# Home-based rehabilitation versus centre-based programs in patients with temporomandibular disorders—a systematic review and meta-analysis

**DOI:** 10.22514/jofph.2024.002

**Published:** 2024-03-12

**Authors:** Jialin Wang, Ruirui Wang, Peng Zhao, Xiao Zhou, Xuanhui Guo

**Affiliations:** ^1^Sports Rehabilitation Research Center, China Institute of Sport Science, 100061 Beijing, China; ^2^College of Sports Medicine and Physical Therapy, Beijing Sport University, 100084 Beijing, China

**Keywords:** Temporomandibular joint disorders, Home care service, Pain intensity, Maximal mouth opening, Meta-analysis

## Abstract

To compare the effects of home-based rehabilitation and occlusal splints or centre-based rehabilitation in patients with temporomandibular joint disorders (TMD). A systematic review and meta-analysis. PubMed, Embase, Cochrane Library, Web of Science and ClinicalTrials.gov electronic databases were consulted from inception to August 2023, searching for randomized controlled trials (RCTs) that compared home-based rehabilitation for TMD with splints or centre-based rehabilitation. The risk of bias was assessed using the Cochrane risk of bias tool. 23 RCTs (1402 participants, three comparator interventions) were identified. Very low-certainty evidence suggested there are no clinically difference between home-based rehabilitation and splints in pain intensity (mean difference (MD) 7.75, 95% confidence interval (CI): 2.17 to 13.32), maximal mouth opening (MMO) (MD 1.83, 95% CI: −0.27 to 3.93) at short and long-term follow-up, in sleep quality (MD: 1.67, 95% CI: −2.04 to 3.56) and quality of life (psychological: MD 0.94, 95% CI: −4.43 to 6.31; general: MD −1.18, 95% CI: −5.72 to 5.37) at short-term follow-up. Low-certainty evidence suggested that home-based rehabilitation plus manual therapy is more effective for TMD treatment compared to home-based rehabilitation at short-term follow-up (pain intensity: MD: 14.93, 95% CI: 7.72 to 21.93; MMO: MD −2.93, 95% CI: −5.3 to −0.54; sleep quality: MD 1.4, 95% CI: 0.09 to 2.71). Compared with home-based rehabilitation, Transcutaneous Electrical Nerve Stimulation (TENS) and Low-Level Laser Therapy (LLLT) was superior in pain relief at short-term follow-up. Low and very low-certainty evidence suggests home-based rehabilitation could be considered a low-cost, beneficial therapy alternative for TMD patients to relieve symptoms.

## 1. Introduction

Temporomandibular disorders (TMD), a musculoskeletal condition characterized by pain dysfunction of the masticatory muscles, the temporomandibular joint (TMJ), and related-tissue components [1], is the 3rd most common chronic pain condition with annual healthcare costs estimated up to $4 billion [2, 3]. In 2014, Eric Schiffman *et al*.’s [4] revised diagnostic criteria for TMD (DC/TMD) divided TMD into two subtypes: pain-related TMD (including myalgia, arthralgia and TMD-induced headache) and intra-articular (including TMJ disc displacement, degenerative arthropathy and subluxation). In 2021, a meta-analysis summarized the prevalence of TMD using DC/TMD as the diagnostic criteria. It concluded that the total prevalence of TMD was about 31% in adults/elderly and about 11% in children/adolescents. In addition, the most common type of TMD is TMJ disc displacement with reduction (DDWR), which accounts for about 26% in adults/older adults and 7.5% in children/adolescents [5].

An international consensus mentioned that unless there are specific and justifiable indications to the contrary, treatment of TMD patients should initially be based on conservative, reversible, and evidence-based therapeutic modalities [6, 7]. Common TMD conservative treatment techniques are centre-based, including physical factor therapy, acupuncture, manual therapy, *etc*. However, the duration of conservative treatment is always continuity a long time. In areas with low economic levels and incomplete coverage of rehabilitation centers, patients may have limited access to rehabilitation. Home-based rehabilitation is a structured program (including exercise training) with clear objectives for the participants, including monitoring, follow-up visits, and letters or telephone calls from staff [8]. Previous studies have pointed out that home rehabilitation is not limited by time, space or equipment and can be done by the patient alone [9, 10, 11, 12].

With the Covid-19 pandemic, home-based rehabilitation became an excellent option for appropriate patients to reduce the risk of viral infection, and the effectiveness has been demonstrated in many studies. Taylor *et al*. [13] found that home-based and center-based rehabilitation approaches appeared to have the same effect on clinical and health-related indicators in cardiac rehabilitation patients. Desheng *et al*. [14] found that home-based rehabilitation was associated with significant improvements in mobility, activities of daily living, instrumental activities, and balance after hip fracture in a study of the effects of home-based rehabilitation in older adults. A meta-analysis by Francesca *et al*. [15] found moderate to low evidence that home-based breathing exercises were near as effective in improving dyspnea, fatigue and mood in Health-related Quality of life (HRQoL) compared to Outpatient treatment.

A review mentioned that the management of patients with TMD should be based on accurate diagnosis and disease-related education and guidance for self-management [16]. Several studies have described the effects of self-management of temporomandibular disorders but lack systematic reviews that provide a comprehensive account of the effectiveness of TMD home rehabilitation. For these reasons, we conducted this review to investigate the efficacy of home-based rehabilitation in reducing signs and symptoms of TMD and compare its results with occlusal splints or centre-based rehabilitation.

## 2. Methods

This meta-analysis (MA) was carried out following the Preferred Reporting Items for Systematic Reviews and Meta-Analysis (PRISMA) guidelines [17]. All analyses were based on published studies. This study was registered in PROSPERO before it started, registration number: CRD42022357572.

### 2.1 Search strategy

We searched the PubMed, Embase, Web of Science and Cochrane Library electronic databases from inception to August 2023. All search strategies were completed by an experienced librarian (WJL) and are summarized in the supplemental material (see **Supplementary material 1**). The reference lists of selected articles were screened for other relevant articles. Grey literature searching included reference lists of included studies and conference proceedings of the following organizations: the American College of Sports Medicine from 2011 to 2023, the American Physical Therapy Association from 2012 to 2023, and the World Confederation for Physical Therapy from 2011 to 2023.

### 2.2 Study selection

First, two authors (WJL and WRR) carried out the screening of titles and abstracts. Second, the same authors checked the full text of the included manuscripts. Third, in case of any disagreement, a decision was made by consensus with the participation of a third author (ZX). A short checklist was adapted to the present review and was used to guide the selection of relevant studies (see **Supplementary material 2**). The exclusion reason and excluded articles are presented in **Supplementary material 2**.

### 2.3 Eligibility criteria

#### 2.3.1 Types of studies

Any published or unpublished randomized controlled trial (RCT) in any language was included.

#### 2.3.2 Types of participants

Patients with symptoms or signs associated with TMD and a diagnosis of TMD were included. Still, studies investigating people with TMD after trauma, pre-post surgery and fracture were excluded.

#### 2.3.3 Types of interventions

Home-based rehabilitation is defined as a structured program (that includes exercise training) with clear objectives for the participants, including monitoring, follow-up visits and letters or telephone calls from staff [8]. In this review, all patient self-management without trained individual (except pre-intervention guidance) involvement was considered home-based rehabilitation. Studies were included whether they were based solely on exercise (such as self-massage and stretching for masticatory and neck muscles, active jaw movement exercises, and so on) or included other intervention elements (comprehensive TMD rehabilitation). Studies in which therapy requiring techniques by a trained individual (such as joint mobilization, passive techniques, some physical factor therapies, and so on) were excluded. The control group mainly received centre-based TMD rehabilitation (*e.g.*, manual therapy, physical factor therapies, *etc*.) or occlusal splints (a splint that could promote correction of the vertical dimension, maxillo-mandibular realignment, TMJ repositioning, and cognitive awareness).

#### 2.3.4 Types of outcome measures

In this review, the researchers collected as much outcome data as possible for meta-analysis. In addition, adverse events were extracted when available. The duration of treatment was defined as [18, 19]:

• short-term (post-treatment to three months),

• intermediate-term (three months up to, but not including, six months), and

• long-term (six months or longer).

### 2.4 Risk of bias assessment in individual studies

Two reviewers (WJL and WRR) independently assessed the risk of bias in included RCTs using the Cochrane Risk of Bias tool [20] which assesses six bias domains: sequence generation; allocation concealment; blinding of participants and personnel, blinding of outcome assessment; incomplete outcome data; selective outcome reporting; other sources of bias (*e.g.*, clarity of diagnosis, compliance to intervention, features of study design). The risk of bias in each domain, and the overall risk of bias for each outcome within a study, were judged as “low”, “high” or “unclear” risk [20]. Trials lacking comprehensive information regarding outcome assessment were judged as unclear risk, and all others low risk, for the domain of detection bias.

### 2.5 Measures

The minimum clinically important difference (MCID) for pain was 19 on a 100-point pain intensity scale [21]. For the MMO, we used an MCID of 6 mm [22, 23, 24]. For other outcomes (*i.e.*, functional disability scales), where there is an absence of clear guidance on MCID and the scales used in different studies are not uniform, we used the common hierarchy of Cohen 1988 [25]: small (0.20), medium (0.50) or large (0.80).

### 2.6 Data extraction

A standardized data extraction form was used, and the following were extracted from all included studies by two authors (WJL and WRR): author names, year of publication, country, number of participants, sex and age of participants, treatment and control interventions, outcome measures, duration of follow-up, results data, side effects. The data extraction form was pilot-tested, and discrepancies in data extraction were resolved by consensus. If the data cannot be meta-analyzed, the results would be described.

### 2.7 Data synthesis

A meta-analysis was performed where possible. STATA (Version 14.0, StataCorp LP, 4905 Lakeway Drive College Station, TX 77845, USA) was used to pool the effect size and conduct sensitivity analysis to explore the source of heterogeneity. To compare the results reported in these studies, we calculated the standardized mean difference (SMD) or weighted mean difference (WMD) and corresponding 95% confidence interval (CIs) for continuous variables and inter-group differences after treatment. SMD was used to standardize the results and eliminate the influence of dimension and measurement methods. Heterogeneity was assessed by the chi-square test [26], and the degree of inconsistency of the treatment effects across trials was measured. Study heterogeneity was evaluated using the inconsistency index (*I*^2^-statistic) with values of 25–50%, 50–75% and 75–100%, indicating low, medium and high heterogeneity, respectively [27]. When significant heterogeneity existed (*I*^2^ ≥ 50%, *p *< 0.1), sensitivity or subgroup analyses were performed to explore the source of heterogeneity. If the cause of heterogeneity could not be identified and the heterogeneity was within the allowed range, a random-effects model was used to pool the effect size [28]. We planned to assess reporting bias using sensitivity analysis and publication bias using the graphical aide funnel plot under the condition of sufficient studies.

The researchers used an adapted the Grading of Recommendations Assessment, Development and Evaluation approach (GRADE) approach, as recommended by the Cochrane Back Review Group to assess the quality of evidence for each outcome data [29]. The quality of the evidence on a specific outcome was based on five main domains: limitations of the study design, inconsistency, indirectness (inability to generalize), imprecision (insufficient or imprecise data) of results, and publication bias across all studies that measured that particular outcome. The quality started at high when at least two RCTs with a low risk of bias provided results for the outcome and were reduced by a level for each of the domains not met.

## 3. Results

### 3.1 Results of the search

23 RCTs met the eligibility criteria [30, 31, 32, 33, 34, 35, 36, 37, 38, 39, 40, 41, 42, 43, 44, 45, 46, 47, 48, 49, 50, 51, 52]. Search results are presented in Fig. 1. The Characteristics of included studies are shown in Table 1. The total number of participants was 1402 (mean age 37.6 years, 81.4% female), and the sample size ranged from 20 to 200. Diagnostic criteria for TMD were RDC/TMD in 16 studies (69.6%). 12 studies [30, 31, 33, 34, 37, 38, 39, 40, 41, 46, 51, 52] compared comprehensive home-based programs (*i.e.*, self-exercise plus education with or without nonsteroidal anti-inflammatory drugs) and the splints. 11 trials [32, 35, 36, 42, 43, 44, 45, 46, 48, 49, 50] compared comprehensive home-based programs with home-based programs plus manual therapy; three trials [39, 40, 41] with electrical nerve stimulation (TENS) or low-level laser therapy (LLLT). The home rehabilitation programs for TMD based on self-care and exercise differed considerably in duration (range: 1 to 12 months), frequency (1 to 5 sessions per week), and session length (20 minutes to 45 minutes per session). Many programs used individually tailored exercise prescriptions, making it difficult to quantify the amount of exercise undertaken precisely. Detailed intervention contents of home-based programs and control groups are shown in **Supplementary Table 3**.

**Fig. 1.** PRISMA Flow Diagram. PRISMA flow diagram showing the process of study selection. 959 articles were retrieved from the bibliographic search, and 4 were found by manual search. 23 articles were included in the review. PRISMA: Preferred Reporting Items of Systematic Reviews and Mata-Analysis; TMDs: Temporomandibular Disorders.

**Table 1.** Demographic characteristics of included studies.

First Author and Year	Treatments and Sample size	Diagnose	Mean Age	Female (%)	Treatment Duration (Weeks)	Outcomes	Follow-up and Loss rate	Country
Truelove 2006	HBR (64) = usual conservative, dentist-prescribed self-care treatment; CG①(68) = hard splint; CG②(68) = soft splint.	RDC/TMD^3^	36.0 (SD 12.0)	88.6%	12	A, B, C, D, E	3 and 12 months (30%)	America
Haketa 2010	HBR (19) = self-exercise treatment; CG (21) = splint.	MRI^2^	37.6 (SD 14.9)	88.5%	8	B, F, G	4 and 8 weeks (11.5%)	Japan
Ficnar 2013	HBR (21) = self-exercises; CG①(21) = semi-finished occlusal appliance; CG②(21) = laboratory-made occlusal appliance.	RDC/TMD^3^	18∼50	79.4%	12	B, H	3 months (8%)	Germany
Costa 2015	HBR (30) = counseling for behavioral changes; CG (30) = splint.	RDC/TMD^1^	31.6 (SD 7.9)	90.0%	NM	I	2 and 5 months (31.2%)	Brazil
de Resende 2019	HBR (19) = counseling; CG①(25) = counseling + splint; CG②(24) = splint; CG③(21) = manual therapy.	RDC/TMD^3^	18∼61	80.1%	4	F, J, K, R	1 month (4%)	Brazil
Melo 2020	HBR (19) = counseling; CG①(25) = counseling + splint; CG②(24) = splint; CG③(21) = counseling + manual therapy.	RDC/TMD^3^	18∼65	NM	4	F, S, T, U	1 month (23%)	Brazil
Wanman 2020	HBR (30) = home exercise; CG (30) = splint.	RDC/TMD^2^	38.7 (SD 15.1)	70.0%	6	B, E, V, X	3 months (16.7%)	Sweden
Peixoto 2021	HBR (15) = counseling; CG①(15) = splint; CG②(15) = scalp acupuncture; CG③(15) = manual therapy.	RDC/TMD^3^	18∼65	NM	4	F, J, K	1 month (35%)	Brazil
Craane 2012	HBR (26) = counseling for behavioral changes; CG (23) = HBR + manual therapy.	RDC/TMD^2^	36.7 (SD 14.6)	95.9%	6	B, D, F, Y, Z	3, 6, 12, 26, 52 weeks (14.3%)	Netherlands
Niemelä 2012	HBR (37) = patient education and self-exercise; CG (39) = splint.	RDC/TMD^3^	43.7 (SD 13.1)	81.6%	4	B, F, H	4 weeks (5.2%)	Finland
Tuncer 2013①	HBR (20) = patient education and self-exercise; CG (20) = HBR + manual therapy.	RDC/TMD^3^	35.9 (SD 13.4)	77.5%	4	B, F	4 weeks (0%)	Turkey
Tuncer 2013②	HBR (20) = patient education and self-exercise; CG (20) = HBR + manual therapy.	RDC/TMD^3^	35.9 (SD 13.4)	77.5%	4	FHP	4 weeks (0%)	Turkey
Qvintus 2015	HBR (40) = patient education and self-exercise; CG (39) = stabilization splint.	DC/TMD^3^	43.3 (SD 13.2)	77.5%	48	F	12 months (22.5%)	Finland
Cavalcanti 2016	HBR (20) = self-care and anti-inflammatory drug; CG (20) = LLLT.	Muscle tenderness palpation and Fonseca^3^	25∼50	100%	4	B, PPD	1, 2, 3, 4 weeks (0%)	Brazil
Machado 2016	HBR (22) = patient education and oral motor exercises; CG (18) = TENS.	RDC/TMD^3^	33.5 (SD 11.9)	92.7%	16	ProTMDmulti, TP, OMES	3 months (21.2%)	Brazil
Patil 2017	HBR (18) = patient education and self-exercise; CG (18) = LLLT.	RDC/TMD^3^	33.5 (SD 10.2)	63.8%	4	B, F, TP	1, 2, 3, 4 weeks (0%)	Saudi Arabia
Corum 2018	HBR (18) = patient education and self-exercise; CG (18) = HBR + manual therapy.	RDC/TMD^3^	27.9 (SD 7.0)	100%	6	B, D, F, SF-36	1 month (8%)	Turkey
Nagata 2018	HBR (30) = patient education and self-exercise; CG (31) = HBR + manual therapy.	DC/TMD^2^	49.6 (SD 25.0)	82.0%	NM	B, C, F	From 2 to 18 weeks after baseline (21%)	Japan
Brandão 2020	HBR (8) = patient education; CG (11) = HBR + manual therapy.	RDC/TMD^3^	18∼60	NM	4	PPD	1 month (17%)	Brazil
Delgado 2020	HBR (30) = patient education and exercise; CG (31) = HBR + manual therapy.	RDC/TMD^3^	43.2 (SD 11.2)	59%	4	B, F, SF-36, THI, BDI, CF-PDI	4 weeks and 3, 6 month (8.2%)	Spain
Ram 2021	HBR (40) = patient education and self-exercise; CG①(40) = stabilization splints; CG②(40) = HBR + manual therapy.	DC/TMD^3^	39.4 (SD 10.3)	54.4%	NM	B, F	1, 2 weeks and 1, 3 month (8.8%)	India
Gikić 2021	HBR (15) = patients education and home-based exercise; CG①(15) = stabilisation splint; CG②(15) = other occlusal devices.	DC/TMD^3^	36.45 (SD 10.51)	100%	24	F, B, R, V, PSS, GAD-7	3 and 6 months (33.3%)	Croatia
Olbort 2023	HBR (30) = home-based exercise; CG (30) = stabilisation splint.	DC/TMD^3^	49.35 (SD16.34)	70%	24	B, F, C	2, 4 and 6 months (0%)	Germany

*HBR: home-based rehabilitation; ①②③: the study had more than two groups, which were distinguished by the sequence number ①, ② and ③; CG: control group; SD: standard deviation; TMD: temporomandibular disorders; RDC/TMD: Research Diagnostic Criteria for temporomandibular disorders; MRI: Magnetic Resonance Imaging; DC/TMD: Diagnostic Criteria for temporomandibular disorders; TMJ: temporomandibular joint; ^1^: masticatory myofascial pain; ^2^: TMJ disc displacement; ^3^: the mixed TMD; NM: not mentioned; A: the mean of present, average and worst TMD-related pain in the past two months (CPI); B: maximum mouth opening (MMO); C: Joint sounds; D: pressure pain sensitivity (PPT); E: RDC/TMD diagnoses; F: pain (NPRS/VAS); G: Limitation of Daily Functions for the TMD Questionnaire (LDFTQ); H: the number of pressure-sensitive areas of the TMJ; I: TMD-related headache characteristics and frequency using International Classification of Headache Disorders (ICHD); J: sleep quality-SQ (PSQI); K: Quality of life-QL (WHOQOL-BREF); R: quality of life related to oral health-QLOH (OHIP-14); S: Hospital Anxiety and Depression Scale (HADS); T: Beck Anxiety Inventory (BAI); U: the State-Trait Anxiety Inventory (STAI-S and T); V: Jaw function limitation scale-20 (JFLS-20); W: Neck Disability Index (NDI); X: the rating of the subject’s motivation to complete the intervention (NRS); Y: McGill Pain Questionnaire (MPQ); Z: Mandibular Function Impairment Questionnaire (MFIQ); FHP: Forward head posture (degrees); SF-12/36: general health-related quality of life; THI: Tinnitus Handicap Inventory; CF-PDI: TMD-related disability; BDI-Ⅱ: Beck Depression Inventory; PPD: the percentage of pain and depression (%); LLLT: low-level laser irradiation; Fonseca: the questionnaire of Fonseca; TENS: transcutaneous electrical nerve stimulation; ProTMDmulti: Self-judgment of TMD severity; TP: Tenderness to palpation (NRS); OMES: Orofacial myofunctional status; PSS: Perceived Stress Scale; GAD-7: The Generalized Anxiety Disorder-7.*

### 3.2 Risk of bias assessment

The included studies had a high risk of bias in subject blindness and attrition. 16 studies did not blind participants [30, 31, 34, 35, 36, 37, 38, 39, 40, 41, 43, 44, 45, 47, 48], and four studies did not report whether participants were blinded [32, 33, 42, 46]. these studies were at high risk for performance bias. Seven trials [33, 34, 35, 36, 39, 41, 42], which did not report whether outcome assessors were blinded, were at unclear risk of detection bias. Three trials [38, 41, 52] was at high risk for detection bias. Ten trials [32, 37, 38, 40, 42, 43, 45, 46, 49, 50] had high attrition rates and were at high risk of attrition bias. 13 studies [33, 34, 35, 36, 38, 39, 40, 41, 42, 44, 46, 47, 48] did not report whether allocation hiding was performed (Fig. 2 and **Supplementary Fig. 1**).

**Fig. 2.** Risk of bias summary. Review authors’ judgments about each risk of bias item for each included study (Risk of Bias scale); ①②: the study had three groups, which were distinguished by the sequence number.

### 3.3 Effects of interventions

#### 3.3.1 Home-based rehabilitation versus occlusal splints

Seven trials were available for this comparison, including 391 patients with TMD [31, 33, 48, 49, 50, 51, 52]. The maximum duration of follow-up ranged from four weeks to one year, and the mean age of the participants ranged from 18 to 65 years. The comparison results were shown in Fig. 3.

**Fig. 3.** Forest plot comparing home-based rehabilitation with splints. Pooled mean differences calculated by random effects model in pain, maximal mouth opening and sleep quality, fixed effects model in quality of life.

##### 3.3.1.1 Pain intensity

Short-termed improvements in current average pain intensity using VAS/NPRS were reported in all seven studies [31, 33, 48, 49, 50, 51, 52]. The results showed that the splint group relieved pain intensity more significantly than the home-based rehabilitation (WMD 7.75, 95% CI: 2.17 to 13.32, participants = 391; studies = 7 (11 comparisons); *I*^2^ = 80.4%; random-effect; very low-quality evidence; **Supplementary Fig. 2**). Long-termed improvements in pain relief were reported in four studies [30, 38, 51, 52], and one of them reported that the splints group was better than the home-based rehabilitation group (**Supplementary Fig. 3**) [52]. There was no evidence of funnel plot asymmetry for pain intensity (Egger test *p* = 0.65; **Supplementary Fig. 4**). Sensitivity analyses were conducted to investigate each study’s influence on the overall risk estimate by omitting one study and found that no study changed the pooled outcome (**Supplementary Fig. 5**).

##### 3.3.1.2 Maximal mouth opening (MMO)

Short-termed improvement in MMO was measured in five studies [31, 33, 50, 51, 52], but no significant differences in MMO improvement were observed between home-based rehabilitation and splint (WMD 1.83, 95% CI: 0.27 to 3.93, participants = 298; studies = 5 (8 comparisons); *I*^2^ = 86.0%; random-effect; very low-quality evidence, **Supplementary Fig. 6**). Ficnar *et al*. [34] reported that the splint group improved MMO more significantly than the home-based rehabilitation at 2.5 months. Truelove *et al*. [30] compared the splint treatment of two materials with home-based rehabilitation. They found neither splint therapy provided a more significant medium-term benefit than self-care treatment without splint therapy. Long-termed improvements in MMO were reported in two studies with three comparisons and the results indicated that home-based rehabilitation group was better than splints group (WMD 4.50, 95% CI: 0.11 to 8.89, *I*^2^ = 89.4%; random-effect, **Supplementary Fig. 7**).

##### 3.3.1.3 Sleep quality

Two trials [48, 49] with a high risk of bias investigated short-term effects of sleep quality using a Sleep Quality Index (PSQI), showing there were no statistical differences in sleep quality for patients randomized to either home-based rehabilitation or splints group at short-term (WMD 1.67, 95% CI: −0.24 to 3.56, participants = 114; studies = 2 (3 comparisons); *I*^2^ = 68.0%; random-effect; low-quality evidence, **Supplementary Fig. 8**).

##### 3.3.1.4 Quality of life (QL)

Two studies [48, 49] evaluated QL (psychological, social, general) using the World Health Organization QL (WHOQOL-Bref). The pooled effects did not show statistical differences in psychological (WMD 0.94, 95% CI: −4.43 to 6.31, *I*^2^ = 0%, **Supplementary Fig. 9**) and general (WMD −0.18, 95% CI: −5.72 to 5.37, *I*^2^ = 0%, **Supplementary Fig. 10**) part of QL, but the home-based rehabilitation showed a worsening in the social part of quality life (WMD −10.76, 95% CI: −17.00 to −4.52, participants = 114; studies = 2 (3 comparisons); *I*^2^ = 0%; fixed-effect; low-quality evidence, **Supplementary Fig. 11**).

#### 3.3.2 Home-based rehabilitation versus home-based rehabilitation plus-manual therapy

Ten trials [32, 36, 42, 43, 44, 45, 46, 48, 49, 50] were available for this comparison and included 445 patients with TMD. The maximum duration of follow-up ranged from four weeks to one year, and the mean age of the participants ranged from 28.8 to 50.7 years. The results were detailed in Fig. 4.

**Fig. 4.** Forest plot comparing home-based rehabilitation with home-based rehabilitation plus manual therapy. Pooled mean differences were calculated by random effects model in pain, maximal mouth opening and pressure pain thresholds, and fixed effects model in function, sleep quality, quality of life, and depression symptoms.

##### 3.3.2.1 Pain intensity

Short-termed improvements in current average pain intensity using VAS/NPRS were reported in ten studies [32, 36, 42, 43, 44, 45, 46, 48, 49, 50]. The pooled result showed home-based rehabilitation plus manual therapy has a better impact on pain relief than only home-based rehabilitation (WMD 14.93, 95% CI: 7.92 to 21.93, participants = 365; studies = 8 (11 comparisons); *I*^2^ = 76.6%; random-effect; low-quality evidence; **Supplementary Fig. 12**). Brandão and Nagata *et al*. [43, 44] reported pain relief in both groups, but no between-group difference existed. Two studies [32, 45] reported intermediate to long-term impacts in pain intensity, but their results were highly heterogeneous (*I*^2^ = 94.8%). One showed manual therapy has additional effects on pain relief [45], while the other did the opposite [32]. There was no evidence of funnel plot asymmetry for pain intensity (Egger test *p* = 0.1; **Supplementary Fig. 13**). Sensitivity analyses were conducted to investigate each study’s influence on the overall risk estimate by omitting one study and found that no study changed the pooled outcome (**Supplementary Fig. 14**).

##### 3.3.2.2 Maximal mouth opening (MMO)

Five trials [32, 36, 42, 43, 50] reported MMO at short-term showing home-based rehabilitation plus manual therapy has a better impact on MMO improvement than only home-based rehabilitation (WMD −2.93, 95% CI −5.3 to −0.54; participants = 198; studies = 4 (8 comparisons); *I*^2^ = 81.7%; random-effect; low-quality evidence; **Supplementary Fig. 15**). Nagata *et al*. [43] reported that additional manual therapy was superior to only home rehabilitation after the first treatment; the mean average increment was 6.86 mm (95% CI 8.12 to 5.55). In contrast, no significant differences were observed after the second visit (*p *> 0.05). Two studies [32, 45] reported intermediate-term effects, but their results were highly heterogeneous (*I*^2^ = 93.0%). One [45] showed manual therapy has an additional impact on MMO improvement than only home-based rehabilitation in mixed TMD patients (WMD −5.1, 95% CI −7.04 to −3.16), while the other [32] found no evidence of a significant difference between the two management in disc displacement without reduction (DDWOR) patients (WMD = 3.7, 95% CI −0.43 to 7.83). Two studies [32, 45] reported MMO in the long-term follow-up, but their results were highly heterogeneous (*I*^2^ = 94.5%). One [45] showed additional manual therapy brought a better effect on MMO improvement in the mixed TMD patients (WMD −6.50, 95% CI −8.27 to −4.73), while the other [32] found no evidence of a significant difference between the two management in DDWOR patients (WMD = 3.40, 95% CI −0.78 to 7.58).

##### 3.3.2.3 Pressure pain thresholds (PPT)

Three studies reported short-term improvements in PPT [32, 42, 45], and the results showed that there was a statistical difference between home rehabilitation and manual therapy plus home rehabilitation in changes in PT-temporal muscle (SMD −0.66, 95% CI −1.31 to −0.01; participants = 146; studies = 3 (6 comparisons); *I*^2^ = 85.0%; random-effect; low-quality evidence, **Supplementary Fig. 16**), but not in PPT-masseter (SMD −0.57, 95% CI −1.14 to 0.01; participants = 146; studies = 3 (6 comparisons); *I*^2^ = 81.1%; random-effect; low-quality evidence, **Supplementary Fig. 17**). Two studies whose results were inconsistent reported intermediate and long-term improvement in PPTs. Delgado *et al*. [45] indicated that individuals receiving home-based rehabilitation plus manual therapy showed more significant increases (large effect sizes) in PPTs than those receiving home rehabilitation alone. Craane *et al*. [32] found no evidence of a statistically significant difference in PPTs between the two groups.

##### 3.3.2.4 Function

Two studies [32, 45] using Mandibular Function Impairment Questionnaire (MFIQ) and Craniofacial Pain and Disability Index (CF-PDI) reported function improvement. Craane *et al*. [32] reported no statistically significant difference in MFIQ scores between home-based rehabilitation and manual therapy plus home-based rehabilitation in DDWOR patients (SMD −0.20, 95% CI −0.53 to 0.13; *I*^2^ = 0%, **Supplementary Fig. 18**). Delgado *et al*. [45] indicated that additional manual therapy significantly improved CF-PDI more than home rehabilitation alone in mixed TMD patients (SMD 0.59, 95% CI 0.07 to 1.10).

##### 3.3.2.5 Sleep quality

Two trials with a high risk of bias [48, 49] evaluated sleep quality in the short-term using PSQI showing additional manual therapy has a better effect compared with only home-based rehabilitation in sleep quality improvement (WMD 1.40, 95% CI 0.09 to 2.71, participants = 70; studies = 2; *I*^2^ = 0.0%; fixed-effect; low-quality evidence, **Supplementary Fig. 19**).

##### 3.3.2.6 Quality of life (QL)

Two trials with a high risk of bias [48, 49] using the WHOQOL-Brief evaluated QL (psychological, social, general) at short-termed follow-up. The pooled result showed there were no statistically significant differences in psychological (WMD −2.75, 95% CI: −8.88 to 3.38, *I*^2^ = 0%, **Supplementary Fig. 20**), social (WMD −7.1, 95% CI: −14.84 to 0.64, *I*^2^ = 0%, **Supplementary Fig. 21**) and general (WMD −2.45, 95% CI: −9.19 to 4.28, *I*^2^ = 0%, **Supplementary Fig. 22**) part of QL.

#### 3.3.3 Home-based rehabilitation versus TENS or LLLT

Three studies [39, 40, 41] describe the efficacy of home-based rehabilitation versus TENS or LLLT. The maximum duration of follow-up ranged from four weeks to one year, and the mean age of the participants (n = 116) ranged from 32.91 to 50.0 years. The comparison results were shown in Fig. 5.

**Fig. 5.** Forest plot comparing home-based rehabilitation with TENS or LLLT. Pooled mean differences calculated by a fixed effects model in tenderness to palpation.

One study [41] reported short-term TP using VAS, showing only a statistically significant difference in favor of TENS compared with home-based rehabilitation in TP-masseter and TMJ (masseter: WMD 10.00, 95% CI: 4.69 to 15.32, *I*^2^ = 20.5%, fixed-effect; TMJ: WMD = 11.05, 95% CI: 2.74 to 19.37, *I*^2^ = 58.1%, random-effect, **Supplementary Figs. 23,24**). The pooled effect size of TP-temporalis (WMD 3.31, 95% CI −2.92 to 9.53, *I*^2^ = 0.0%, fixed-effect, **Supplementary Fig. 25**) showed no significant differences in short-term effects between the two groups. In addition, one low-quality study [39] found that compared with home-based rehabilitation, patients in the LLLT group significantly reduced the proportion of patients in pain after treatment (LLLT: 75%, Home: 55%). One study [40] reported TP at intermediate-term, showing a significant difference in favor of home-based rehabilitation compared with LLLT in TP-masseter and TMJ (masseter: WMD −1.60, 95% CI: −2.68 to −0.52, *I*^2^ = 0.0%, fixed-effect; TMJ: WMD −1.55, 95% CI: −2.68 to −0.43, *I*^2^ = 0.0%, fixed-effect, **Supplementary Figs. 26,27**), but there were no significant differences in TP-temporalis (WMD 3.31, 95% CI −2.92 to 9.53, *I*^2^ = 0.0%, fixed-effect).

### 3.4 Cost-effectiveness

Since all original literature does not list the cost of treatment, we have no way to calculate the exact cost-effectiveness. We have summarized the treatment items that may incur costs in the included trials and presented them in **Supplementary Table 4**. These studies are from different countries, and we cannot list medical expenses.

### 3.5 Adverse events

Only two of the 23 studies reported whether there were adverse events during the trial. Cavalcanti and Gikić *et al*. [39, 51] reported no patients in the actual study reported any side effects.

## 4. Discussion

### 4.1 Summary

This systematic review and meta-analysis included 23 studies on TMD home-based rehabilitation. (1) There was low to very low-certainty evidence that no statistical difference between home-based rehabilitation and splints in MMO, sleep and quality of life (psycho and general) improvement at short-term follow-up. There was low to very low-certainty evidence that home-based rehabilitation is inferior to splints in improving pain intensity and quality of life (social) at short-term follow-up. Home rehabilitation was superior to splinting in terms of long-term improvement in MMO. However, the two groups’ statistical differences in pain and MMO improvement were not clinically significant. (2) There was low to very low-certainty evidence that home-based rehabilitation plus manual therapy was superior to only home-based rehabilitation on pain relief, MMO, PPT-temporalis, and sleep quality improvement at short-term follow-up. However, the two groups’ statistical differences in pain and MMO improvement were not clinically significant. Very low-certainty evidence indicated no statistically significant differences in functional status, PPT-masseter, and quality of life improvement at short-term follow-up and no statistically significant differences in MMO improvement at intermediate and long-term follow-up. (3) In comparing home-based rehabilitation and TENS or LLLT, all evidence is very low certainty. TENS and LLLT were superior to home-based rehabilitation in TP-TMJ and masseter in short-term follow-up. In intermediate-term follow-up, the results showed patient’s self-management was superior to LLLT in TP-TMJ and masseter.

### 4.2 Analysis of Home-based rehabilitation

The conclusion is of practical significance: TMD can be treated at home with no clinically significant difference in pain, function or quality of life improvement compared with other conservative treatments, such as splints or centre-based rehabilitation.

In a short-term follow-up comparing the efficacy of home-based rehabilitation and splint therapy for TMD patients, it was found that, after the exclusion of studies item by item, the study by Haketa *et al*. [31] contributed significantly to the heterogeneity of the pooled results (*I*^2^ = 80.4%). The reason for this result may be that the study focused on patients with temporomandibular disc displacement without reduction (ADDWOR). The effectiveness of splinting for TMD patients has always received attention, but the outcomes remain controversial. In 2020, Fouda *et al*. [53] published a meta-analysis showing that splints are not effective in reducing pain intensity or improving function in patients with TMD. In 2021, Zhang *et al*. [54] reported in a systematic review including six studies that both exercise and splint therapy are effective treatment methods for TMD patients, but the difference in effectiveness between the two remains unclear, which is consistent with our meta-analysis results. In 2020, Fernández *et al*. [55] pointed out that the clinical effects of the same treatment may be related to different TMD subtypes. A pre- and post-test clinical trial indicated that exercise seems effective for TMD patients with anterior disc displacement [56]. A randomized controlled trial compared the therapeutic effects of centric and distraction splints on ADDWOR patients, finding that both types of splints could significantly improve maximum mandibular opening and reduce subjective pain [57]. A review of the utilization of splints for TMD determined that the underlying mechanism and effectiveness of splint therapy continue to require comprehensive comprehension [58]. Additionally, Ram and Niemelä *et al*.’s [33, 50] study also home-educated patients in the splint group, which may bring additional benefits to the splint group and is a source of heterogeneity in synthetic outcomes.

Quality of life, measured by the WHOQOL-General, showed improvement in the general QL and the other domains after home-based rehabilitation and splint treatment. However, in the social domains, the home-based rehabilitation showed an inverse line to the splints, showing a worsening with QL. Peixoco and de Resende [48, 49] suggested that this result may be because the guiding of home rehabilitation proposed in the study does not cover the components of the social domain. However, the small sample size (two studies included 114 TMD patients) and poor methodological quality of the included original research can lead to lower certainty in the results (uncertainties of allocation concealment and high risk of blind methods). Therefore, this result is also likely due to a bias. It should be noted that in the medium to long-term follow-up, the clinical effectiveness of home-based rehabilitation may be unstable. This result might be due to decreased compliance after three months in the home rehabilitation group [59]. In 2021, Beverley Kok *et al*. [60] analyzed the improvement of physical function in patients with cirrhosis by home exercise and found that the benefits of exercise largely depend on continued participation, which supported our result [61].

Manual therapy has been used to restore a normal range of motion, reduce local ischemia, stimulate proprioception, break fibrous adhesions, stimulate synovial fluid production, and reduce pain [62, 63, 64, 65]. Among the included studies, the content of manual therapy mainly included: massage, joint mobilization, muscle energy technique, and myofascial release techniques. In the short-term follow-up, home-based rehabilitation plus manual therapy was more effective in improving pain intensity, MMO, the PPT-temporalis muscle, sleep quality, and depressive symptoms than only home-based rehabilitation. A meta-analysis showed that based on the low-quality certainty evidence, manual therapy shows promising results in treating myogenous, arthrogenous and mixed TMD [63]. However, this requires a high degree of therapist expertise and is difficult to implement in home-based rehabilitation, which may be one of the reasons for the superiority of additional manual therapy. In addition, there was little difference in the improvement of sleep quality and depressive symptoms between home-based rehabilitation and manual treatment; as Reid and Hofmann *et al*. [66, 67], these two indicators are difficult to change in the short term. In the interim follow-up results of the MMO, two studies showed different outcomes in TMD patients with additional manual therapy, possibly due to differences in the subtypes of TMD in the enrolled patients. Delgado *et al*. [45] included patients with mixed TMD, while Craane *et al*. [33] included patients with TMJ disc displacement. In 2020, Fernández *et al*. [55] proposed that differences in clinical outcomes due to manual therapy may be related to different TMD subgroups—such as myofascial or arthrogenic; Therefore, it is crucial to determine which TMD subtypes require which particular manual therapy.

Compared with TENS and LLLT, the short-term follow-up results of both TP-masseter and TP-TMJ showed that home-based rehabilitation was inferior to the control group. Still, the intermediate follow-up results were the opposite. In 2017, the result of a meta-analysis that showed that LLLT treatment has only short-term effects on pain relief in orofacial regions was consistent with our review’s results [68].

Possible reasons for the effectiveness of home rehabilitation include the possibility of higher attendance, independent of weather, traffic and venue. Patient attendance is essential to ensuring recovery outcomes in treating chronic musculoskeletal disorders with a lengthy recovery period. Essery R *et al*. [69], in their study of predictors of home-based rehabilitation, found that patients had high intrinsic motivation and that interest produced more sustained performance than extrinsic motivation (stimulation by rehabilitation therapists). Christiansen *et al*. [70] conducted a multicentre RCT to compare the efficacy of group and home exercise in patients with subacromial pain. The results suggest that home rehabilitation may lead to more exercise time. After collecting and comparing the treatment equipment needed by the home-based rehabilitation and the control group in the included study, it was found that the treatment cost of home-based rehabilitation was lower than that of the other control groups in this review when the treatment effects were not inferior to that of the control group.

### 4.3 Strengths and limitations

This is the first meta-analysis to compare the effect of home-based rehabilitation and occlusal splints or centre-based rehabilitation in patients with TMD. The rigorous research methodology used in this study involved two experienced researchers who performed the tasks of article selection, data extraction and quality evaluation. We referenced accepted guidelines and included as many databases as possible, with no restrictions on language or year of publication in the selection. However, relatively significant clinical and statistical heterogeneity resulted in the quality of our results turning out to be low to very low certainty.

Due to the number of research being insufficient, we are unable to discuss the type of manual therapy and the subtype of TMD, which may be one of the essential sources of the risk of bias in this study, and future systematic reviews should be more nuanced with subgroup analysis to provide more direct guidance for clinical practice. As with the limitations of all home rehabilitation, we cannot tell if all patients completed the established home rehabilitation components on time and in the right amount. Although there was no restriction on the languages included in the study, we did not search the literature base for other languages, which could lead to a risk of bias. The studies in this review came from various countries, with significant variation between national rehabilitation facilities and economic levels. Differences in rehabilitation facilities and costs between developed and developing countries may affect compliance, outcomes, and patient expectations in home-based rehabilitation.

### 4.4 Clinical implication

Although our study yielded only low to very low-certainty evidence, due to the homogeneity of the final results, we believe that home-based rehabilitation is an option for TMD patients when splints or centre-based rehabilitation are unavailable.

## 5. Conclusion

Low and very low-certainty evidence suggested the efficiency of home-based rehabilitation for treating TMD patients, when compared with occlusal splints, arthrocentesis, and TENS or LLLT, has similar results in pain relief, function and quality of life improvement. The additional manual therapy could provide better short-term pain relief and MMO improvement results than home-based rehabilitation alone. Further studies with higher levels of evidence and more representative samples are needed to verify the performance of this form of treatment.

## 6. Key findings

In this systematic review and meta-analysis that included 23 trials, low and very low certainty evidence suggests home-based rehabilitation could be considered a conservative, low-cost, beneficial therapy alternative for TMD patients to relieve symptoms.

## Supplementary Material




