# Supplementary material for: Home-based rehabilitation versus centre-based programs in patients with temporomandibular disorders—a systematic review and meta-analysis
Source: J Oral Facial Pain Headache. 2024 Mar 12;38(1):1–16. doi: 10.22514/jofph.2024.002 (PMC11798648; doi:10.22514/jofph.2024.002)
Supplement: Supplementary file 1 [file Supplementary-material-1.docx]

Supplementary material

Database: Pubmed (As of 16 August 2023).

| Search | Query | Results | Time |
| --- | --- | --- | --- |
| #17 | Search: #3 AND #15 Filters:  Clinical Trial, Randomized Controlled Trial | 253 | 22:37:17 |
| #16 | Search: #3 AND #15 | 1790 | 22:36:16 |
| #15 | Search: #4 OR #5 OR #6 OR #7 OR #8 OR #9 OR #10 OR #11 OR #12 OR #13 OR #14 | [567,862](https://pubmed.ncbi.nlm.nih.gov/?term=%234+OR+%235+OR+%236+OR+%237+OR+%238+OR+%239+OR+%2310+OR+%2311+OR+%2312+OR+%2313+OR+%2314&sort=) | 22:35:52 |
| #14 | Search: ((((((((((((((((((“Manipulations, Musculoskeletal”[Title/Abstract]) OR (“Manipulation Therapy”[Title/Abstract])) OR (“Manipulative Therapies”[Title/Abstract])) OR (“Manipulative Therapy”[Title/Abstract])) OR (“Therapies, Manipulative”[Title/Abstract])) OR (“Therapy, Manipulative”[Title/Abstract])) OR (“Therapy, Manipulation”[Title/Abstract])) OR (“Manipulation Therapies”[Title/Abstract])) OR (“Therapies, Manipulation”[Title/Abstract])) OR (“Reflexology”[Title/Abstract])) OR (“Bodywork”[Title/Abstract])) OR (“Bodyworks”[Title/Abstract])) OR (“Rolfing”[Title/Abstract])) OR (“Craniosacral Massage”[Title/Abstract])) OR (“Massage, Craniosacral”[Title/Abstract])) OR (“Manual Therapies”[Title/Abstract])) OR (“Manual Therapy”[Title/Abstract])) OR (“Therapies, Manual”[Title/Abstract])) OR (“Therapy, Manual”[Title/Abstract]) | [5526](https://pubmed.ncbi.nlm.nih.gov/?term=%28%28%28%28%28%28%28%28%28%28%28%28%28%28%28%28%28%28%22Manipulations%2C+Musculoskeletal%22%5BTitle%2FAbstract%5D%29+OR+%28%22Manipulation+Therapy%22%5BTitle%2FAbstract%5D%29%29+OR+%28%22Manipulative+Therapies%22%5BTitle%2FAbstract%5D%29%29+OR+%28%22Manipulative+Therapy%22%5BTitle%2FAbstract%5D%29%29+OR+%28%22Therapies%2C+Manipulative%22%5BTitle%2FAbstract%5D%29%29+OR+%28%22Therapy%2C+Manipulative%22%5BTitle%2FAbstract%5D%29%29+OR+%28%22Therapy%2C+Manipulation%22%5BTitle%2FAbstract%5D%29%29+OR+%28%22Manipulation+Therapies%22%5BTitle%2FAbstract%5D%29%29+OR+%28%22Therapies%2C+Manipulation%22%5BTitle%2FAbstract%5D%29%29+OR+%28%22Reflexology%22%5BTitle%2FAbstract%5D%29%29+OR+%28%22Bodywork%22%5BTitle%2FAbstract%5D%29%29+OR+%28%22Bodyworks%22%5BTitle%2FAbstract%5D%29%29+OR+%28%22Rolfing%22%5BTitle%2FAbstract%5D%29%29+OR+%28%22Craniosacral+Massage%22%5BTitle%2FAbstract%5D%29%29+OR+%28%22Massage%2C+Craniosacral%22%5BTitle%2FAbstract%5D%29%29+OR+%28%22Manual+Therapies%22%5BTitle%2FAbstract%5D%29%29+OR+%28%22Manual+Therapy%22%5BTitle%2FAbstract%5D%29%29+OR+%28%22Therapies%2C+Manual%22%5BTitle%2FAbstract%5D%29%29+OR+%28%22Therapy%2C+Manual%22%5BTitle%2FAbstract%5D%29&sort=) | 22:34:32 |
| #13 | Search: “Musculoskeletal Manipulations”[Mesh] Sort by: Most Recent | [18,706](https://pubmed.ncbi.nlm.nih.gov/?sort=date&term=%22Musculoskeletal+Manipulations%22%5BMesh%5D) | 22:31:48 |
| #12 | Search: (“Home Health Care Nursing”[Title/Abstract]) OR (“Nursing, Home Health”[Title/Abstract]) | [94](https://pubmed.ncbi.nlm.nih.gov/?term=%28%22Home+Health+Care+Nursing%22%5BTitle%2FAbstract%5D%29+OR+%28%22Nursing%2C+Home+Health%22%5BTitle%2FAbstract%5D%29&sort=) | 22:31:05 |
| #11 | Search: “Home Health Nursing”[Mesh] Sort by: Most Recent | [377](https://pubmed.ncbi.nlm.nih.gov/?sort=date&term=%22Home+Health+Nursing%22%5BMesh%5D) | 22:30:40 |
| #10 | Search: ((((((((((((((((((“Modalities, Physical Therapy”[Title/Abstract])) OR (“Modality, Physical Therapy"[Title/Abstract])) OR (“Physical Therapy Modality”[Title/Abstract])) OR (“Physiotherapy (Techniques)”[Title/Abstract])) OR (“Physiotherapies (Techniques)”[Title/Abstract])) OR (“Physical Therapy Techniques”[Title/Abstract])) OR (“Physical Therapy Technique”[Title/Abstract])) OR (“Techniques, Physical Therapy”[Title/Abstract])) OR (“Group Physiotherapy”[Title/Abstract])) OR (“Group Physiotherapies”[Title/Abstract])) OR (“Physiotherapies, Group”[Title/Abstract])) OR (“Physiotherapy, Group”[Title/Abstract])) OR (“Physical Therapy”[Title/Abstract])) OR (“Physical Therapies”[Title/Abstract])) OR (“Therapy, Physical”[Title/Abstract])) OR (“Neurological Physiotherapy”[Title/Abstract])) OR (“Physiotherapy, Neurological”[Title/Abstract])) OR (“Neurophysiotherapy”[Title/Abstract]) | [28,378](https://pubmed.ncbi.nlm.nih.gov/?term=%28%28%28%28%28%28%28%28%28%28%28%28%28%28%28%28%28%28%22Modalities%2C+Physical+Therapy%22%5BTitle%2FAbstract%5D%29%29+OR+%28%22Modality%2C+Physical+Therapy%22%5BTitle%2FAbstract%5D%29%29+OR+%28%22Physical+Therapy+Modality%22%5BTitle%2FAbstract%5D%29%29+OR+%28%22Physiotherapy+%28Techniques%29%22%5BTitle%2FAbstract%5D%29%29+OR+%28%22Physiotherapies+%28Techniques%29%22%5BTitle%2FAbstract%5D%29%29+OR+%28%22Physical+Therapy+Techniques%22%5BTitle%2FAbstract%5D%29%29+OR+%28%22Physical+Therapy+Technique%22%5BTitle%2FAbstract%5D%29%29+OR+%28%22Techniques%2C+Physical+Therapy%22%5BTitle%2FAbstract%5D%29%29+OR+%28%22Group+Physiotherapy%22%5BTitle%2FAbstract%5D%29%29+OR+%28%22Group+Physiotherapies%22%5BTitle%2FAbstract%5D%29%29+OR+%28%22Physiotherapies%2C+Group%22%5BTitle%2FAbstract%5D%29%29+OR+%28%22Physiotherapy%2C+Group%22%5BTitle%2FAbstract%5D%29%29+OR+%28%22Physical+Therapy%22%5BTitle%2FAbstract%5D%29%29+OR+%28%22Physical+Therapies%22%5BTitle%2FAbstract%5D%29%29+OR+%28%22Therapy%2C+Physical%22%5BTitle%2FAbstract%5D%29%29+OR+%28%22Neurological+Physiotherapy%22%5BTitle%2FAbstract%5D%29%29+OR+%28%22Physiotherapy%2C+Neurological%22%5BTitle%2FAbstract%5D%29%29+OR+%28%22Neurophysiotherapy%22%5BTitle%2FAbstract%5D%29&sort=) | 22:29:42 |
| #9 | Search: “Physical Therapy Modalities”[Mesh] Sort by: Most Recent | [178,918](https://pubmed.ncbi.nlm.nih.gov/?sort=date&term=%22Physical+Therapy+Modalities%22%5BMesh%5D) | 22:26:13 |
| #8 | Search: ((((((((((“Remedial Exercise”[Title/Abstract]) OR (“Exercise, Remedial”[Title/Abstract])) OR (“Exercise, Remedial”[Title/Abstract])) OR (“Exercise, Remedial”[Title/Abstract])) OR (“Therapy, Exercise”[Title/Abstract])) OR (“Therapy, Exercise”[Title/Abstract])) OR (“Therapy, Exercise”[Title/Abstract])) OR (“Rehabilitation Exercise”[Title/Abstract])) OR (“Rehabilitation Exercise”[Title/Abstract])) OR (“Exercises, Rehabilitation”[Title/Abstract])) OR (“Rehabilitation Exercises”[Title/Abstract]) | [2081](https://pubmed.ncbi.nlm.nih.gov/?term=%28%28%28%28%28%28%28%28%28%28%22Remedial+Exercise%22%5BTitle%2FAbstract%5D%29+OR+%28%22Exercise%2C+Remedial%22%5BTitle%2FAbstract%5D%29%29+OR+%28%22Exercise%2C+Remedial%22%5BTitle%2FAbstract%5D%29%29+OR+%28%22Exercise%2C+Remedial%22%5BTitle%2FAbstract%5D%29%29+OR+%28%22Therapy%2C+Exercise%22%5BTitle%2FAbstract%5D%29%29+OR+%28%22Therapy%2C+Exercise%22%5BTitle%2FAbstract%5D%29%29+OR+%28%22Therapy%2C+Exercise%22%5BTitle%2FAbstract%5D%29%29+OR+%28%22Rehabilitation+Exercise%22%5BTitle%2FAbstract%5D%29%29+OR+%28%22Rehabilitation+Exercise%22%5BTitle%2FAbstract%5D%29%29+OR+%28%22Exercises%2C+Rehabilitation%22%5BTitle%2FAbstract%5D%29%29+OR+%28%22Rehabilitation+Exercises%22%5BTitle%2FAbstract%5D%29&sort=) | 22:24:04 |
| #7 | Search: ((((((((((((((((((((((((“Exercises”[Title/Abstract]) OR (“Physical Activity”[Title/Abstract])) OR (“Activities, Physical”[Title/Abstract])) OR (“Activity, Physical”[Title/Abstract])) OR (“Physical Activities”[Title/Abstract])) OR (“Exercise, Physical”[Title/Abstract])) OR (“Exercises, Physical”[Title/Abstract])) OR (“Physical Exercise”[Title/Abstract])) OR (“Physical Exercises”[Title/Abstract])) OR (“Acute Exercise”[Title/Abstract])) OR (“Acute Exercises”[Title/Abstract])) OR (“Exercise, Acute”[Title/Abstract])) OR (“Exercises, Acute”[Title/Abstract])) OR (“Exercise, Isometric”[Title/Abstract])) OR (“Exercises, Isometric”[Title/Abstract])) OR (“Isometric Exercises”[Title/Abstract])) OR (“Isometric Exercise”[Title/Abstract])) OR (“Exercise, Aerobic”[Title/Abstract])) OR (“Aerobic Exercise”[Title/Abstract])) OR (“Aerobic Exercises”[Title/Abstract])) OR (“Exercises, Aerobic”[Title/Abstract])) OR (“Exercise Training”[Title/Abstract])) OR (“Exercise Trainings”[Title/Abstract])) OR (“Training, Exercise”[Title/Abstract])) OR (“Trainings, Exercise”[Title/Abstract]) | [242,726](https://pubmed.ncbi.nlm.nih.gov/?term=%28%28%28%28%28%28%28%28%28%28%28%28%28%28%28%28%28%28%28%28%28%28%28%28%22Exercises%22%5BTitle%2FAbstract%5D%29+OR+%28%22Physical+Activity%22%5BTitle%2FAbstract%5D%29%29+OR+%28%22Activities%2C+Physical%22%5BTitle%2FAbstract%5D%29%29+OR+%28%22Activity%2C+Physical%22%5BTitle%2FAbstract%5D%29%29+OR+%28%22Physical+Activities%22%5BTitle%2FAbstract%5D%29%29+OR+%28%22Exercise%2C+Physical%22%5BTitle%2FAbstract%5D%29%29+OR+%28%22Exercises%2C+Physical%22%5BTitle%2FAbstract%5D%29%29+OR+%28%22Physical+Exercise%22%5BTitle%2FAbstract%5D%29%29+OR+%28%22Physical+Exercises%22%5BTitle%2FAbstract%5D%29%29+OR+%28%22Acute+Exercise%22%5BTitle%2FAbstract%5D%29%29+OR+%28%22Acute+Exercises%22%5BTitle%2FAbstract%5D%29%29+OR+%28%22Exercise%2C+Acute%22%5BTitle%2FAbstract%5D%29%29+OR+%28%22Exercises%2C+Acute%22%5BTitle%2FAbstract%5D%29%29+OR+%28%22Exercise%2C+Isometric%22%5BTitle%2FAbstract%5D%29%29+OR+%28%22Exercises%2C+Isometric%22%5BTitle%2FAbstract%5D%29%29+OR+%28%22Isometric+Exercises%22%5BTitle%2FAbstract%5D%29%29+OR+%28%22Isometric+Exercise%22%5BTitle%2FAbstract%5D%29%29+OR+%28%22Exercise%2C+Aerobic%22%5BTitle%2FAbstract%5D%29%29+OR+%28%22Aerobic+Exercise%22%5BTitle%2FAbstract%5D%29%29+OR+%28%22Aerobic+Exercises%22%5BTitle%2FAbstract%5D%29%29+OR+%28%22Exercises%2C+Aerobic%22%5BTitle%2FAbstract%5D%29%29+OR+%28%22Exercise+Training%22%5BTitle%2FAbstract%5D%29%29+OR+%28%22Exercise+Trainings%22%5BTitle%2FAbstract%5D%29%29+OR+%28%22Training%2C+Exercise%22%5BTitle%2FAbstract%5D%29%29+OR+%28%22Trainings%2C+Exercise%22%5BTitle%2FAbstract%5D%29&sort=) | 22:21:00 |
| #6 | Search: “Exercise Therapy”[Mesh] OR “Exercise”[Mesh] Sort by: Most Recent | [283,816](https://pubmed.ncbi.nlm.nih.gov/?sort=date&term=%22Exercise+Therapy%22%5BMesh%5D+OR++%22Exercise%22%5BMesh%5D) | 22:16:37 |
| #5 | Search: (“Self Management”[Title/Abstract]) OR (“Management, Self”[Title/Abstract]) | [27,371](https://pubmed.ncbi.nlm.nih.gov/?term=%28%22Self+Management%22%5BTitle%2FAbstract%5D%29+OR+%28%22Management%2C+Self%22%5BTitle%2FAbstract%5D%29&sort=) | 22:14:08 |
| #4 | Search: “Self-Management”[Mesh] Sort by: Most Recent | [5383](https://pubmed.ncbi.nlm.nih.gov/?sort=date&term=%22Self-Management%22%5BMesh%5D) | 22:13:13 |
| #3 | Search: #1 or #2 | [31,507](https://pubmed.ncbi.nlm.nih.gov/?term=%231+or+%232&sort=) | 22:12:12 |
| #2 | Search: (((((((((((((((((((((((“Disorder, Temporomandibular Joint”[Title/Abstract]) OR (“Disorders, Temporomandibular Joint”[Title/Abstract])) OR (“Joint Disorder, Temporomandibular”[Title/Abstract])) OR (“Joint Disorders, Temporomandibular”[Title/Abstract])) OR (“Temporomandibular Joint Disorder”[Title/Abstract])) OR (“TMJ Disorders or Disorder, TMJ”[Title/Abstract])) OR (“Disorders, TMJ”[Title/Abstract])) OR (“TMJ Disorder”[Title/Abstract])) OR (“Temporomandibular Disorders”[Title/Abstract])) OR (“Disorder, Temporomandibular”[Title/Abstract])) OR (“Disorders, Temporomandibular”[Title/Abstract])) OR (“Temporomandibular Disorder”[Title/Abstract])) OR (“Temporomandibular Joint Diseases”[Title/Abstract])) OR (“Disease, Temporomandibular Joint”[Title/Abstract])) OR (“Diseases, Temporomandibular Joint”[Title/Abstract])) OR (“Joint Disease, Temporomandibular”[Title/Abstract])) OR (“Joint Diseases, Temporomandibular”[Title/Abstract])) OR (“Temporomandibular Joint Disease”[Title/Abstract])) OR (“TMJ Diseases”[Title/Abstract])) OR (“Disease, TMJ”[Title/Abstract])) OR (“Diseases, TMJ”[Title/Abstract])) OR (“TMJ Disease”[Title/Abstract])) OR “Facial Pain”[MeSH Terms] OR “Face Pain”[Title/Abstract] OR “Face Pain”[Title/Abstract] OR “Orofacial Pain”[Title/Abstract] OR “Orofacial Pain”[Title/Abstract] OR “Neuralgic Facial Pain”[Title/Abstract] OR “Craniofacial Pain”[Title/Abstract] OR “pain craniofacial”[Title/Abstract] OR “Myofacial Pain”[Title/Abstract] | 16,833 | 22:11:50 |
| #1 | Search: “Temporomandibular Joint Disorders”[Mesh] Sort by: Most Recent | [18,939](https://pubmed.ncbi.nlm.nih.gov/?sort=date&term=%22Temporomandibular+Joint+Disorders%22%5BMesh%5D) | 22:07:55 |

Database: Web of science (16 August 2023)

1: ((((((((((((((((((((((((TS=(“Temporomandibular Joint Disorders”)) OR TS=(“Disorder, Temporomandibular Joint”)) OR TS=(“Disorders, Temporomandibular Joint”)) OR TS=(“Joint Disorder, Temporomandibular”)) OR TS=(“Joint Disorders, Temporomandibular”)) OR TS=(“Temporomandibular Joint Disorder”)) OR TS=(“TMJ Disorders or Disorder, TMJ”)) OR TS=(“Disorders, TMJ”)) OR TS=(“TMJ Disorder”)) OR TS=(“Temporomandibular Disorders”)) OR TS=(“Disorder, Temporomandibular”)) OR TS=(“Disorders, Temporomandibular”)) OR TS=(“Temporomandibular Disorder”)) OR TS=(“Temporomandibular Joint Diseases”)) OR TS=(“Disease, Temporomandibular Joint”)) OR TS=(“Diseases, Temporomandibular Joint”)) OR TS=(“Joint Disease, Temporomandibular”)) OR TS=(“Joint Diseases, Temporomandibular”)) OR TS=(“Temporomandibular Joint Disease”)) OR TS=(“TMJ Diseases”)) OR TS=(“Disease, TMJ”)) OR TS=(“Diseases, TMJ”)) OR TS=(“TMJ Disease”)) OR TS=(“facial pain”)) OR TS=(“orofacial pain”) OR TS=(“Pain, Facial”) OR TS=(“Pain, Face”) OR TS=(“Pain, Orofacial”) OR TS=(“Craniofacial Pain”) OR TS=(“Pain, Craniofacial”) OR TS=(“Craniofacial Pain”) OR TS=(“Myofacial Pain”) OR TS=(“Pain, Myofacial”) 14,772

2: ((((((((((((((((((((((((((((((((((((((TS=(“Self-Management”)) OR TS=(“Management, Self”)) OR TS=(“Exercise Therapy”)) OR TS=(“Exercise”)) OR TS=(“Physical Activity”)) OR TS=(“Activities, Physical”)) OR TS=(“Activity, Physical”)) OR TS=(“Physical Activities”)) OR TS=(“Exercise, Physical”)) OR TS=(“Exercises, Physical”)) OR TS=(“Physical Exercise”)) OR TS=(“Physical Exercises”)) OR TS=(“Acute Exercise”)) OR TS=(“Acute Exercises”)) OR TS=(“Exercise, Acute”)) OR TS=(“Exercises, Acute”)) OR TS=(“Exercise, Isometric”)) OR TS=(“Exercises, Isometric”)) OR TS=(“Isometric Exercises”)) OR TS=(“Isometric Exercise”)) OR TS=(“Exercise, Aerobic”)) OR TS=(“Aerobic Exercise”)) OR TS=(“Aerobic Exercises”)) OR TS=(“Exercises, Aerobic”)) OR TS=(“Exercise Training”)) OR TS=(“Exercise Trainings”)) OR TS=(“Training, Exercise”)) OR TS=(“Trainings, Exercise”)) OR TS=(“Remedial Exercise”)) OR TS=(“Exercise, Remedial”)) OR TS=(“Exercise, Remedial”)) OR TS=(“Exercise, Remedial”)) OR TS=(“Therapy, Exercise”)) OR TS=(“Therapy, Exercise”)) OR TS=(“Therapy, Exercise”)) OR TS=(“Rehabilitation Exercise”)) OR TS=(“Rehabilitation Exercise”)) OR TS=(“Exercises, Rehabilitation”)) OR TS=(“Rehabilitation Exercises”) 722,851

3: #1 AND #2 482

4: Excluded review paper 398

Database: Embase (16 August 2023)

#6: #5 AND (“clinical trial”/de OR “randomized controlled trial”/de) 151

#5: #4 AND #3 889

#4: “exercises” OR “physical activity”/exp OR “physical activity” OR “activities, physical”:ab,ti OR “activity, physical”:ab,ti OR “physical activities”:ab,ti OR “exercise, physical”:ab,ti OR “exercises, physical”:ab,ti OR “physical exercise”:ab,ti OR “physical exercises”:ab,ti OR “acute exercise”:ab,ti OR “acute exercises”:ab,ti OR “exercise, acute”:ab,ti OR “exercises, acute”:ab,ti OR “exercise, isometric”:ab,ti OR “exercises, isometric”:ab,ti OR “isometric exercises”:ab,ti OR “isometric exercise”:ab,ti OR “exercise, aerobic”:ab,ti OR “aerobic exercise”:ab,ti OR “aerobic exercises”:ab,ti OR “exercises, aerobic”:ab,ti OR “exercise training”:ab,ti OR “exercise trainings”:ab,ti OR “training, exercise”:ab,ti OR “trainings, exercise”:ab,ti OR “self-management”:ab,ti OR “management, self”:ab,ti OR “remedial exercise”:ab,ti OR “exercise, remedial”:ab,ti OR “therapy, exercise”:ab,ti OR “rehabilitation exercise”:ab,ti OR “exercises, rehabilitation”:ab,ti OR “rehabilitation exercises”:ab,ti OR “modalities, physical therapy”:ab,ti OR “modality, physical therapy”:ab,ti OR “physical therapy modality”:ab,ti OR (physiotherapy:ab,ti AND techniques:ab,ti) OR (physiotherapies:ab,ti AND techniques:ab,ti) OR “home health care nursing”:ab,ti OR “nursing, home health”:ab,ti OR “physical therapy techniques”:ab,ti OR “physical therapy technique”:ab,ti OR “techniques, physical therapy”:ab,ti OR “group physiotherapy”:ab,ti OR “group physiotherapies”:ab,ti OR “physiotherapies, group”:ab,ti OR “physiotherapy, group”:ab,ti OR “physical therapy”:ab,ti OR “physical therapies”:ab,ti OR “manipulations, musculoskeletal”:ab,ti OR “therapy, physical”:ab,ti OR “manipulation therapy”:ab,ti OR “manipulative therapies”:ab,ti OR “therapies, manipulative”:ab,ti OR “manipulative therapy”:ab,ti OR “therapy, manipulation”:ab,ti OR “therapy, manipulative”:ab,ti OR “therapies, manipulation”:ab,ti OR “manipulation therapies”:ab,ti OR “manual therapies”:ab,ti OR “massage, craniosacral”:ab,ti OR “craniosacral massage”:ab,ti OR “manual therapy”:ab,ti OR “therapies, manual”:ab,ti OR “therapy, manual”:ab,ti 785,309

#3: #1 OR #2 [26,984](https://www.embase.com/)

#2: “disorder, temporomandibular joint”:ab,ti OR “disorders, temporomandibular joint”:ab,ti OR “joint disorder, temporomandibular”:ab,ti OR “joint disorders, temporomandibular”:ab,ti OR “temporomandibular joint disorder”:ab,ti OR “tmj disorders”:ab,ti OR “disorder, tmj”:ab,ti OR “disorders, tmj”:ab,ti OR “tmj disorder”:ab,ti OR “temporomandibular disorders”:ab,ti OR “disorder, temporomandibular”:ab,ti OR “disorders, temporomandibular”:ab,ti OR “temporomandibular disorder”:ab,ti OR “temporomandibular joint diseases”:ab,ti OR “disease, temporomandibular joint”:ab,ti OR “diseases, temporomandibular joint”:ab,ti OR “joint disease, temporomandibular”:ab,ti OR “joint diseases, temporomandibular”:ab,ti OR “temporomandibular joint disease”:ab,ti OR “tmj diseases”:ab,ti OR “disease, tmj”:ab,ti OR “diseases, tmj”:ab,ti OR “tmj disease”:ab,ti OR “facial pain”:ab,ti OR “face pain”:ab,ti OR “orofacial pain”:ab,ti OR “craniofacial pain”:ab,ti OR “pain craniofacial”:ab,ti OR “myofacial pain”:ab,ti [16,089](https://www.embase.com/)

#1: “temporomandibular joint disorder”/exp OR “temporomandibular joint disorder” 17,257

Database: Cochrane (16 August 2023)

#1 MeSH descriptor: [Temporomandibular Joint Disorders] explode all trees 901

#2 (“Disorder, Temporomandibular Joint” or “Disorders, Temporomandibular Joint” or “Joint Disorder, Temporomandibular” or “Joint Disorders, Temporomandibular” or “Temporomandibular Joint Disorder” or “TMJ Disorders or Disorder, TMJ” or “Disorders, TMJ” or “TMJ Disorder” or “Temporomandibular Disorders” or “Disorder, Temporomandibular” or “Disorders, Temporomandibular” or “Temporomandibular Disorder” or “Temporomandibular Joint Diseases” or “Disease, Temporomandibular Joint” or “Diseases, Temporomandibular Joint” or “Joint Disease, Temporomandibular” or “Joint Diseases, Temporomandibular” or “Temporomandibular Joint Disease” or “TMJ Diseases” or “Disease, TMJ” or “Diseases, TMJ” or “TMJ Disease” or TMD or “facial pain” or “orofacial pain”):ti,ab,kw (Word variations have been searched) 2695

#3 #1 or #2 2780

#4 MeSH descriptor: [Exercise] explode all trees 28,782

#5 MeSH descriptor: [Rehabilitation] explode all trees 41,269

#6 MeSH descriptor: [Physical Therapy Modalities] explode all trees 29,938

#7 MeSH descriptor: [Musculoskeletal Manipulations] explode all trees 3374

#8 MeSH descriptor: [Home Health Nursing] explode all trees 9

#9 MeSH descriptor: [Self-Management] explode all trees 725

#10 (“Exercises” or “Physical Activity” or “Activities, Physical” or “Activity, Physical” or “Physical Activities” or “Exercise, Physical” or “Exercises, Physical” or “Physical Exercise” or “Physical Exercises” or “Acute Exercise” or “Acute Exercises” or “Exercise, Acute” or “Exercises, Acute” or “Exercise, Isometric” or “Exercises, Isometric” or “Isometric Exercises” or “Isometric Exercise” or “Exercise, Aerobic” or “Aerobic Exercise” or “Aerobic Exercises” or “Exercises, Aerobic” or “Exercise Training” or “Exercise Trainings” or “Training, Exercise” or “Trainings, Exercise”):ti,ab,kw (Word variations have been searched) 143,671

#11 (“Modalities, Physical Therapy” or “Modality, Physical Therapy” or “Physical Therapy Modality” or “Physiotherapy (Techniques)” or “Physiotherapies (Techniques)” or “Physical Therapy Techniques” or “Physical Therapy Technique” or “Techniques, Physical Therapy” or “Group Physiotherapy” or “Group Physiotherapies” or “Physiotherapies, Group” or “Physiotherapy, Group” or “Physical Therapy” or “Physical Therapies” or “Therapy, Physical” or “Neurological Physiotherapy” or “Physiotherapy, Neurological” or “Neurophysiotherapy”):ti,ab,kw (Word variations have been searched) 12,885

#12 (“Nursing, Home Health” or “Home Health Care Nursing”):ti,ab,kw (Word variations have been searched) 24

#13 (“Manipulations, Musculoskeletal” or “Manipulation Therapy” or “Manipulative Therapies” or “Manipulative Therapy” or “Therapies, Manipulative” or “Therapy, Manipulative” or “Therapy, Manipulation” or “Manipulation Therapies” or “Therapies, Manipulation” or “Reflexology” or “Bodywork” or “Bodyworks” or “Rolfing” or “Craniosacral Massage” or “Massage, Craniosacral” or “Manual Therapies” or “Manual Therapy” or “Therapies, Manual” or “Therapy, Manual”):ti,ab,kw (Word variations have been searched) 3367

#14 (“Self Management” or “Management, Self”):ti,ab,kw (Word variations have been searched) 10037

#15 #4 or #5 or #6 or #7 or #8 or #9 or #10 or #11 or #12 or #13 or #14 179,870

#16 #3 and #15 and “randomized controlled trial” 152

Database: ClinicalTrials. gov (16 August 2023)

Exercise OR Rehabilitation OR “Physical Therapy Modalities” OR “Musculoskeletal Manipulations” OR “Home Health Nursing” OR “Self-Management” | “Temporomandibular Joint Disorders” AND “[randomized controlled trial](javascript:;)” AND 5
