# Supplementary material for: Home-based rehabilitation versus centre-based programs in patients with temporomandibular disorders—a systematic review and meta-analysis
Source: J Oral Facial Pain Headache. 2024 Mar 12;38(1):1–16. doi: 10.22514/jofph.2024.002 (PMC11798648; doi:10.22514/jofph.2024.002)
Supplement: Supplementary file 2 [file Supplementary-material-2.docx]

Supplementary material

Supplementary Table 1. A short question guide for the selection of relevant studies based on inclusion criteria.

| Item | Question | Action |
| --- | --- | --- |
| 1 | Did the study use any of the eligible study design? | Yes, move to the next question  No, exclude |
| 2 | Did the study involve people with temporomandibular joint disorders (TMD)? | Yes, move to the next question  No, exclude |
| 3 | Did the study involve people with TMD after trauma, pre-post surgery or fracture? | Yes, exclude  No, move to the next question |
| 4 | Whether the home-based rehabilitation in the study meets the criteria? | Yes, move to the next question  No, exclude |
| 5 | Did the study compare home-based rehabilitation with other treatment modalities (some techniques need a trained individual)? | Yes, move to the next question  No, exclude |

Supplementary Table 2. After reading the full text, the study was excluded and the reasons for exclusion.

| Exclusion criteria and excluded articles (n = 81) | |
| --- | --- |
| Non-randomized controlled trial  (n = 15) | Greene *et al.* [1]  Nickerson *et al.* [2]  McCarty *et al.* [3]  Dworkin *et al.* [4]  Nicolakis *et al.* [5]  Michelotti *et al.* [6]  Furto *et al.* [7]  Almeida *et al.* [8]  Navratil *et al.* [9]  Oh, J. S. *et al.* [10]  Merrill *et al.* [11]  Henien *et al.* [12]  Enache *et al.* [13]  Liu *et al.* [14]  Raustia *et al.* [15] |
| Non- home-based rehabilitation compared with other center-based rehabilitation  (n = 45) | Crockett *et al.* [16]  Raustia *et al.* [17]  List *et al.* [18]  Dworkin *et al.* [19]  Stegenga *et al.* [20]  Petersson *et al.* [21]  Wright *et al.* [22]  Stiesch *et al.* [23]  Ismail *et al.* [24]  Bakke *et al.* [25]  Diraçoğlu *et al.* [26]  La Touche, R *et al.* [27]  Cuccia *et al.* [28]  de Felício *et al.* [29]  Maluf *et al.* [30]  Madani *et al.* [31]  Turner *et al.* [32]  Ferrando *et al.* [33]  Amaral *et al.* [34]  El Hage *et al.* [35]  Vos *et al.* [36]  da Costa *et al.* [37]  De Giorgi *et al.* [38]  Aksu *et al.* [39]  Oliveira *et al.* [40]  Espí-López *et al.* [41]  Kanungo *et al.* [42]  Ashraf *et al.* [43]  Magesty *et al.* [44]  Pundkar *et al.* [45]  Sahin *et al.* [46]  Pihut *et al.* [47]  Tanhan *et al.* [48]  Kalamir *et al.* [49]  Thiruvevenkadam *et al.* [50]  Tang *et al.* [51]  Michelotti *et al.* [52]  Sarfraz *et al.* [53]  Ke, X. Q *et al.* [54]  Minakuchi *et al.* [55]  Schiffman *et al.* [56]  Kraaijenga *et al.* [57]  Nagata *et al.* [58]  Daruri *et al.* [59]  Brandao *et al.* [60] |
| Conference papers, poster only  (n = 3) | Lucas *et al.* [61]  Brignardello *et al.* [62]  Haketa *et al.* [63] |
| Study protocol (no results have been published yet)  (n = 3) | Kim *et al.* [64]  Incorvati *et al.* [65]  Kim *et al.* [66] |
| Subjects were not patients with TMJ disorders, such as traumatic or odontogenic pain  (n = 4) | Roth *et al.* [67]  Geisser *et al.* [68]  Galindez *et al.* [69]  Klobas *et al.* [70] |
| Full text unavailable  (n = 2) | Doerfler *et al.* [71]  Magnusson *et al.* [72] |
| No suitable result data is available  (n = 5) | Kalamir *et al.* [73]  Tavera *et al.* [74]  Kokkola *et al.* [75]  Zwiri *et al.* [76]  Lindfors *et al.* [77] |
| Duplicate article  (n = 4) | de la Serna *et al.* [78]  Coskun *et al.* [79]  Maluf *et al.* [80]  Olbort *et al.* [81] |

References

[1] Greene CS, Laskin DM. Long-term evaluation of conservative treatment for myofascial pain-dysfunction syndrome. The Journal of the American Dental Association. 1974; 89: 1365–1368.

[2] Nickerson JW. Exercising judgment in the treatment of temporomandibular disorders. Journal of Oral and Maxillofacial Surgery. 1988; 46: 444, 519.

[3] McCarty WL, Darnell MW. Rehabilitation of the temporomandibular joint through the application of motion. CRANIO®. 1993; 11: 298–307.

[4] Dworkin SF. Behavioral and educational modalities. Oral Surgery, Oral Medicine, Oral Pathology, Oral Radiology, and Endodontology. 1997; 83: 128–133.

[5] Nicolakis P, Erdogmus B, Kopf A, Nicolakis M, Piehslinger E, Fialka-Moser V. Effectiveness of exercise therapy in patients with myofascial pain dysfunction syndrome. Journal of Oral Rehabilitation. 2002; 29: 362–368.

[6] Michelotti A, De Wijer A, Steenks M, Farella M. Home-exercise regimes for the management of non‐specific temporomandibular disorders. Journal of Oral Rehabilitation. 2005; 32: 779–785.

[7] Furto ES, Cleland JA, Whitman JM, Olson KA. Manual physical therapy interventions and exercise for patients with temporomandibular disorders. CRANIO®. 2006; 24: 283–291.

[8] Almeida AF, Berni KC, Rodrigues-Bigaton D. Effect of treatment with HVES on pain and electromyography activity in patients with TMD. Electromyogr Clin Neurophysiol. 2009; 49: 245–254

[9] Navrátil L, Navratil V, Hajkova S, Hlinakova P, Dostalova T, Vranová J. Comprehensive treatment of temporomandibular joint disorders. CRANIO®. 2014; 32: 24–30.

[10] Oh J, Kim S, Park K. Effects of controlled condylar rotation exercise on symmetrical mouth opening in patients with temporomandibular disorder. Journal of Physical Therapy Science. 2015; 27: 1319–1321.

[11] Merrill RL, Goodman D. Chronic orofacial pain and behavioral medicine. Oral and Maxillofacial Surgery Clinics of North America. 2016; 28: 247–260.

[12] Henien M, Sproat C. Interactive group therapy for the management of myofascial temporomandibular pain. British Dental Journal. 2017; 223: 90–95.

[13] Enache AM, Becheanu AG, Festila D. Conservative treatment of disc displacement without reduction—case report. Romanian Journal of Oral Rehabilitation. 2020; 12: 144–151.

[14] Liu SS, Fan S, Li GP, Cai B, Yao Y, Jin L, *et al*. Short term effects of a novel combined approach compared with physical therapy alone among older patients with temporomandibular degenerative joint disease: a prospective cohort study. BMC Oral Health. 2023; 23: 173.

[15] Raustia AM. Diagnosis and treatment of temporomandibular joint dysfunction. Results of a computed tomography study of the temporomandibular joint and physiological masticatory treatment compared to acupuncture. Proceedings of the Finnish Dental Society. 1987; 83: 205–207. (In Finnish)

[16] Crockett DJ, Foreman ME, Alden L, Blasberg B. A comparison of treatment modes in the management of myofascial pain dysfunction syndrome. Biofeedback and Self-Regulation. 1986; 11: 279–291.

[17] Raustia AM, Pohjola RT. Acupuncture compared with stomatognathic treatment for TMJ dysfunction. Part III: effect of treatment on mobility. The Journal of Prosthetic Dentistry. 1986; 56: 616–623.

[18] List T, Helkimo M, Andersson S, Carlsson GE. Acupuncture and occlusal splint therapy in the treatment of craniomandibular disorders. Part I. A comparative study. Swedish Dental Journal. 1992; 16: 125–141.

[19] Dworkin SF, Huggins KH, Wilson L, Mancl L, Turner J, Massoth D, *et al*. A randomized clinical trial using research diagnostic criteria for temporomandibular disorders-axis II to target clinic cases for a tailored self-care TMD treatment program. Journal of Orofacial Pain. 2002; 16: 48–63.

[20] Stegenga B, de Bont LGM, Dijkstra PU, Boering G. Short-term outcome of arthroscopic surgery of temporomandibular joint osteoarthrosis and internal derangement: a randomized controlled clinical trial. British Journal of Oral and Maxillofacial Surgery. 1993; 31: 3–14.

[21] Petersson A, Eriksson L, Lundh H. No short-term difference in outcome after temporomandibular joint arthrography alone or with immediate lavage. Oral Surgery, Oral Medicine, Oral Pathology. 1994; 77: 322–326.

[22] Wright EF, Domenech MA, Fischer JR. Usefulness of posture training for patients with temporomandibular disorders. The Journal of the American Dental Association. 2000; 131: 202–210.

[23] Stiesch-Scholz M, Fink M, Tschernitschek H, Roßbach A. Medical and physical therapy of temporomandibular joint disk displacement without reduction. CRANIO®. 2002; 20: 85–90.

[24] Ismail F, Demling A, Heßling K, Fink M, Stiesch‐Scholz M. Short‐term efficacy of physical therapy compared to splint therapy in treatment of arthrogenous TMD. Journal of Oral Rehabilitation. 2007; 34: 807–813.

[25] Bakke M, Eriksson L, Thorsen NM, Sewerin I, Petersson A, Wagner A. Modified condylotomy versus conventional conservative treatment in painful reciprocal clicking—a preliminary prospective study in eight patients. Clinical Oral Investigations. 2008; 12: 353–359.

[26] Dıraçoğlu D, Saral IB, Keklik B, Kurt H, Emekli U, Özçakar L, *et al*. Arthrocentesis versus nonsurgical methods in the treatment of temporomandibular disc displacement without reduction. Oral Surgery, Oral Medicine, Oral Pathology, Oral Radiology, and Endodontology. 2009; 108: 3–8.

[27] La Touche R, Fernández-De-Las-Peñas C, Fernández-Carnero J, Escalante K, Angulo-Díaz-Parreño S, Paris-Alemany A, *et al*. The effects of manual therapy and exercise directed at the cervical spine on pain and pressure pain sensitivity in patients with myofascial temporomandibular disorders. Journal of Oral Rehabilitation. 2009; 36: 644–652.

[28] Cuccia AM, Caradonna C, Annunziata V, Caradonna D. Osteopathic manual therapy versus conventional conservative therapy in the treatment of temporomandibular disorders: a randomized controlled trial. Journal of Bodywork and Movement Therapies. 2010; 14: 179–184.

[29] de Felício CM, Melchior MDO, da Silva MAMR. Effects of orofacial myofunctional therapy on temporomandibular disorders. CRANIO®. 2010; 28: 249–259.

[30] Maluf SA, Moreno BGD, Crivello O, Cabral CMN, Bortolotti G, Marques AP. Global postural reeducation and static stretching exercises in the treatment of myogenic temporomandibular disorders: a randomized study. Journal of Manipulative and Physiological Therapeutics. 2010; 33: 500–507.

[31] Madani AS, Mirmortazavi A. Comparison of three treatment options for painful temporomandibular joint clicking. Journal of Oral Science. 2011; 53: 349–354.

[32] Turner JA, Mancl L, Huggins KH, Sherman JJ, Lentz G, LeResche L. Targeting temporomandibular disorder pain treatment to hormonal fluctuations: a randomized clinical trial. Pain. 2011; 152: 2074–2084.

[33] Ferrando M, Galdón MJ, Durá E, Andreu Y, Jiménez Y, Poveda R. Enhancing the efficacy of treatment for temporomandibular patients with muscular diagnosis through cognitive-behavioral intervention, including hypnosis: a randomized study. Oral Surgery, Oral Medicine, Oral Pathology and Oral Radiology. 2012; 113: 81–89.

[34] Amaral AP, Politti F, Hage YE, Arruda EE, Amorin CF, Biasotto-Gonzalez DA. Immediate effect of nonspecific mandibular mobilization on postural control in subjects with temporomandibular disorder: a single-blind, randomized, controlled clinical trial. Brazilian Journal of Physical Therapy. 2013; 17: 121–127.

[35] El Hage Y, Politti F, de Sousa D, Herpich C, Gloria I, Gomes C, *et al*. Effect of mandibular mobilization on electromyographic signals in muscles of mastication and static balance in individuals with temporomandibular disorder: study protocol for a randomized controlled trial. Trials. 2013; 14: 316.

[36] Vos LM, Huddleston Slater JJR, Stegenga B. Arthrocentesis as initial treatment for temporomandibular joint arthropathy: a randomized controlled trial. Journal of Cranio-Maxillofacial Surgery. 2014; 42: e134–e139.

[37] da Costa LMR, Schimit EFD, Souza C, Wagner Neto ES, de Souza da Silva L, Candotti CT, *et al*. Effect of the Pilates method on women with temporomandibular disorders: a study protocol for a randomized controlled trial. Journal of Bodywork and Movement Therapies. 2016; 20: 110–114.

[38] De Giorgi I, Castroflorio T, Sartoris B, Deregibus A. The use of conventional transcutaneous electrical nerve stimulation in chronic facial myalgia patients. Clinical Oral Investigations. 2017; 21: 275–280.

[39] Aksu Ö, Pekin Doğan Y, Sayıner Çağlar N, Şener BM. Comparison of the efficacy of dry needling and trigger point injections with exercise in temporomandibular myofascial pain treatment. Turkish Journal of Physical Medicine and Rehabilitation. 2019; 65: 228–235.

[40] Oliveira SSI, Pannuti CM, Paranhos KS, Tanganeli JPC, Laganá DC, Sesma N, *et al*. Effect of occlusal splint and therapeutic exercises on postural balance of patients with signs and symptoms of temporomandibular disorder. Clinical and Experimental Dental Research. 2019; 5: 109–115.

[41] Espí-López GV, Arnal-Gómez A, Del Pino AC, Benavent-Corai J, Serra-Añó P, Inglés M. Effect of manual therapy and splint therapy in people with temporomandibular disorders: a preliminary study. Journal of Clinical Medicine. 2020; 9: 1–15.

[42] Kanungo B, Patra RC, Mohanty P, Bawa P. Physical therapy approach in conjunction with dry needling on health related quality of life in patients with temporomandibular disorder: a randomized control trial. Indian Journal of Public Health Research and Development. 2020; 11: 187–192.

[43] Ashraf N, Aneeq Ur R, Mustansar A, Noor S, Latif W, Laique T. Immediate and prolonged effects of temporomandibular joint mobilization on pain, range of motion in hypo mobile temporo-mandibular joint. Pakistan Journal of Medical & Health Sciences. 2021; 15: 1124–1127.

[44] Magesty RA, da Silva MAM, Simões CASC, Falci SGM, Douglas-de-Oliveira DW, Gonçalves PF, *et al*. Oral health-related quality of life in patients with disc displacement with reduction after counselling treatment versus counselling associated with jaw exercises. Journal of Oral Rehabilitation. 2021; 48: 369–374.

[45] Pundkar S, Patil D, Naqvi W. A comparative study on effectiveness of rocabado approach and conventional physiotherapy on pain, rom and QOL in patients with TMJ dysfunction. Journal of Pharmaceutical Research International. 2021; 33: 201–209.

[46] Şahin D, Kaya Mutlu E, Şakar O, Ateş G, İnan Ş, Taşkıran H. The effect of the ischaemic compression technique on pain and functionality in temporomandibular disorders: a randomised clinical trial. Journal of Oral Rehabilitation. 2021; 48: 531–541.

[47] Pihut M, Zarzecka-Francica E, Gala A. Physiotherapeutic rehabilitation of adolescent patients with temporomandibular disorders. Folia Medica Cracoviensia. 2022; 62: 79–90.

[48] Tanhan A, Ozer AY, Polat MG. Efficacy of different combinations of physiotherapy techniques compared to exercise and patient education in temporomandibular disorders: a randomized controlled study. CRANIO®. 2023; 41: 389–401.

[49] Kalamir A, Pollard H, Vitiello A, Bonello R. Intra-oral myofascial therapy for chronic myogenous temporomandibular disorders: a randomized, controlled pilot study. Journal of Manual & Manipulative Therapy. 2010; 18: 139–146.

[50] Thiruvevenkadam IA, Ling LT. Effect of cervical extensor strengthening on severity of temporomandibular joint disorder among university students: a randomized controlled trial. Research Journal of Pharmacy and Technology. 2021; 14: 2233–2242.

[51] Tang YH, Vos LM, Tuin AJ, Huddleston Slater JJR, Gareb B, van Bakelen NB, *et al*. Arthrocentesis versus non-surgical intervention as initial treatment for temporomandibular joint arthralgia: a randomized controlled trial with long-term follow-up. International Journal of Oral and Maxillofacial Surgery. 2023; 52: 595–603.

[52] Michelotti A, Steenks MH, Farella M, Parisini F, Cimino R, Martina R. The additional value of a home physical therapy regimen versus patient education only for the treatment of myofascial pain of the jaw muscles: short-term results of a randomized clinical trial. Journal of Orofacial Pain. 2004; 18: 114–125.

[53] Sarfraz S, Anwar N, Tauqeer S, Asif T, Ain NU, Shakeel H. Comparison of effects of manual physical therapy and exercise therapy for patients with Temporomandibular disorders. Journal of The Pakistan Medical Association. 2023; 73: 129–130.

[54] Ke X, Shao X, Wang J, Zhuo J. The effect of individual nursing on anxiety and depression in patients with temporomandibular disorders. Contrast Media & Molecular Imaging. 2022; 2022: 1–5.

[55] Minakuchi H, Kuboki T, Maekawa K, Matsuka Y, Yatani H. Self-reported remission, difficulty, and satisfaction with nonsurgical therapy used to treat anterior disc displacement without reduction. Oral Surgery, Oral Medicine, Oral Pathology, Oral Radiology, and Endodontology. 2004; 98: 435–440.

[56] Schiffman EL, Look JO, Hodges JS, Swift JQ, Decker KL, Hathaway KM, *et al*. Randomized effectiveness study of four therapeutic strategies for TMJ closed lock. Journal of Dental Research. 2007; 86: 58–63.

[57] Kraaijenga S, van der Molen L, van Tinteren H, Hilgers F, Smeele L. Treatment of myogenic temporomandibular disorder: a prospective randomized clinical trial, comparing a mechanical stretching device (TheraBite (R)) with standard physical therapy exercise. CRANIO—The Journal of Craniomandibular & Sleep Practice. 2014; 32: 208–216.

[58] Nagata K, Maruyama H, Mizuhashi R, Morita S, Hori S, Yokoe T, *et al*. Efficacy of stabilisation splint therapy combined with non-splint multimodal therapy for treating RDC/TMD axis I patients: a randomised controlled trial. Journal of Oral Rehabilitation. 2015; 42: 890–899.

[59] Sailaja S, Daruri S, Lavanya R, Ravali C, Dhanabalan S, Bojanapu S, *et al*. Comparative study of mouth exercise program and with combination of ultrasound therapy in temporomandibular disorder. Journal of Indian Academy of Oral Medicine and Radiology. 2022; 34: 432.

[60] Brandao R, Mendes CMC, Lopes TD, Brandao RA, de Sena EP. Neurophysiological aspects of isotonic exercises in temporomandibular joint dysfunction syndrome. CODAS. 2021; 33: e20190218.

[61] Lucas C, Branco I, Silva M, Alves P, Pereira ÂM. Benefits of manual therapy in temporomandibular joint dysfunction treatment. Annals of Medicine. 2018; 50: S167–S168.

[62] Brignardello-Petersen R. Self-management interventions may be more effective than usual care for treating patients with chronic orofacial pain. The Journal of the American Dental Association. 2019; 150: e121.

[63] Haketa T, Kino K, Sato F, Ishikawa T, Sugisaki M. Randomized clinical study of initial treatment for temporomandibular disorder ADDWOR. Journal of Dental Research. 2007.

[64] Kim H, Kim K, Kim M, Lee YJ, Ha I, Shin B, *et al*. Clinical research on the clinical effectiveness and cost-effectiveness of Chuna manual therapy for temporomandibular disorder: a study protocol for a multicenter randomized controlled trial. European Journal of Integrative Medicine. 2019; 27: 27–33.

[65] Incorvati C, Romeo A, Fabrizi A, Defila L, Vanti C, Gatto MRA, *et al*. Effectiveness of physical therapy in addition to occlusal splint in myogenic temporomandibular disorders: protocol of a randomised controlled trial. BMJ Open. 2020; 10: e038438.

[66] Kim J, Park KS, Lee YJ, Kim K, Cho J, Ha I. Efficacy, safety, and economic assessment of hominis placental pharmacopuncture for chronic temporomandibular disorder: a protocol for a multicentre randomised controlled trial. Trials. 2020; 21: 525.

[67] Roth PM, Thrash WJ. Effect of transcutaneous electrical nerve stimulation for controlling pain associated with orthodontic tooth movement. American Journal of Orthodontics and Dentofacial Orthopedics. 1986; 90: 132–138.

[68] Geisser ME, Wiggert EA, Haig AJ, Colwell MO. A randomized, controlled trial of manual therapy and specific adjuvant exercise for chronic low back pain. the Clinical Journal of Pain. 2005; 21: 463–470.

[69] Galindez-Ibarbengoetxea X, Setuain I, Ramirez-Velez R, Andersen LL, Gonzalez-Izal M, Jauregi A, *et al*. Immediate effects of osteopathic treatment versus therapeutic exercise on patients with chronic cervical pain. Alternative Therapies in Health and Medicine. 2018; 24: 24–32.

[70] Klobas L, Axelsson S, Tegelberg A. Effect of therapeutic jaw exercise on temporomandibular disorders in individuals with chronic whiplash-associated disorders. Acta Odontologica Scandinavica. 2006; 64: 341–347.

[71] Doerfler LF, Rugh JD. Voluntary mandibular retrusion exercises and facial-pain. Journal of Dental Research. 1984; 63: 288–288.

[72] Magnusson T, Syrén M. Therapeutic jaw exercises and interocclusal appliance therapy. A comparison between two common treatments of temporomandibular disorders. Swedish Dental Journal. 1999; 23: 27–37.

[73] Kalamir A, Bonello R, Graham P, Vitiello AL, Pollard H. Intraoral myofascial therapy for chronic myogenous temporomandibular disorder: a randomized controlled trial. Journal of Manipulative and Physiological Therapeutics. 2012; 35: 26–37.

[74] Tavera AT, Montoya MC, Calderón EF, Gorodezky G, Wixtrom RN. Approaching temporomandibular disorders from a new direction: a randomized controlled clinical trial of the TMDes ear system. CRANIO. 2012; 30: 172–182.

[75] Kokkola O, Suominen AL, Qvintus V, Myllykangas R, Lahti S, Tolvanen M, *et al*. Efficacy of stabilisation splint treatment on the oral health‐related quality of life—a randomised controlled one‐year follow‐up trial. Journal of Oral Rehabilitation. 2018; 45: 355–362.

[76] Zwiri AM, Ahmad W, Asif JA, Phaik KS, Husein A, Kassim NK, *et al*. A randomized controlled trial evaluating the levels of the biomarkers hs-CRP, IL-6, and IL-8 in patients with temporomandibular disorder treated with LLLT, traditional conservative treatment, and a combination of both. International Journal of Environmental Research and Public Health. 2022; 19: 8987.

[77] Lindfors E, Magnusson T, Ernberg M. Effect of therapeutic jaw exercises in the treatment of masticatory myofascial pain: a randomized controlled study. Journal of Oral & Facial Pain and Headache. 2020; 34: 364–373.

[78] Delgado de la Serna P, Plaza-Manzano G, Cleland J, Fernández-de-las-Peñas C, Martín-Casas P, Díaz-Arribas MJ. Effects of cervico-mandibular manual therapy in patients with temporomandibular pain disorders and associated somatic tinnitus: a randomized clinical trial. Pain Medicine. 2020; 21: 613–624.

[79] Coskun Benlidayi I, Salimov F, Kurkcu M, Guzel R. Kinesio taping for temporomandibular disorders: Single-blind, randomized, controlled trial of effectiveness. Journal of Back and Musculoskeletal Rehabilitation. 2016; 29: 373–380.

[80] Maluf SA, Moreno BGD, Crivello O, Cabral CMN, Bortolotti G, Marques AP. Global postural reeducation and static stretching exercises in the treatment of myogenic temporomandibular disorders: a randomized study. Journal of Manipulative and Physiological Therapeutics. 2010; 33: 500–507.

[81] Olbort C, Pfanne F, Schwahn C, Bernhardt O. Training of the lateral pterygoid muscle in the treatment of temporomandibular joint disc displacement with reduction: a randomised clinical trial. Journal of Oral Rehabilitation. 2023; 50: 921–930.

Supplementary Table 3. The detailed contents of home-based rehabilitation and control group in each study.

|  | Author (Year) | Home-based rehabilitation | Control group |
| --- | --- | --- | --- |
| Home-based rehabilitation versus Occlusal splints | Truelove 2006 | Dentist prescribed, conservative and reversible self-care strategies that required the dentist to follow a standardized treatment checklist that identifies all treatment recommendations (jaw relaxation, reduction of parafunction, thermal packs, NSAID, passive opening stretches and suggestions about stress reduction); we prescribed selfcare strategies to all subjects, and we discouraged treatments such as narcotic analgesics, antidepressant medications and use of a non-study prescribed splint. | Hard splint: hard acrylic heat- (dental laboratory-) processed flat plane maxillary splint.  Soft splint: soft thermoplastic vinyl athletic mouthguard splint. |
|  | Haketa 2010 | Participants performed manual jaw-opening exercises by themselves, according to the following protocol: As a warm-up, the individual repeated small mouth-opening and -closing movements several times. Then, the individual placed his/her fingertips on the edge of the mandibular anterior teeth and slowly pulled the mandible down until pain occurred on the TMJ-affected side. All participants in both groups were prescribed NSAID. | Splints: the splint was a 1.5-mm-thick hard, clear acrylic sheet that was vacuum-adapted to the maxillary cast. |
|  | Niemelä 2012 | Patients were instructed to perform a standardized  program for masticatory muscle exercises as described by Carlsson and Magnusson. At the beginning of the training program, active mouth openings, latero-trusive movements and protrusive movements were performed. The mandible was held in the maximal positions for a few seconds on each movement. Thereafter, these movements were made towards resistance (using patient’s own fingers). After jaw exercises, the patients were suggested to open the jaw wide, stretching it with fingers a few times for 10–20 s. These movements were repeated 7–10 times per training sessions, and the sessions were performed 2–3 times per day. The patients received written instructions, and the movements were also demonstrated by the dentist before the treatment and reprised if necessary. The instructions for masticatory muscle exercises were given by the same dentist (KS) at the first visit. The stabilisation splint treatments were performed by two other dentists who were carefully instructed in the treatment method. | Home-based rehabilitation + stabilization splint (The stabilisation splints were made of heat-cured acrylic). |
|  | Ficnar 2013 | Included the use of self-exercises (muscle exercise form according to Prof. Schulte, self-massage techniques, mouth opening exercises), medication-based therapy using NSAID, muscle relaxants as well as manual therapy. | SB: semi-finished occlusal appliance  SS: laboratory-made occlusal appliance |
|  | Costa 2015 | Verbal and written instructions about TMD etiology and prognosis, diet modification in order to avoid hard foods, use of reminders to avoid parafunctional habits, relaxation exercises for the jaw muscles, application of a heating pad on painful muscles followed by stretching and self-massage, instructions about sleep hygiene, and incentives for social and aerobic activities. The suggested frequency for application of heating pads on painful muscles, followed by stretching and self-massage, was three to five times a week for at least 30 minutes each. One therapist was available full time to clarify any doubts related to the instructions. All instructions were clearly repeated during each assessment appointment. | splints |
|  | Qvintus 2015 | Patients were instructed to perform a standardized  program for masticatory muscle exercises as described by Carlsson and Magnusson. At the beginning of the training program, active mouth openings, latero-trusive movements and protrusive movements were performed. The mandible was held in the maximal positions for a few seconds on each movement. Thereafter, these movements were made towards resistance (using patient’s own fingers). After jaw exercises, the patients were suggested to open the jaw wide, stretching it with fingers a few times for 10–20 s. These movements were repeated 7–10 times per training sessions, and the sessions were performed 2–3 times per day. The patients received written instructions, and the movements were also demonstrated by the dentist before the treatment and reprised if necessary. The instructions for masticatory muscle exercises were given by the same dentist (KS) at the first visit. The stabilisation splint treatments were performed by two other dentists who were carefully instructed in the treatment method. | Home-based rehabilitation + stabilization splint (The stabilisation splints were made of heat-cured acrylic). |
|  | de Resende 2019①② | Explaining the etiology of TMD and possible harmful and parafunctional habits, such as biting a pen, nails, mouth corners; chewing gum; conscious teeth tightening; and wide opening of the mouth for eating and yawning. The importance of physical exercises, avoiding caffeinated drinks at night, body posture, and a good quality of sleep was also advised. Personalized counseling was done in 30 m sessions initially and reinforced after 15 days. | Splints: thermopolymerizable acrylic resin (Vipi Cril Plus, Vipi Produtos Odontológicos, Pirassununga, SP, Brazil) |
|  | Melo 2020①② | An investigation was made into habits and other factors that might be responsible for the aetiology of the patient’s dysfunction, and then a series of orientated guidelines for each case were developed that individualize treatment according to personal needs. In addition, general characteristics about the disease were clarified, so that patients understood their condition and felt able to manage it themselves. At the end of the consultation, the patient received a written booklet with dietary guidelines, physical exercises, deleterious habits, instructions on correct mandibular function, posture and sleep hygiene. | Splints; |
|  | Wanman 2020 | Home regime of jaw exercises. They were instructed to do two different trainings. The 1st type of exercise was jaw opening and closing movements daily for 5 minutes after each meal with the mandible and the head of the TMJ in a slightly protruded position. This exercise should not produce any clicking sounds. The 2nd type of exercise was isometric exercises; this entailed jaw opening and jaw protrusion against resistance with the hand for 10 seconds with 10 repetitions each daily. Participants received a training brochure with photos of the exercises and detailed information on frequency and exercise time. The brochure also included a diary. | Splints: resilient bite splint, 4 mm thick BIOPLAST® (Scheu Dental GmbH) produced in a BIOSTAR® heat and vacuum press (Scheu Dental GmbH); |
|  | Peixoto 2021① | Patients received verbal and written guidelines, which were reinforced within 15 days from the first consultation. General characteristics of the dysfunction were clarified, so that they would feel able to self-manage themselves. At the end of the consultation, the patients received a flyer with dietary guidelines and recommendations on physical exercises, harmful habits, posture, and sleep hygiene. | Splints |
|  | Ram 2021① | Patients were educated regarding the diagnosis and  generally favorable prognosis of TMD when appropriate which included reassurance that TMD is a typically benign condition and self‑limiting in the vast majority of cases. Patients were educated regarding the biopsychosocial etiology of TMD, sleep practices, time‑limited use of analgesics, anatomy, and functions of TMJ and associated musculature. Patients were educated regarding identification, monitoring, and avoidance of any parafunctional behavior that can exacerbate the pain and were made conscious to avoid daytime clenching, clicking, or grinding of teeth. Patients were advised to avoid unilateral chewing, excessive talking, and chewing gum, to take proper rest and sleep, to do deep breathing exercises. Advised a pain‑free diet for 2 weeks followed by a review to check the tolerance to firmer consistency food. Instructed to apply moist heat to the area of discomfort for 10 min each time for 2–3 times/day. | Stabilization splints: Stabilization splints were constructed by adapting ethylene‑vinyl acetate sheets (2 mm thickness) on  maxillary casts using a vacuum form machine and occlusal surfaces were modified with auto polymerizing acrylic resin to incorporate the contact of all mandibular teeth in centric relation. |
|  | Gikić 2021 | Patients received written materials with information about the nature of temporomandibular disorders (what they are, why they occur, what is the anatomy of the masticatory muscles and temporomandibular joint) and a pictorial presentation and explanation of physical therapy exercises with presentation and review at each follow-up appointment. All patients were required to follow the same home-exercise programme which included exercises for passive and active stretching, joint mobilisation, passive extension and translational movements to the right, left and forward. This procedure is associated with massage of the jaw elevator muscles (temporal muscle and masseter muscle). In addition to the written material, they received a thorough oral explanation of the present temporomandibular disorder with an emphasis on education about the nature of the present pain and ways to deal with it. Exercises were to be performed twice a day (morning and evening) with 10–20 repetitions of each exercise. At each follow-up meeting, patients were required to explain how they were performing exercises and the examiner (IZA) repeated the instructions and corrected them if they were doing something wrong. | Patients received a stabilisation splint and only basic information about the nature of the diagnosis and the type of therapeutic device they will receive. The stabilisation splint was made of hard acrylic (Resilit-S, Erkodent) in the upper jaw in a centric relation position, with a thickness of 1.5 mm at the level of the first molar. It was made on the plaster model of the upper jaw in the ARTEX articulator. Participants were instructed to wear the splint only at night during sleep.  All stabilisation splints were made by the same dental technician, and had a smooth and flat surface, with a canine guidance occlusal scheme. They were adjusted by the clinician (IZA) so that the antagonistic teeth occluded simultaneously with the splint surface, and if necessary, further adjusted at follow-up appointments. |
|  | Olbort 2023 | Patients received muscle training which included: coordination training for lateral mandibular movement and static stretching and isometric contraction exercises of the LPM to strengthen and restore physiological lateral mandibular movement. A supervisor demonstrated the exercises at the dental office. In addition, patients received an exercise DVD (‘Trainingsprogramm Kiefergelenk’ CMD Kompetenzzentrum Westlausitz, Dr Pfanne) for self-instruction. The muscle exercises were to be performed once a day with 5–6 repetitions per side. | The upper and lower jaw impressions were recorded with an intraoral scanner (3Shape Trios 3, straumanngroup). The arbitrary hinge axis position was determined with a facebow (artex facebow, ammanngirrbach). A paraocclusal tray was used to connect the facebow with the intraoral scan. In addition, a digital jaw registration (zebris JMA-Optic jaw registration system) was performed and transmitted to the dental laboratory. |
| Home-based rehabilitation versus Home-based rehabilitation + Manual therapy | Craane 2012 | Patients were extensively informed about normal jaw function and that overuse, misuse, or parafunction could enhance or provoke their complaints. They received instructions to keep the jaw muscles relaxed, and to avoid non-functional tooth contacts and excessive mouth opening. The instructions were given orally by one investigator in a standard way. In addition, all the patients received a brochure to reinforce and re-study these instructions at home. | Home-based rehabilitation + manual therapy (joint mobilization, and massage). |
|  | Tuncer 2013 | Patient education, self-massage and stretching for masticatory and neck muscles, active jaw movement exercises and coordination exercises for TMJ, head and general posture correction training and strengthening exercises. Each home-based rehabilitation session was 30 minutes, and three treatments per week were administered by themselves. | Home-based rehabilitation + manual therapy (deep friction massage, myofascial release techniques for masticator and neck muscles, guided opening and closing movements for TMJ, joint distraction, anterior and/or lateral glide to stretch the TMJ capsule for disk-condyle accommodation, stabilization and coordination exercises for TMJ, gentle isometric tension exercises  against resistance, and mobilization of cervical spines). |
|  | Corum 2018 | Patients were informed about the causes and associations of complaints, treatment options, and management of symptoms to effectively contribute to their care during treatment and follow up. The education program consisted of information about resting the TMJ and masticatory muscles by limiting jaw activity (*e.g.*, reduced talking, chewing, yawning), parafunctional habit modification, postural correction, reducing stress, anxiety and fear, emphasizing a soft diet, and applying heat and/or ice therapy. | Home-based rehabilitation + Manual therapy (cervical spinal manipulation plus neck exercise) |
|  | Nagata 2018 | Self-exercise, CBT, and education for TMD. Following our previous RCT regarding the splint therapy, we did not use any type of splints in this study. The self-exercise consisted of two types of exercise for the mandibular jaw. One exercise pulled down on a patient’s bilateral lower last molars with their secondary fingers, while opening the jaw to the greatest possible extent (molar pulldown type). The other exercise comprised simplified myo-functional therapy, combined with (1) maximum mouth opening, (2) clenching, (3) protrusion of the lip, (4) maximum mouth opening and maximum tongue protrusion without use of the patient’s fingers; this approach was advocated by Imai. Instructions were to execute simplified myo-functional therapy every hour throughout the day. The patients were guided to perform two types of self-exercise, to the extent that weak pain was felt every time. If sufficient recovery of the mouth opening (<40 mm) had been achieved, patients were prescribed a reduction of exercise strength. | Home-based rehabilitation + Manual therapy (Jog-manipulation) |
|  | Delgado 2020 | Mobility, postural education, and motor control exercises of the TMJ, the tongue, and the neck; instructions for resting jaw position, head/neck position, and posture were provided. | Home-based rehabilitation + Manual therapy (techniques focusing on the TMJ and the masticatory and cervical musculature). |
|  | de Resende 2019③ | Explaining the etiology of TMD and possible harmful and parafunctional habits, such as biting a pen, nails, mouth corners; chewing gum; conscious teeth tightening; and wide opening of the mouth for eating and yawning. The importance of physical exercises, avoiding caffeinated drinks at night, body posture, and a good quality of sleep was also advised. Personalized counseling was done in 30 m sessions initially and reinforced after 15 days. | Home-based rehabilitation + Manual therapy (manual therapy was conducted by a trained researcher and involved heating agents and exercises) |
|  | Melo 2020③ | An investigation was made into habits and other factors that might be responsible for the aetiology of the patient’s dysfunction, and then a series of orientated guidelines for each case were developed that individualize treatment according to personal needs. In addition, general characteristics about the disease were clarified, so that patients understood their condition and felt able to manage it themselves. At the end of the consultation, the patient received a written booklet with dietary guidelines, physical exercises, deleterious habits, instructions on correct mandibular function, posture and sleep hygiene. | Home-based rehabilitation + Manual therapy |
|  | Peixoto 2021③ | Patients received verbal and written guidelines, which were reinforced within 15 days from the first consultation. General characteristics of the dysfunction were clarified, so that they would feel able to self-manage themselves. At the end of the consultation, the patients received a flyer with dietary guidelines and recommendations on physical exercises, harmful habits, posture, and sleep hygiene. | Home-based rehabilitation + Manual therapy |
|  | Ram 2021② | Patients were educated regarding the diagnosis and  generally favorable prognosis of TMD when appropriate which included reassurance that TMD is a typically benign condition and self‑limiting in the vast majority of cases. Patients were educated regarding the biopsychosocial etiology of TMD, sleep practices, time‑limited use of analgesics, anatomy, and functions of TMJ and associated musculature. Patients were educated regarding identification, monitoring, and avoidance of any parafunctional behavior that can exacerbate the pain and were made conscious to avoid daytime clenching, clicking, or grinding of teeth. Patients were advised to avoid unilateral chewing, excessive talking, and chewing gum, to take proper rest and sleep, to do deep breathing exercises. Advised a pain‑free diet for 2 weeks followed by a review to check the tolerance to firmer consistency food. Instructed to apply moist heat to the area of discomfort for 10 min each time for 2–3 times/day. | Home-based rehabilitation + Manual therapy (Muscle energy technique: post isometric relaxation and reciprocal inhibition) |
| Home-based rehabilitation versus Physical factor therapy | Cavalcanti 2016 | Hot packs thrice a day, morning, afternoon, and evening, for 15 min, exercise of opening and closing the mouth, twice a day, myo-relaxing and anti-inflammatory drug administration | LLLT: 780nm laser, dose of 35.0 J/cm^2^, for 20 sec, thrice a week, for 4 weeks. |
|  | Machado 2016 | Instructions to the patients about TMD, myofunctional disorders, and care needed to avoid system overloading; strategies for pain relief such as thermotherapy, massage, and relaxation training; OM exercises, *i.e.*, such as exercises for tongue, lips, and cheeks and jaw muscles (mobility, endurance, muscle strength) and orofacial function training. | LLLT (Model Twin Flex Evolution Laser; MM Optics Ltda, São Carlos, São Paulo) with continuous emission at 780nm wavelength, a power of 60 mW for 40 s, and energy density of 60 ± 1.0 J/cm^2^ was use; |
|  | Patil 2017 | Patients were briefed about modes for handling with TMJ pain through variations in the lifestyle, confronting mechanisms and ergonomic management. They were advised an exercise program comprising of active and passive jaw opening and closing exercises, isometric jaw exercises, jaw stretching exercises and resistive jaw exercises. The participants were instructed to carry out every exercise for six seconds with repetitions for 10 times. These exercises were implemented twice a day for four weeks | TENS (HiDow FDA CLASS II Approved Wireless TENS system): the power was 20 W, with a maximum frequency of 60 Hz and amplitude ranging from 1–10 µA), each therapeutic session lasted for approximately 30 minutes, once in a week for a period of four consecutive weeks. |

NSAID: non-steroidal anti-inflammatory drugs; TMJ: temporomandibular joint; TMD: temporomandibular joint disorders; CBT: cognitive-behavioral therapy; RCT: randomized clinical trials; LLLT: low-level laser irradiation; OM exercise: oral motor exercises; TENS: transcutaneous electrical nerve stimulation; ①②③: the study had more than two groups, which were distinguished by the sequence number ①, ②, and ③.

Supplementary Table 4. Cost comparison between home-based rehabilitation and control group.

|  | Author (Year) | Home-based rehabilitation  Treatment content | Control group |
| --- | --- | --- | --- |
| Home-based rehabilitation versus Splints | Truelove 2006 | Health and exercise recommendations | Hard acrylic heat processed flat plane maxillary splint |
|  | Haketa 2010 | 1. Health and exercise recommendations   2. Amfenac sodium 3 pre a day | 1. 1.5-mm-thick hard clear acrylic sheet splint 2. Amfenac sodium 3 pre a day |
|  | Ficnar 2013 | 1. Health and exercise recommendations   Nonsteroidal anti-inflammatory drugs | SOLUBruxW splint made by malleable thermoplastic |
|  | Costa 2015 | Health and exercise recommendations | Hard acrylic maxillary full-coverage occlusal appliance |
|  | de Resende 2019①② | Health and exercise recommendations | Thermopolymerizable acrylic resin occlusal splints |
|  | Melo 2020①② | A written booklet (with Health Recommendations) | Thermopolymerisable acrylic resin splints (Classic Dental Articles LTDA) |
|  | Wanman 2020 | 1. Training brochure (with home exercise Recommendations) 2. Self-management diary | 4 mm thick BIOPLAST® (Scheu Dental GmbH) resilient bite splint |
|  | Peixoto 2021① | A flyer (with dietary guidelines and recommendations on physical exercises, harmful habits, posture, and sleep hygiene) | Thermopolymerizable acrylic resin full coverage occlusal splints |
|  | Qvintus 2015 | A written instruction | 1. Heat-cured acrylic splints |
|  | Niemelä 2012 | Counselling and instructions for masticatory muscle exercises | Stabilisation splints. |
|  | Ram 2021 | Education for self-management and counseling | Ethylene-vinyl acetate Stabilization splints |
|  | Gikić 2021 | A written instruction | Stabilisation splints. |
|  | Olbort 2023 | An exercise DVD | Stabilisation splints. |
| Home-based rehabilitation versus Home-based rehabilitation + Manual therapy | Craane 2012 | A brochure (with the summary of the treatment modalities) | 9 PT sessions over a 6-week period (twice weekly for 3 weeks and once weekly for the last 3 weeks) |
|  | Tuncer 2013 | Health and exercise recommendations | MT three times a week during the 4 weeks treatment period, each treatment session lasted 30 min. |
|  | Corum 2018 | Health and exercise recommendations | Manual therapy: cervical spinal manipulation plus neck exercise. |
|  | Nagata 2018 | Health and exercise recommendations | Manual therapy by the predefined dentist at the first visit and each subsequent visit of the patients until the restoration of mouth-opening limitation <40 mm |
|  | Delgado 2020 | A self-care book (with health and exercise recommendations). | One month treatment (two sessions the first week and four weekly sessions), multimodal physiotherapy treatment of 30 minutes’ duration |
|  | de Resende 2019③ | Health and exercise recommendations | Manual therapy (Each session lasted 40 min and was performed twice a week for 4 weeks) |
|  | Melo 2020③ | A written booklet (with Health Recommendations) | 1. Manual therapy of 40-min sessions, performed twice a week for 4 weeks. 2. Gel packet at temperatures between 40 °C and 50 °C. |
|  | Peixoto 2021③ | A flyer (with dietary guidelines and recommendations on physical exercises, harmful habits, posture, and sleep hygiene) | Manual therapy of 40-min sessions, performed twice a week for 4 weeks. |
|  | Ram 2021 | Education for self-management and counseling | Muscle energy technique of three times a week for four weeks. |
| Home-based rehabilitation versus Physical factor therapy | Cavalcanti 2016 | 1. Health and exercise recommendations  2. Myo-relaxing  3. Anti-inflammatory drug | Low-level laser (Twin Flex Evolution, MM optics, São Carlos, Brazil) 780 nm laser, dose of 35.0 J/cm2, for 20 sec, thrice a week, for 4 weeks. |
|  | Machado 2016 | Health and exercise recommendations | Low-level laser (Model Twin Flex Evolution Laser; MM Optics Ltda, São Carlos, São Paulo) with continuous emission at 780-nm wavelength, a power of 60 mW for 40 s, and energy density of 60 ± 1.0 J/cm^2^, lasted for 45 min and were held on a weekly basis during the first 60 days and on a biweekly basis thereafter for a total of 12 sessions, totaling a maximum of 9 h in the 120-day period. |
|  | Patil 2017 | Health and exercise recommendations | TENS (HiDow FDA CLASS II Approved Wireless TENS system): the power was 20 W, with a maximum frequency of 60 Hz and amplitude ranging from 1–10 µA), each therapeutic session lasted for approximately 30 minutes, once in a week for a period of four consecutive weeks. |
|  | Peixoto 2021② | A flyer (with dietary guidelines and recommendations on physical exercises, harmful habits, posture, and sleep hygiene) | Chinese scalp acupuncture: (Disposable and sterile needles for acupuncture, 0.25×30. (DongBang Acupuncture, Inc.)). 8 sessions of 40 minutes, twice a week, totaling 4 weeks. |

①②③: the study had more than two groups, which were distinguished by the sequence number ①, ② and ③.


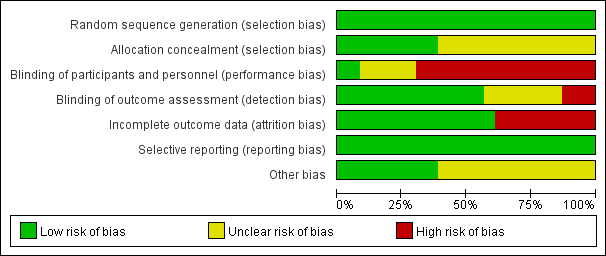


Supplementary Fig. 1. Risk of bias graph Review authors’ judgments about each risk of bias item for each included study.


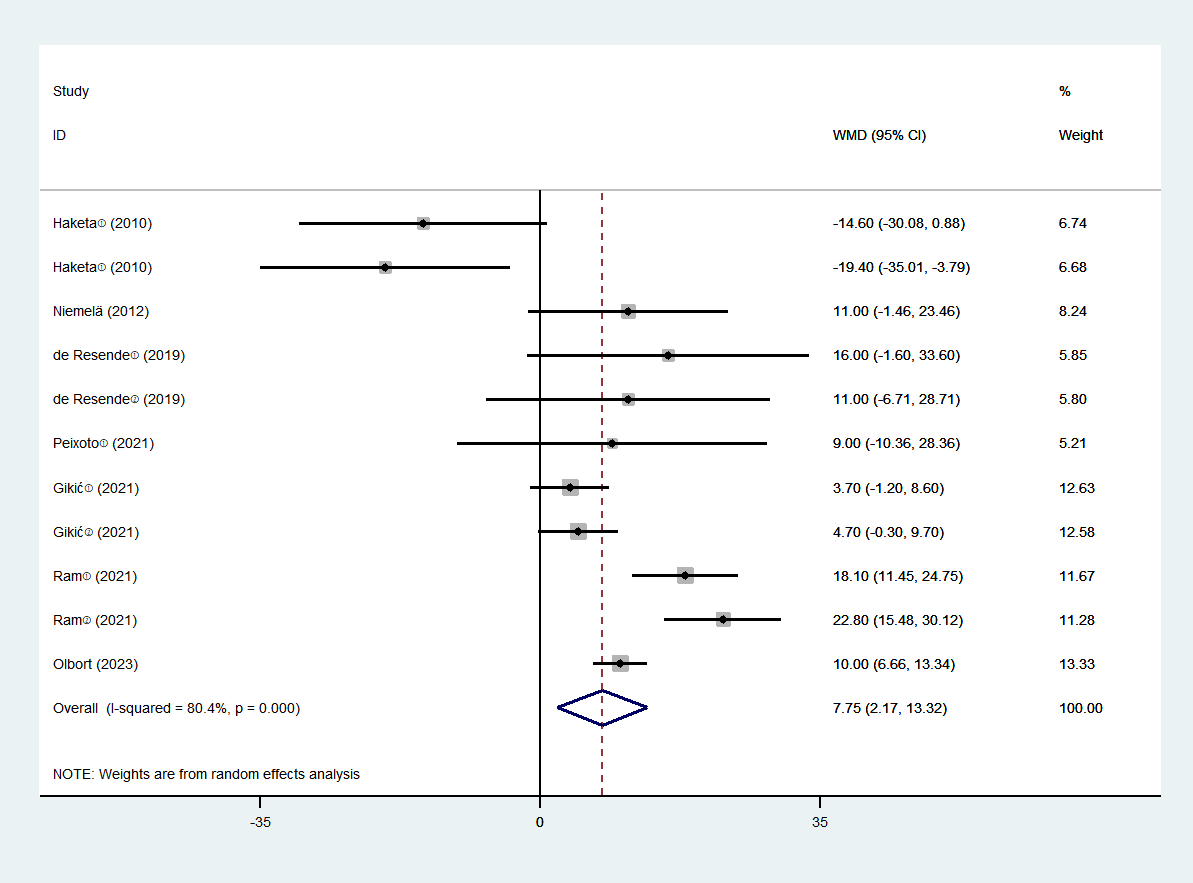


Supplementary Fig. 2. Forest plot of pain relief. Comparing home-based rehabilitation with splints in pain (mean difference, MD) at short term follow-up. Pooled mean differences calculated by random effects model.


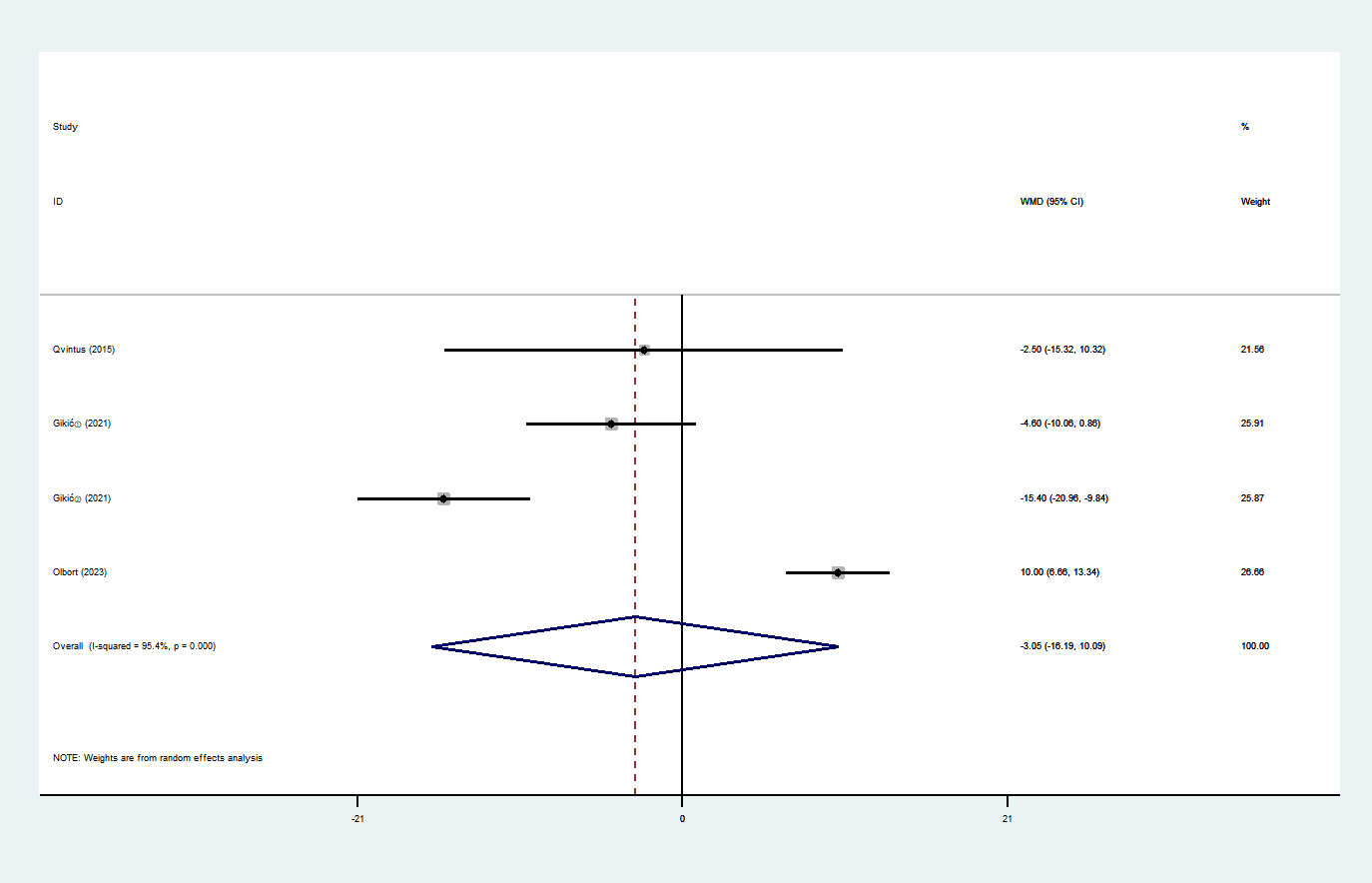


Supplementary Fig. 3. Forest plot of pain relief. Comparing home-based rehabilitation with splints in pain (mean difference, MD) at long term follow-up. Pooled mean differences calculated by random effects model.


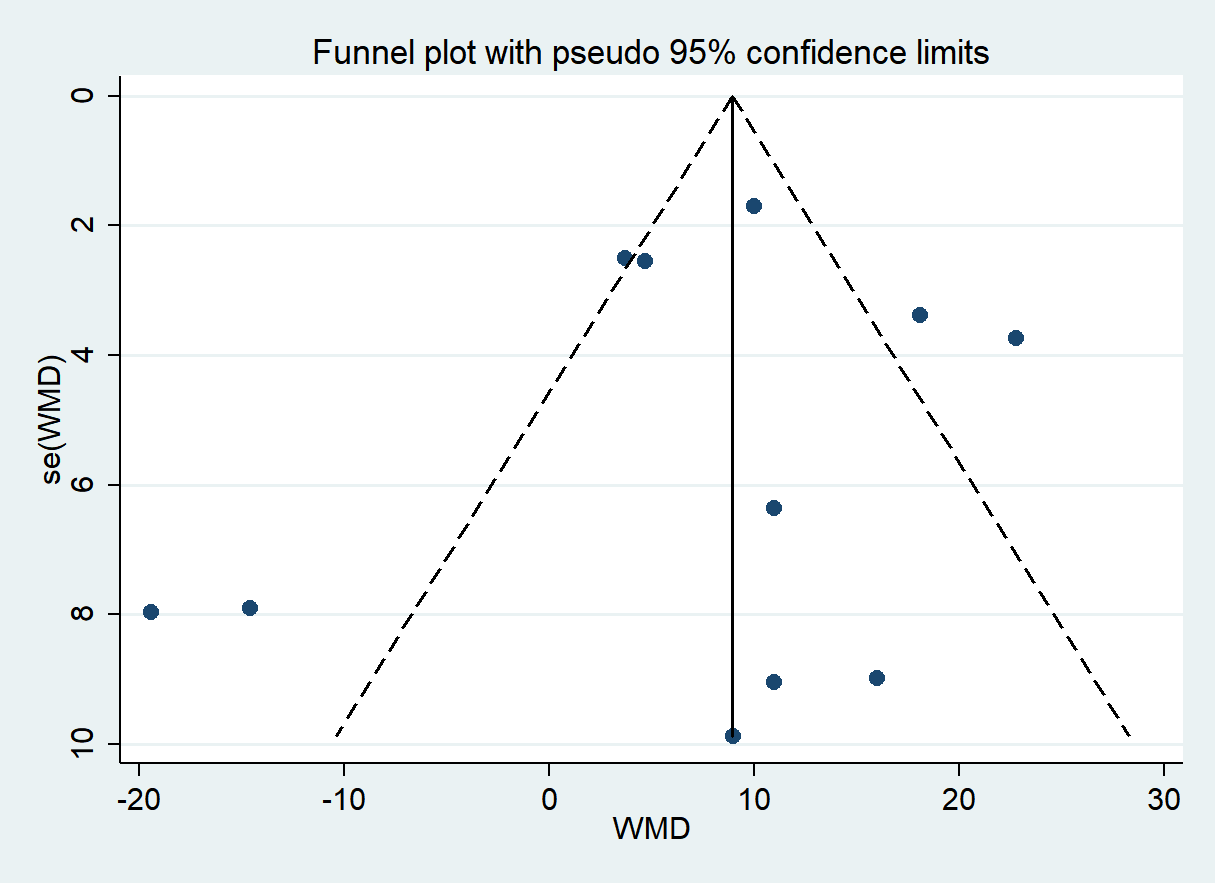


Supplementary Fig. 4. Publication bias heterogeneity funnel plot for pain intensity (short term follow-up). A funnel plot was used to assess the risk of publication bias. The diagonal lines represent the 95% confidence limits. Se: standard error, MD: mean difference. A random-effects model was used.


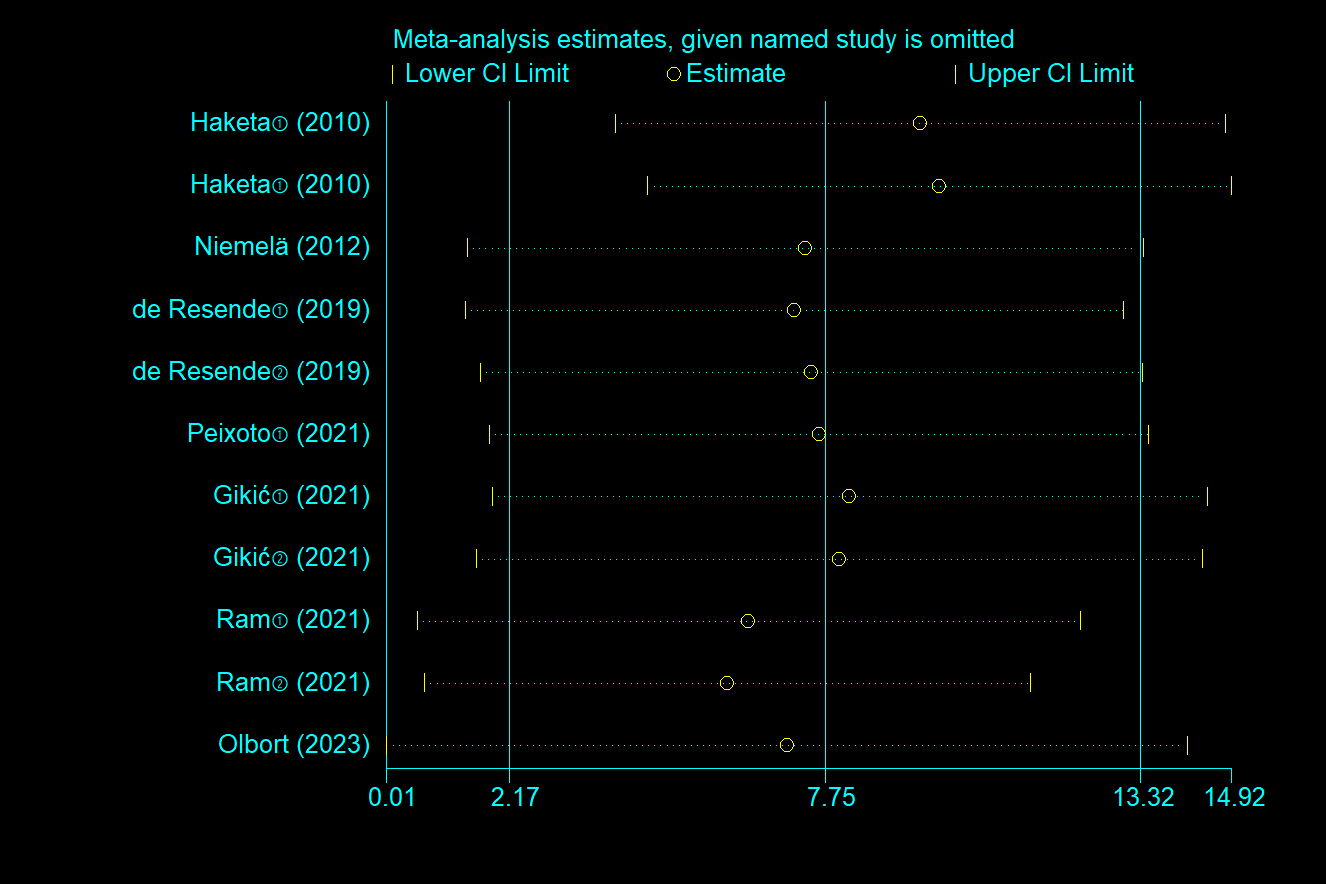


Supplementary Fig. 5. Exclusion sensitivity plot for pain. Comparing home-based rehabilitation with occlusal splints in pain (mean difference, MD) at short term follow-up. Pooled mean differences calculated by random-effects model.


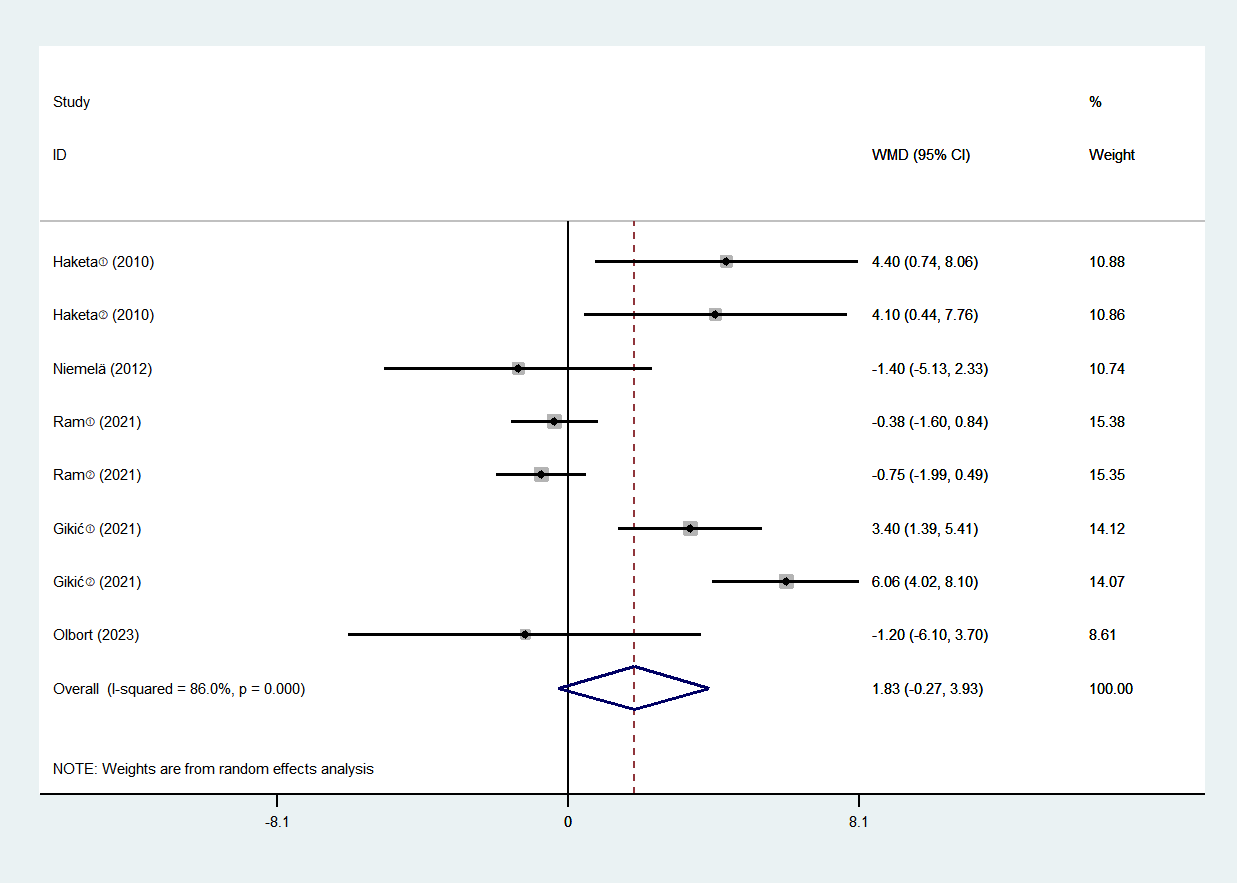


Supplementary Fig. 6. Forest plot of maximal mouth opening at short term follow-up. Comparing home-based rehabilitation with occlusal splints in maximal mouth opening (mean difference, MD) at short term follow-up. Pooled mean differences calculated by random effects model.


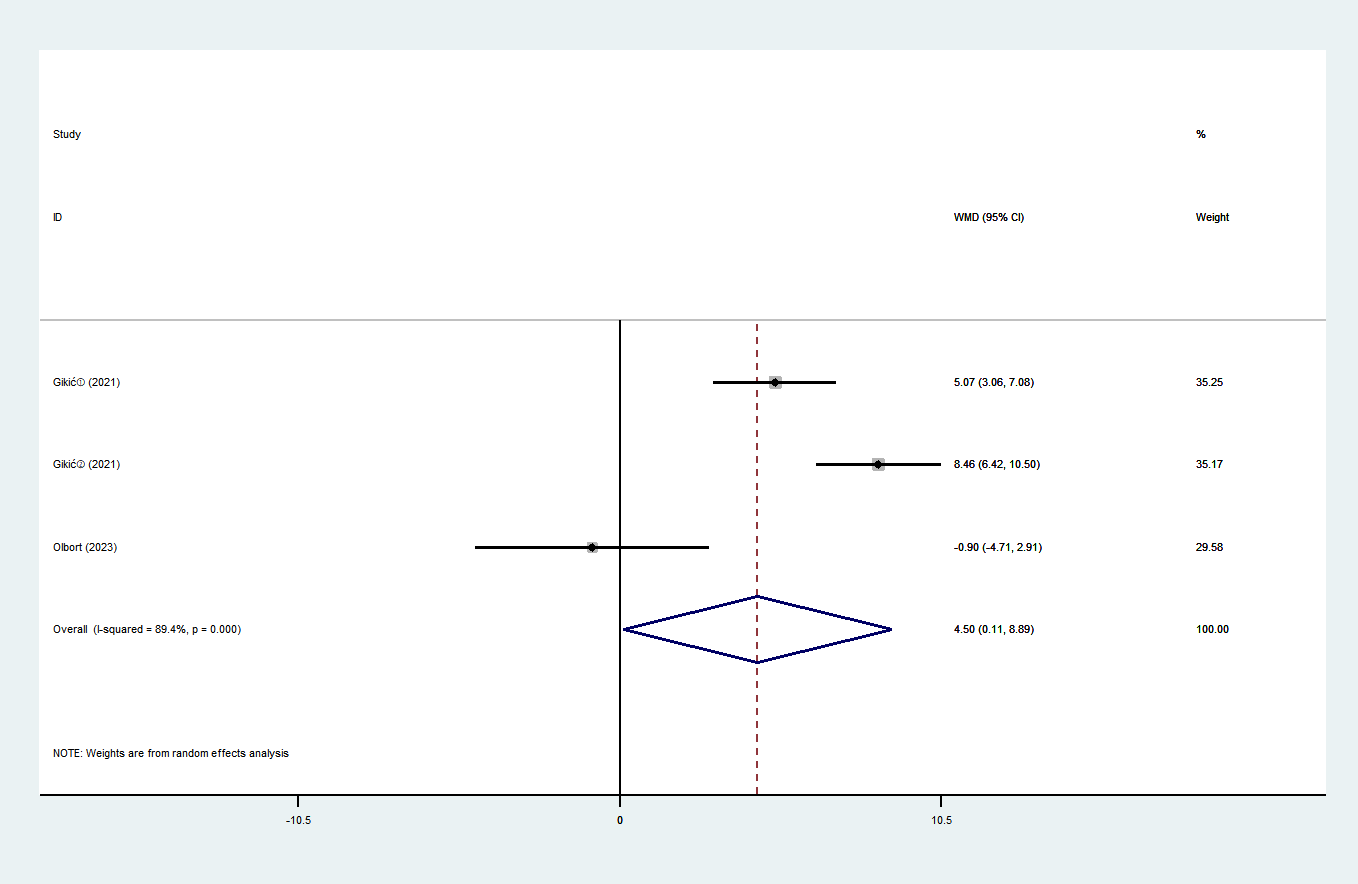


Supplementary Fig. 7. Forest plot of maximal mouth opening at short term follow-up. Comparing home-based rehabilitation with occlusal splints in maximal mouth opening (mean difference, MD) at long term follow-up. Pooled mean differences calculated by random effects model.


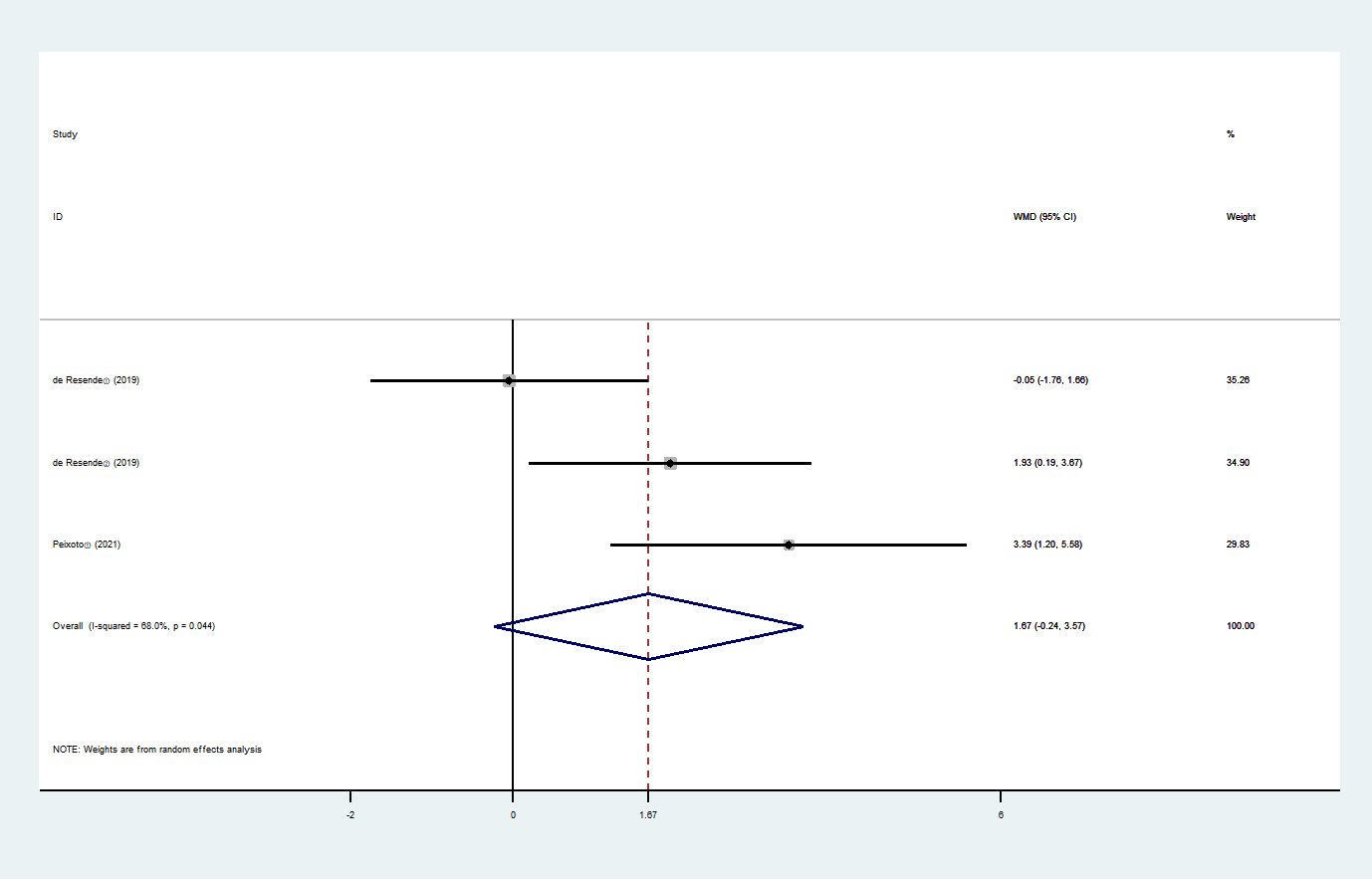


Supplementary Fig. 8. Forest plot of sleep quality at short term follow-up. Comparing home-based rehabilitation with splints in sleep quality (mean difference, MD) at short term follow-up. Pooled mean differences calculated by random effects model.


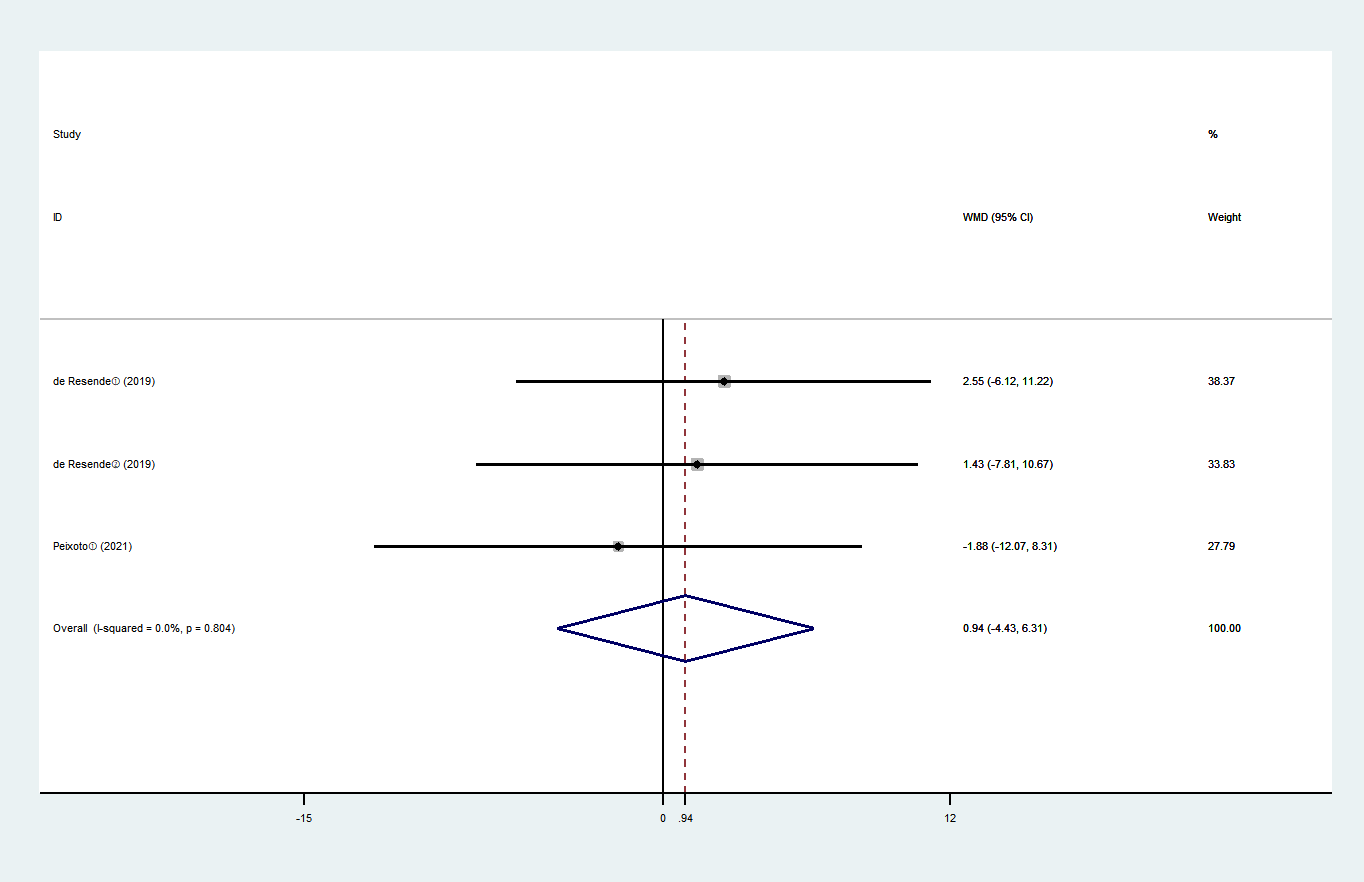


Supplementary Fig. 9. Forest plot of life quality (psychological) at short term follow-up. Comparing home-based rehabilitation with splints in quality of life (mean difference, MD) at short term follow-up. Pooled mean differences calculated by fixed effects model.


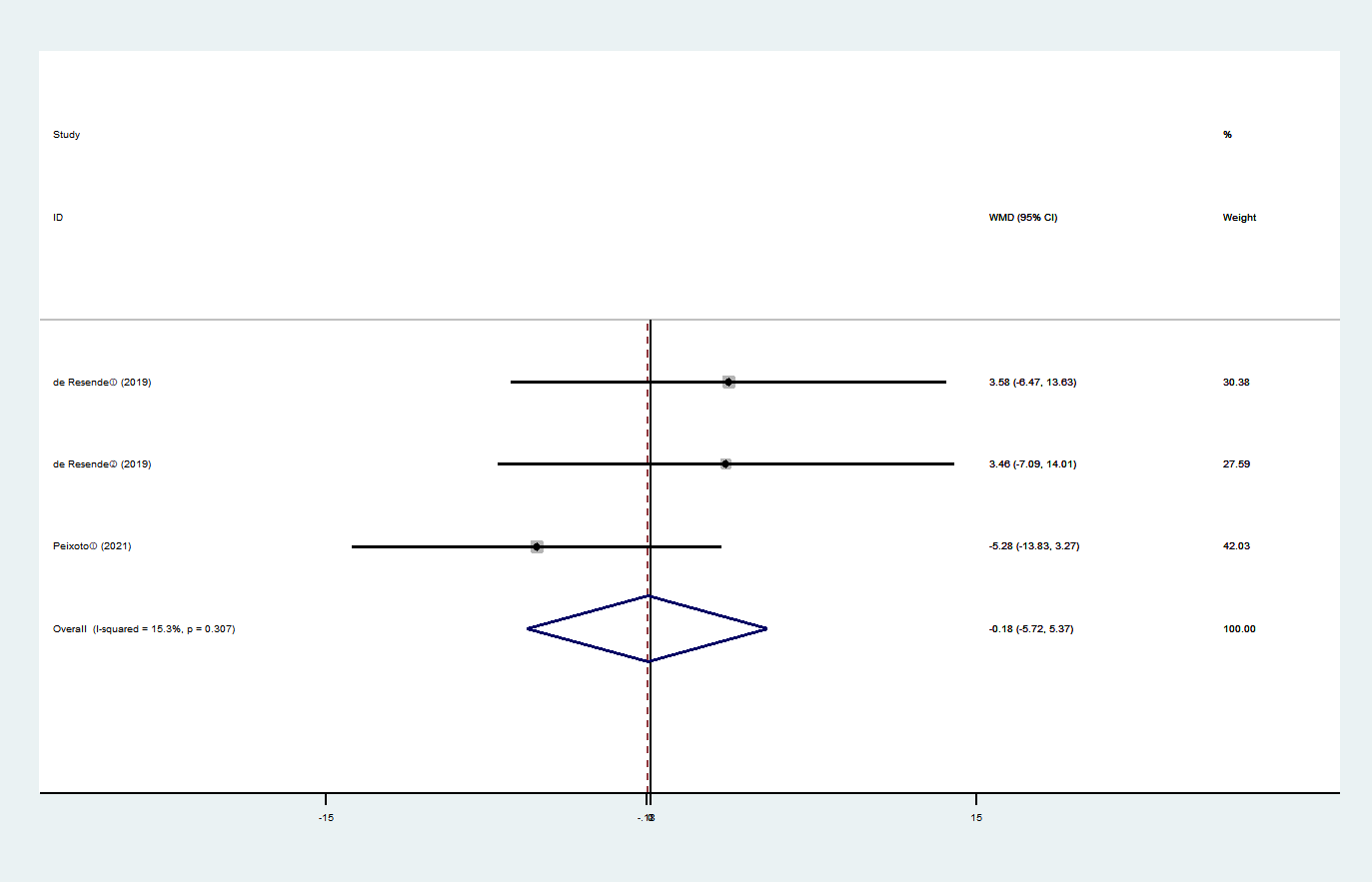


Supplementary Fig. 10. Forest plot of life quality (general) at short term follow-up. Comparing home-based rehabilitation with splints in quality of life (mean difference, MD) at short term follow-up. Pooled mean differences calculated by fixed effects model.


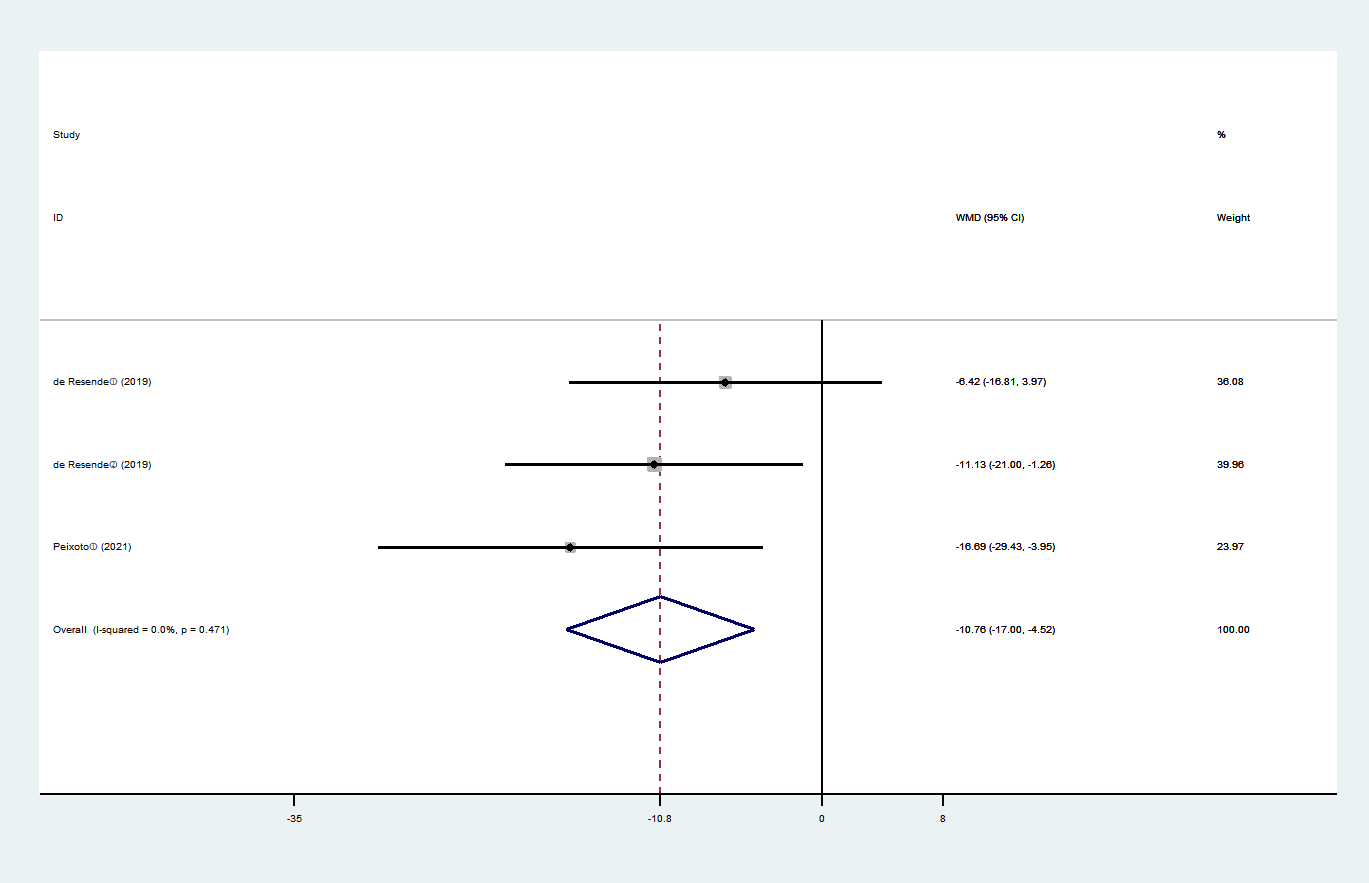


Supplementary Fig. 11. Forest plot of life quality (social) at short term follow-up. Comparing home-based rehabilitation with splints in quality of life (mean difference, MD) at short term follow-up. Pooled mean differences calculated by fixed effects model.


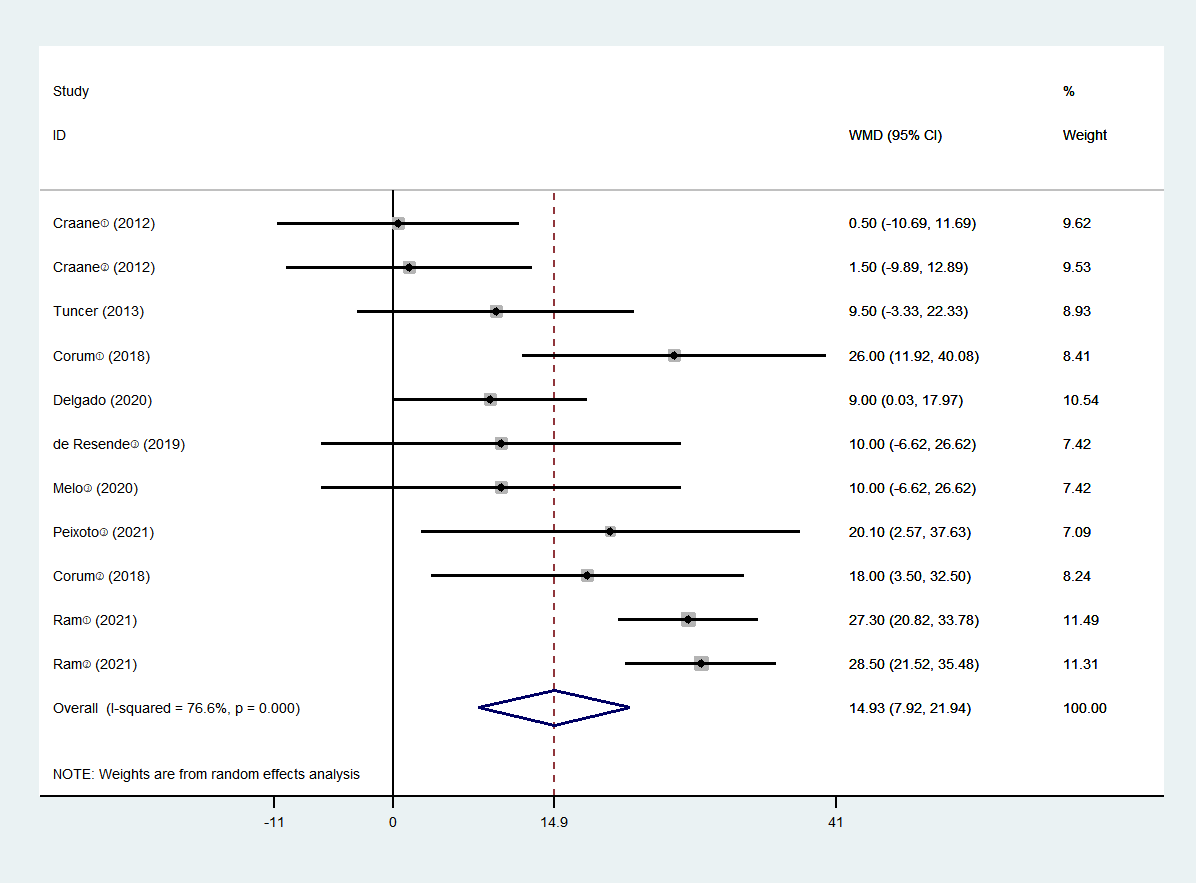


Supplementary Fig. 12. Forest plot of pain relief. Comparing home-based rehabilitation with home-based rehabilitation plus manual therapy in pain (mean difference, MD) at short term follow-up. Pooled mean differences calculated by random-effects model.


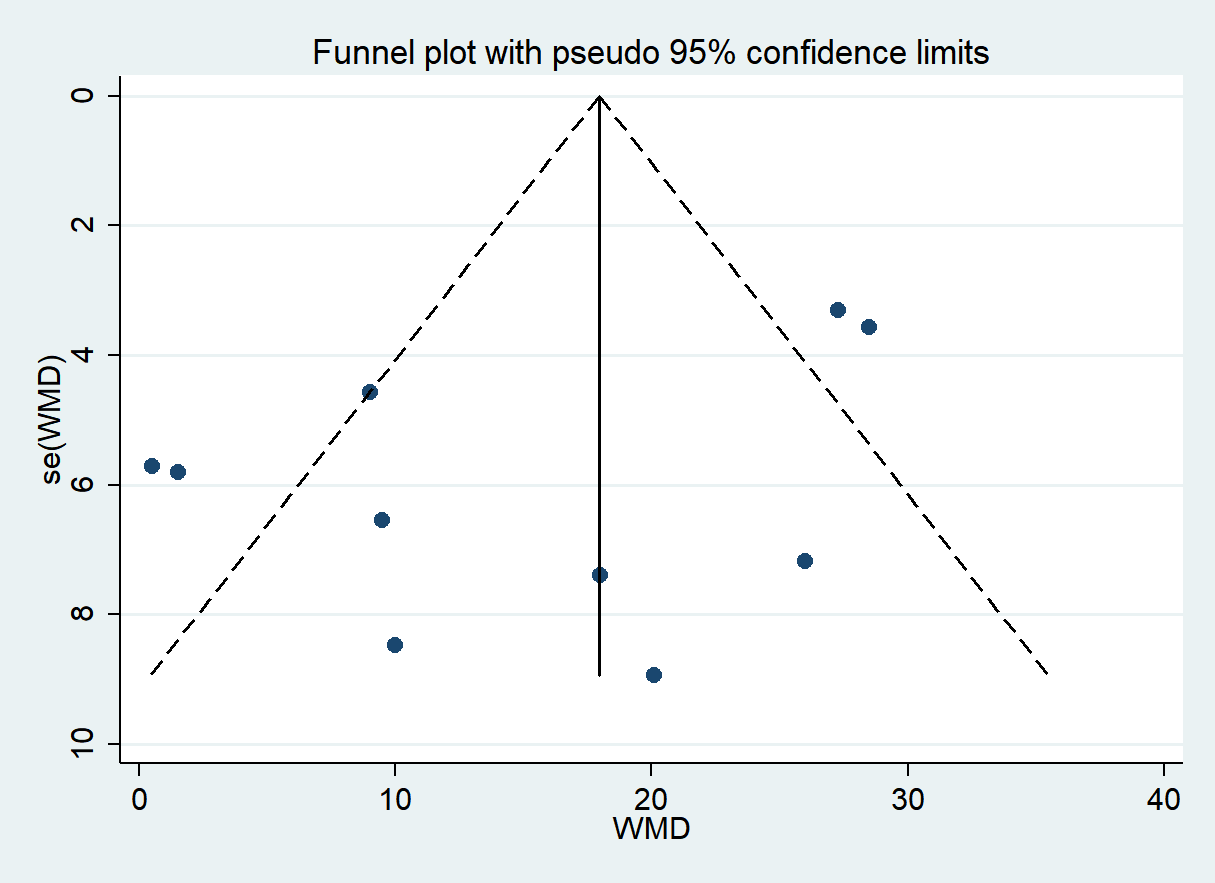


Supplementary Fig. 13. Publication bias heterogeneity funnel plot for pain intensity (short term follow-up). A funnel plot was used to assess the risk of publication bias. The diagonal lines represent the 95% confidence limits. Se: standard error, MD: mean difference. A random-effects model was used.


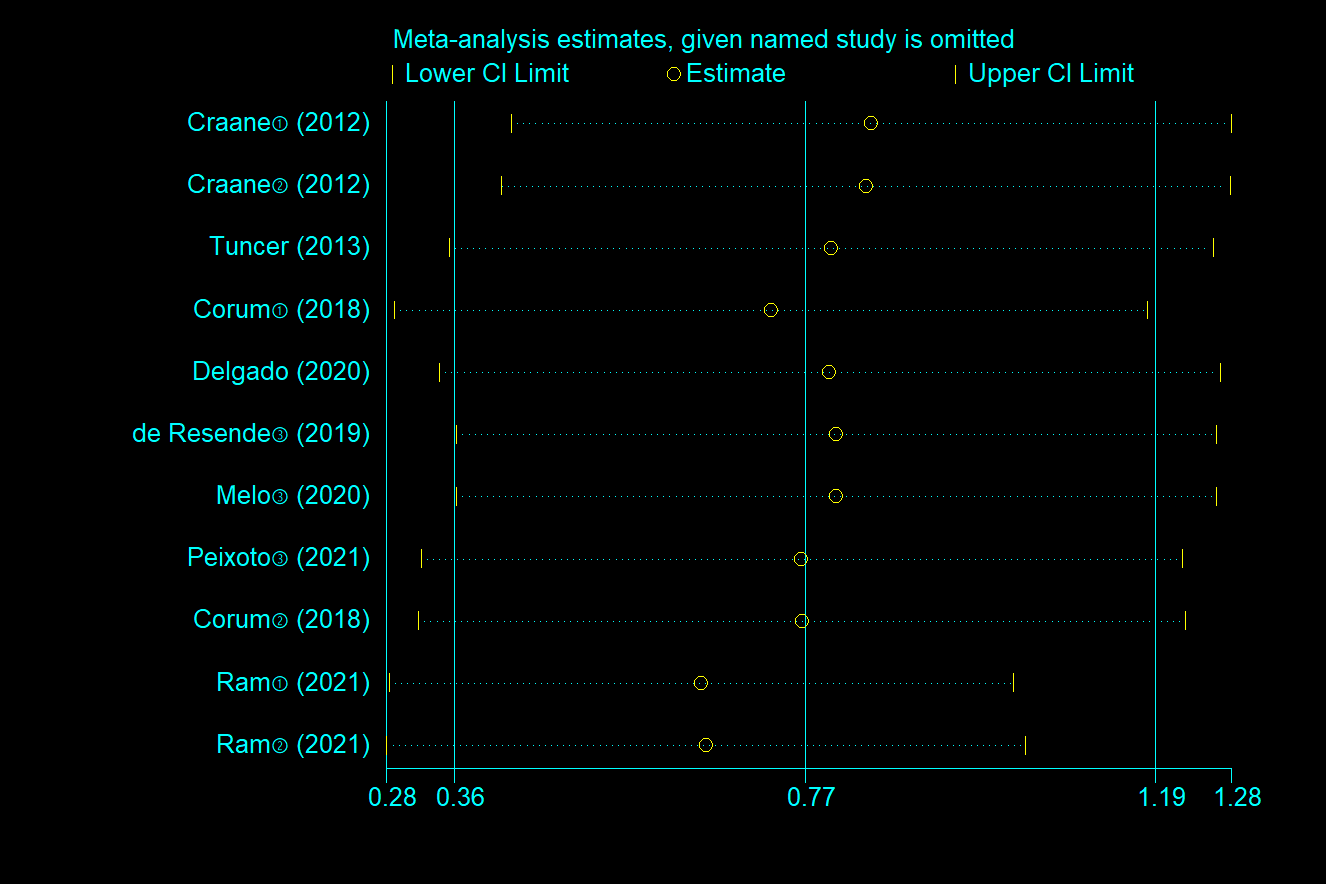


Supplementary Fig. 14. Exclusion sensitivity plot for pain. Comparing home-based rehabilitation with home-based rehabilitation plus manual therapy in pain (mean difference, MD) at short term follow-up. Pooled mean differences calculated by random-effects model.


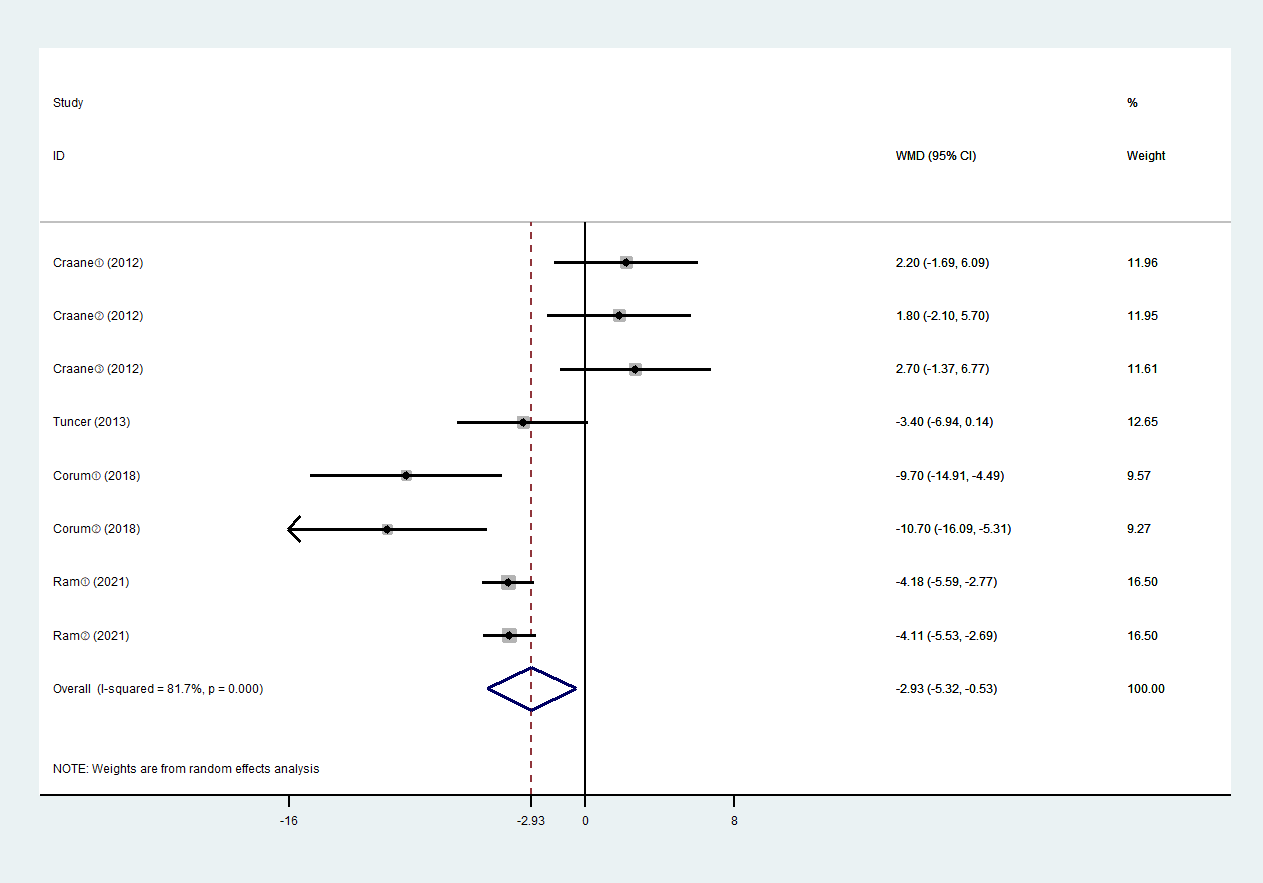


Supplementary Fig. 15. Forest plot of maximal mouth opening. Comparing home-based rehabilitation with home-based rehabilitation plus manual therapy in pain (mean difference, MD) at short term follow-up. Pooled mean differences calculated by random-effects model.


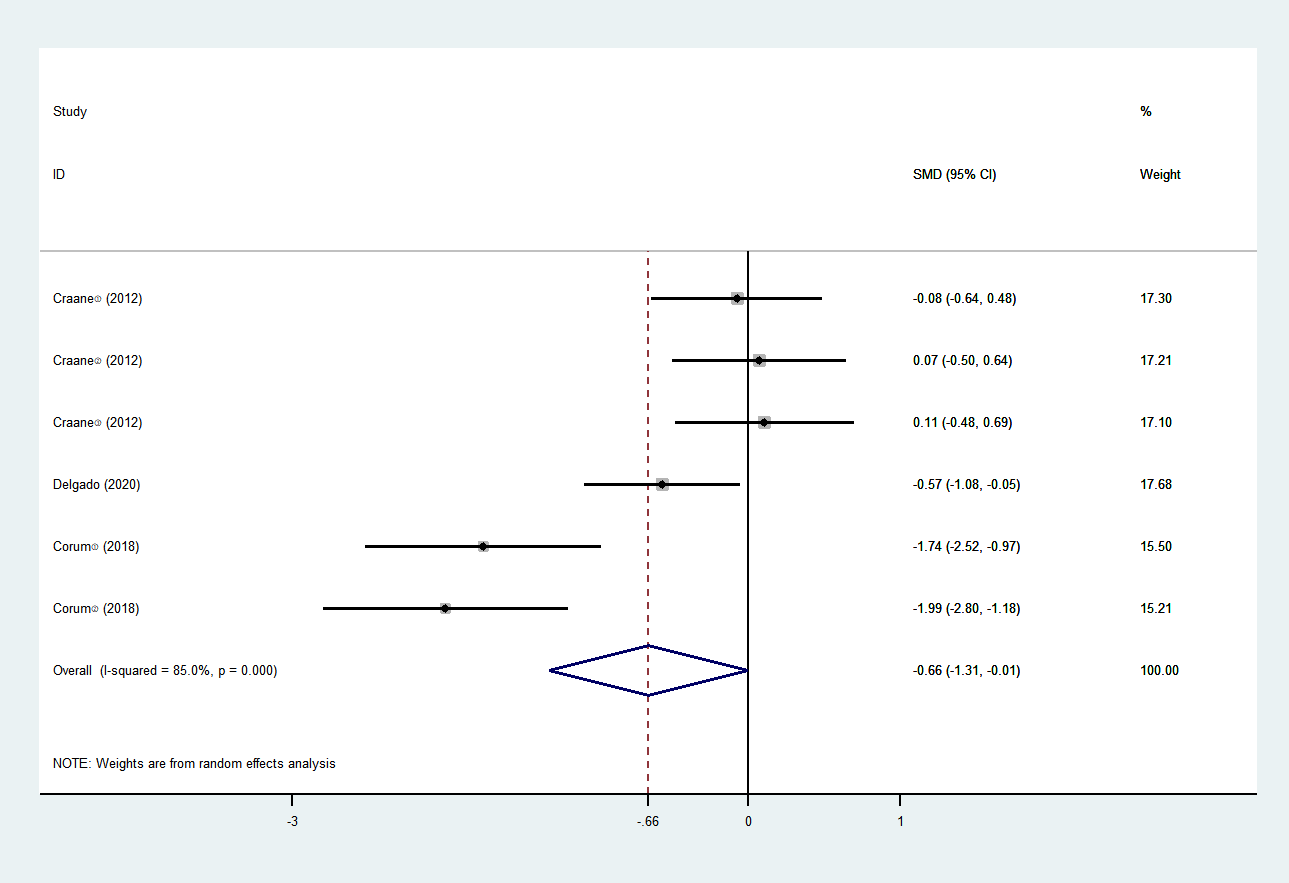


Supplementary Fig. 16. Forest plot of pressure pain thresholds-temporalis. Comparing home-based rehabilitation with home-based rehabilitation plus manual therapy in pain (standardized mean difference, SMD) at short term follow-up. Pooled mean differences calculated by random-effects model.


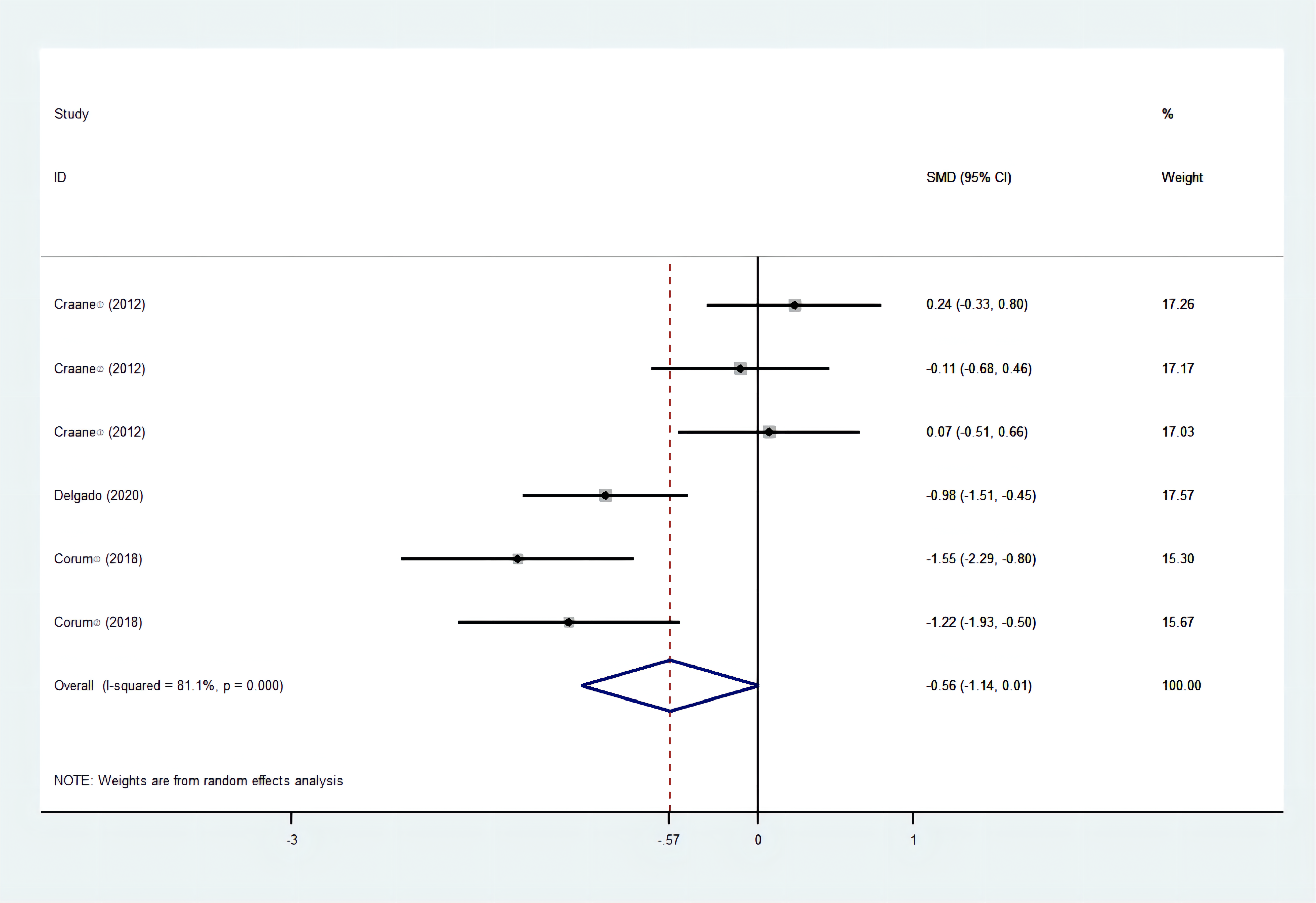


Supplementary Fig. 17. Forest plot of pressure pain thresholds-masseter. Comparing home-based rehabilitation with home-based rehabilitation plus manual therapy in pain (standardized mean difference, SMD) at short term follow-up. Pooled mean differences calculated by random-effects model.


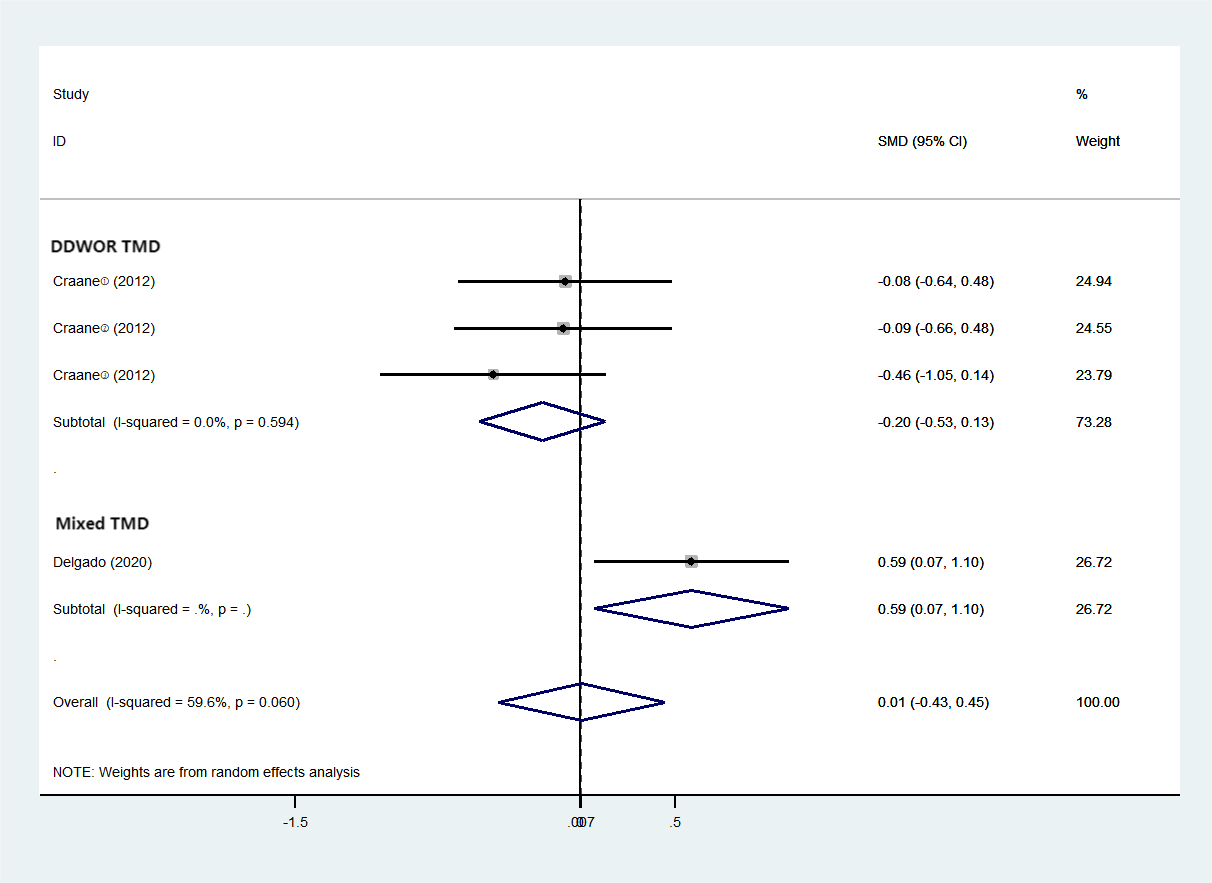


Supplementary Fig. 18. Forest plot of function at short term follow-up. Comparing home-based rehabilitation with home-based rehabilitation plus manual therapy in function (standardized mean difference, SMD) at short term follow-up. Pooled mean differences calculated by fixed effects model.


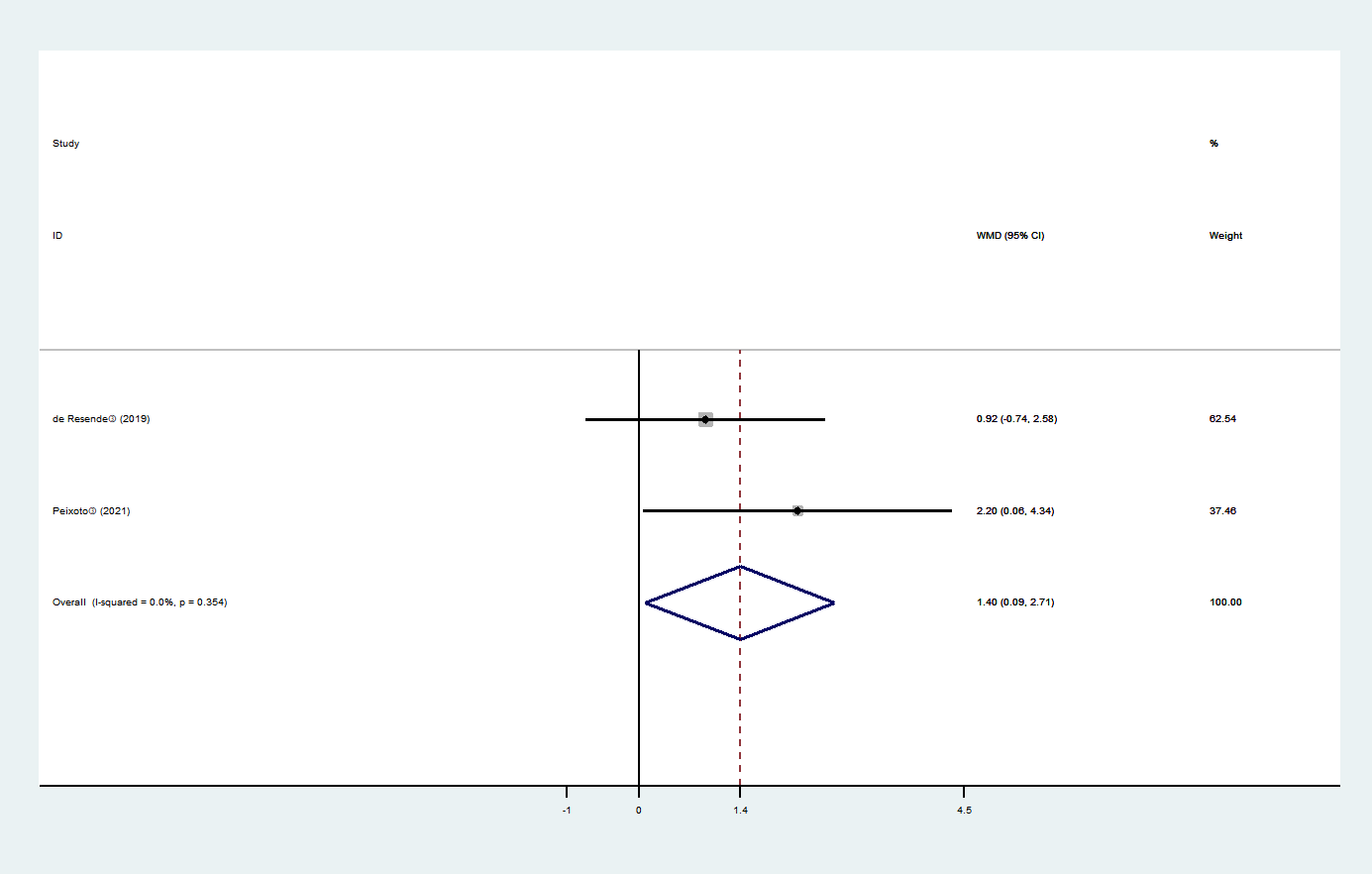


Supplementary Fig. 19. Forest plot of sleep quality at short term follow-up. Comparing home-based rehabilitation with home-based rehabilitation plus manual therapy in sleep quality (mean difference, MD) at short term follow-up. Pooled mean differences calculated by fixed effects model.


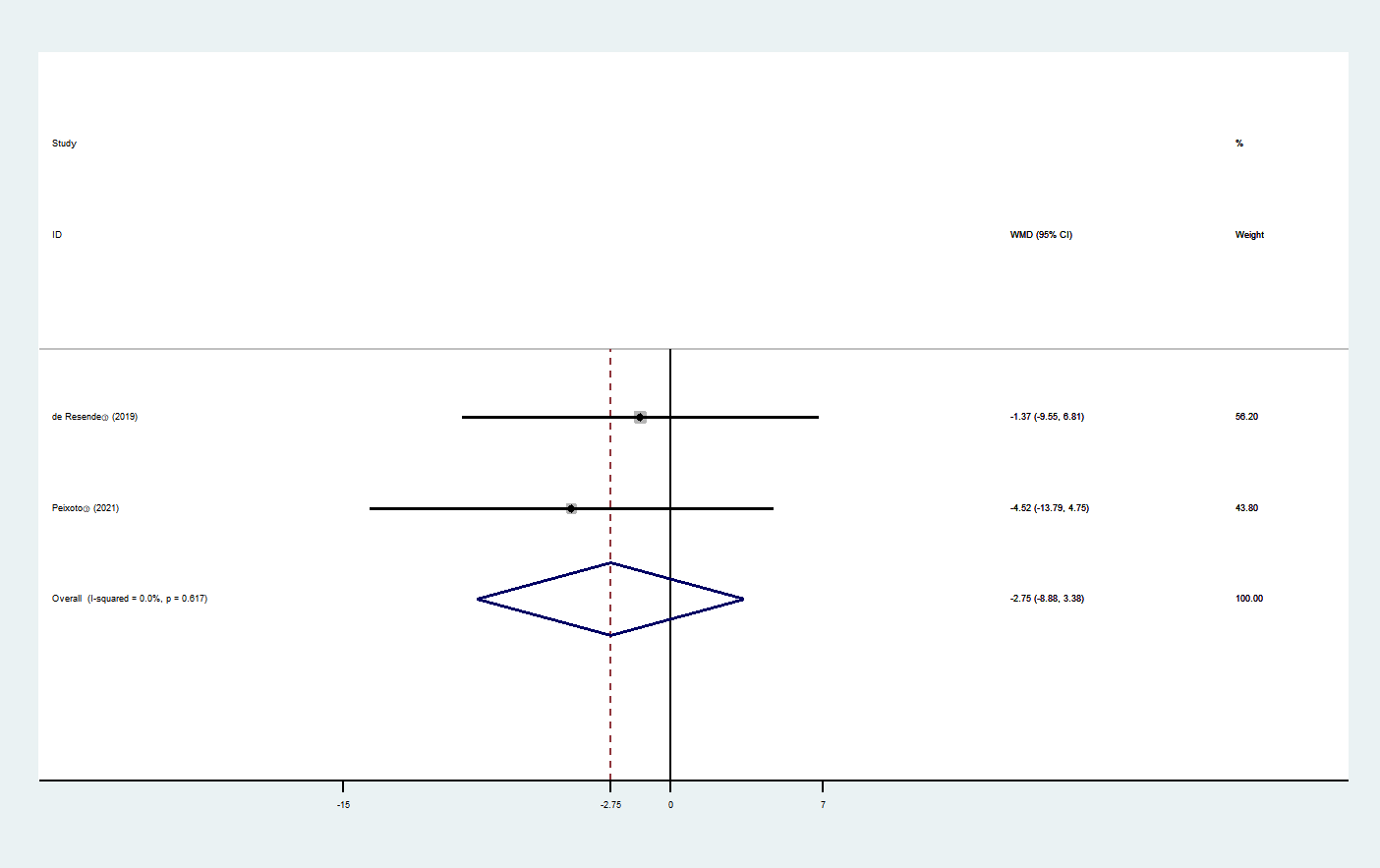


Supplementary Fig. 20. Forest plot of life quality (psychological) at short term follow-up. Comparing home-based rehabilitation with home-based rehabilitation plus manual therapy in quality of life (mean difference, MD) at short term follow-up. Pooled mean differences calculated by fixed effects model.


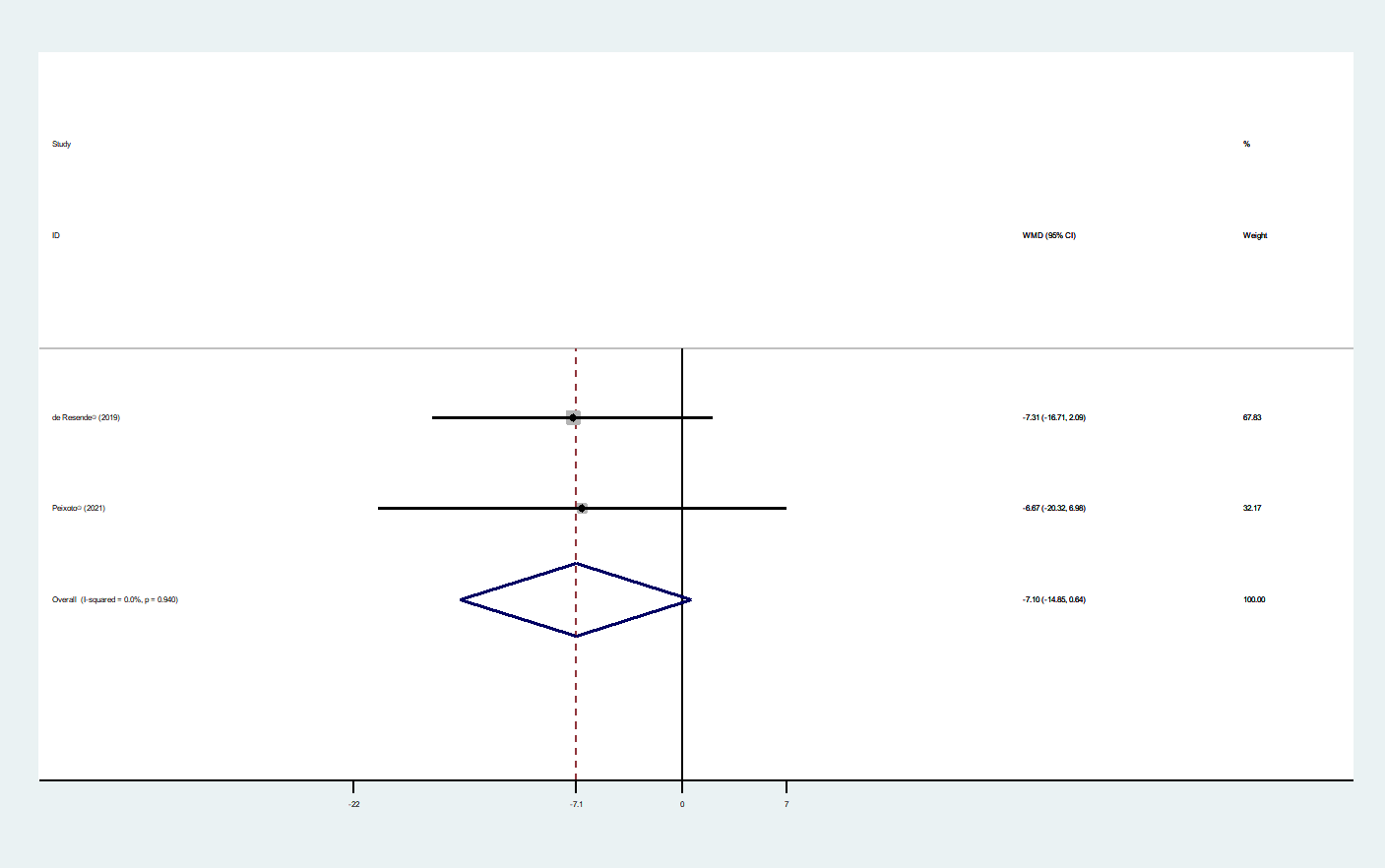


Supplementary Fig. 21. Forest plot of life quality (social) at short term follow-up. Comparing home-based rehabilitation with home-based rehabilitation plus manual therapy in quality of life (mean difference, MD) at short term follow-up. Pooled mean differences calculated by fixed effects model.


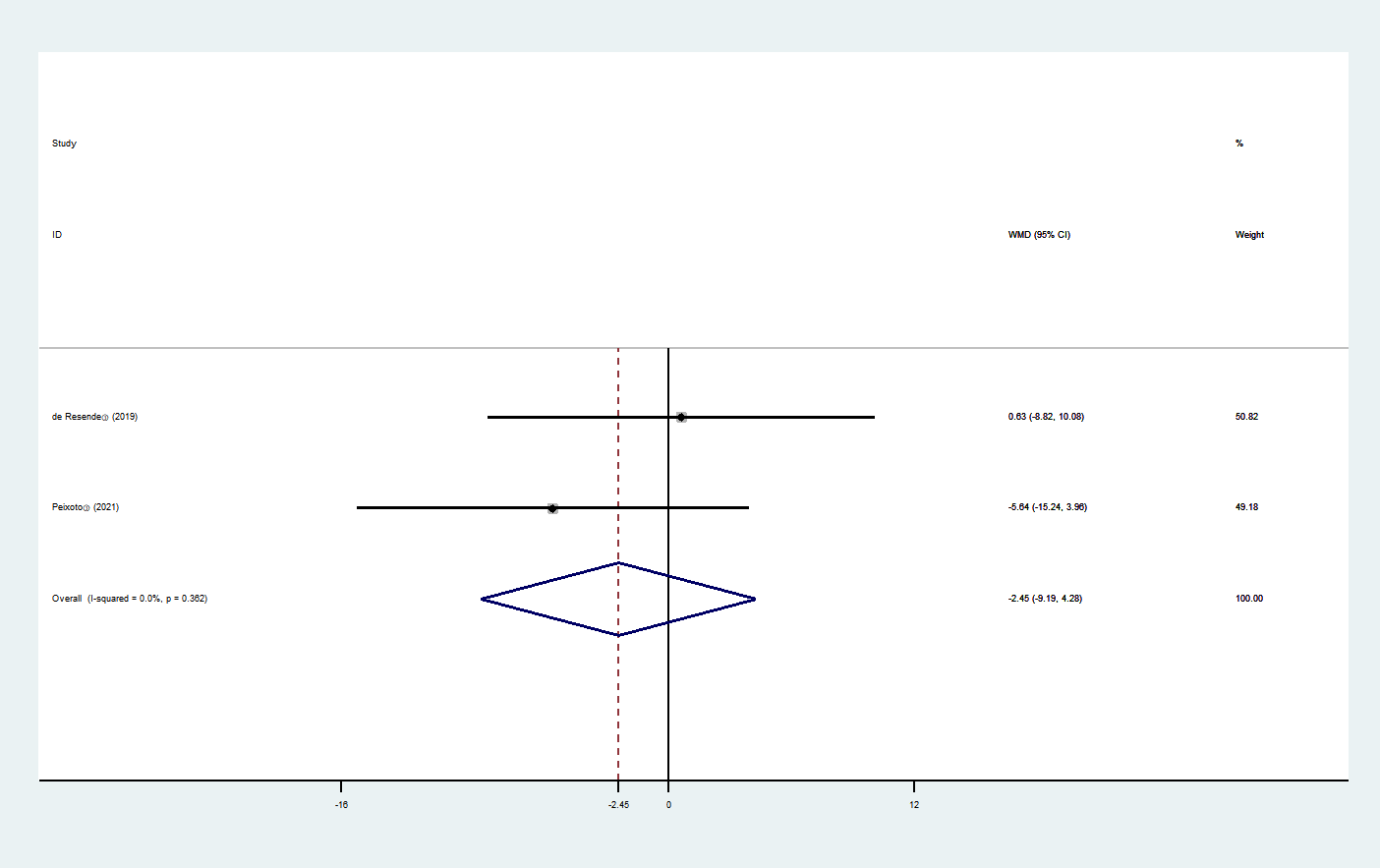


Supplementary Fig. 22. Forest plot of life quality (general) at short term follow-up. Comparing home-based rehabilitation with home-based rehabilitation plus manual therapy in quality of life (mean difference, MD) at short term follow-up. Pooled mean differences calculated by fixed effects model.


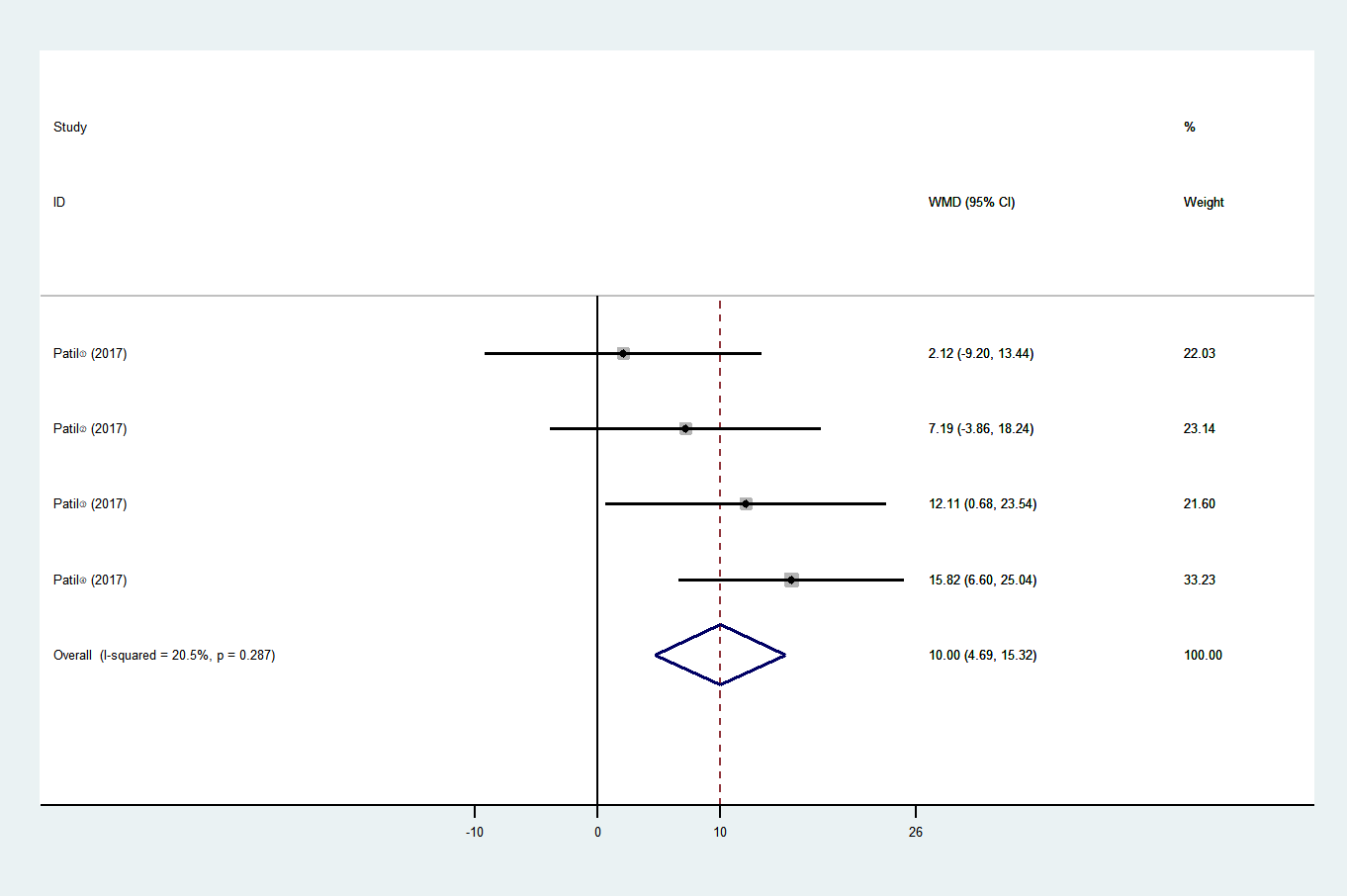


Supplementary Fig. 23. Forest plot of tenderness to palpation-masseter at short term follow-up. Comparing home-based rehabilitation with TENS in tenderness to palpation (mean difference, MD) at short term follow-up. Pooled mean differences calculated by fixed effects model.


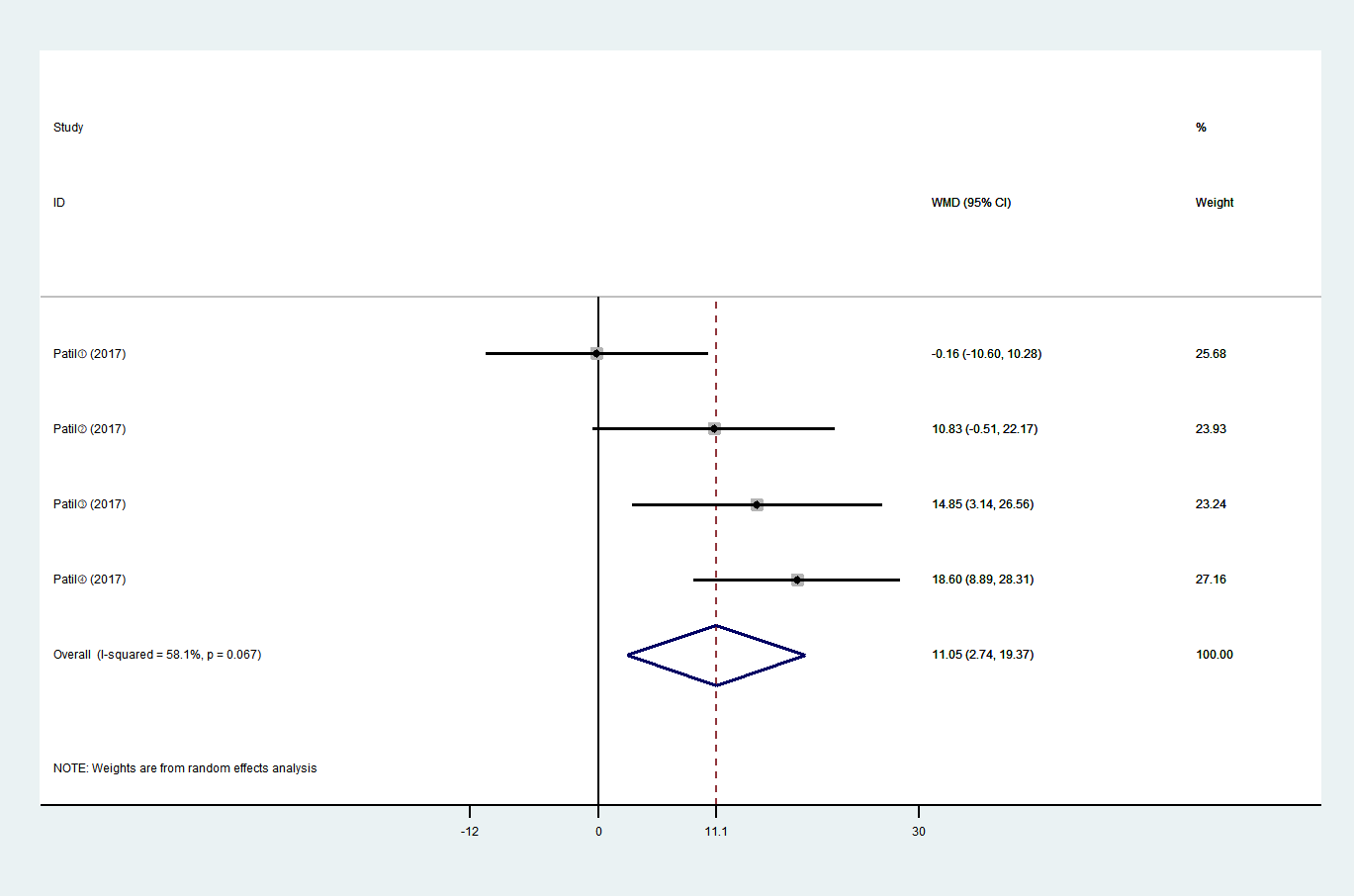


Supplementary Fig. 24. Forest plot of tenderness to palpation-temporomandibular joint at short term follow-up. Comparing home-based rehabilitation with TENS in tenderness to palpation (mean difference, MD) at short term follow-up. Pooled mean differences calculated by random effects model.


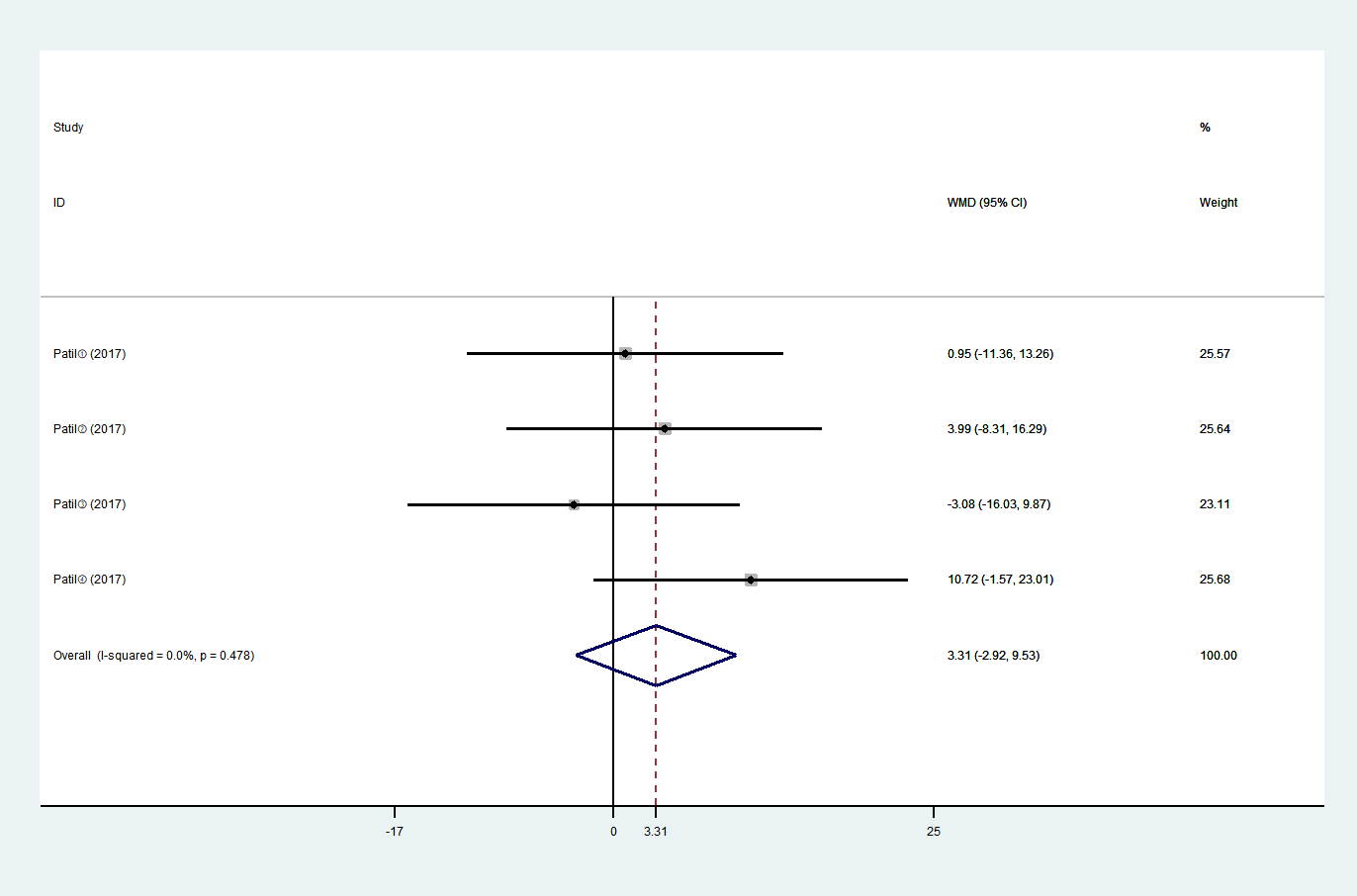


Supplementary Fig. 25. Forest plot of tenderness to palpation-temporalis at short term follow-up. Comparing home-based rehabilitation with TENS in tenderness to palpation (mean difference, MD) at short term follow-up. Pooled mean differences calculated by fixed effects model.


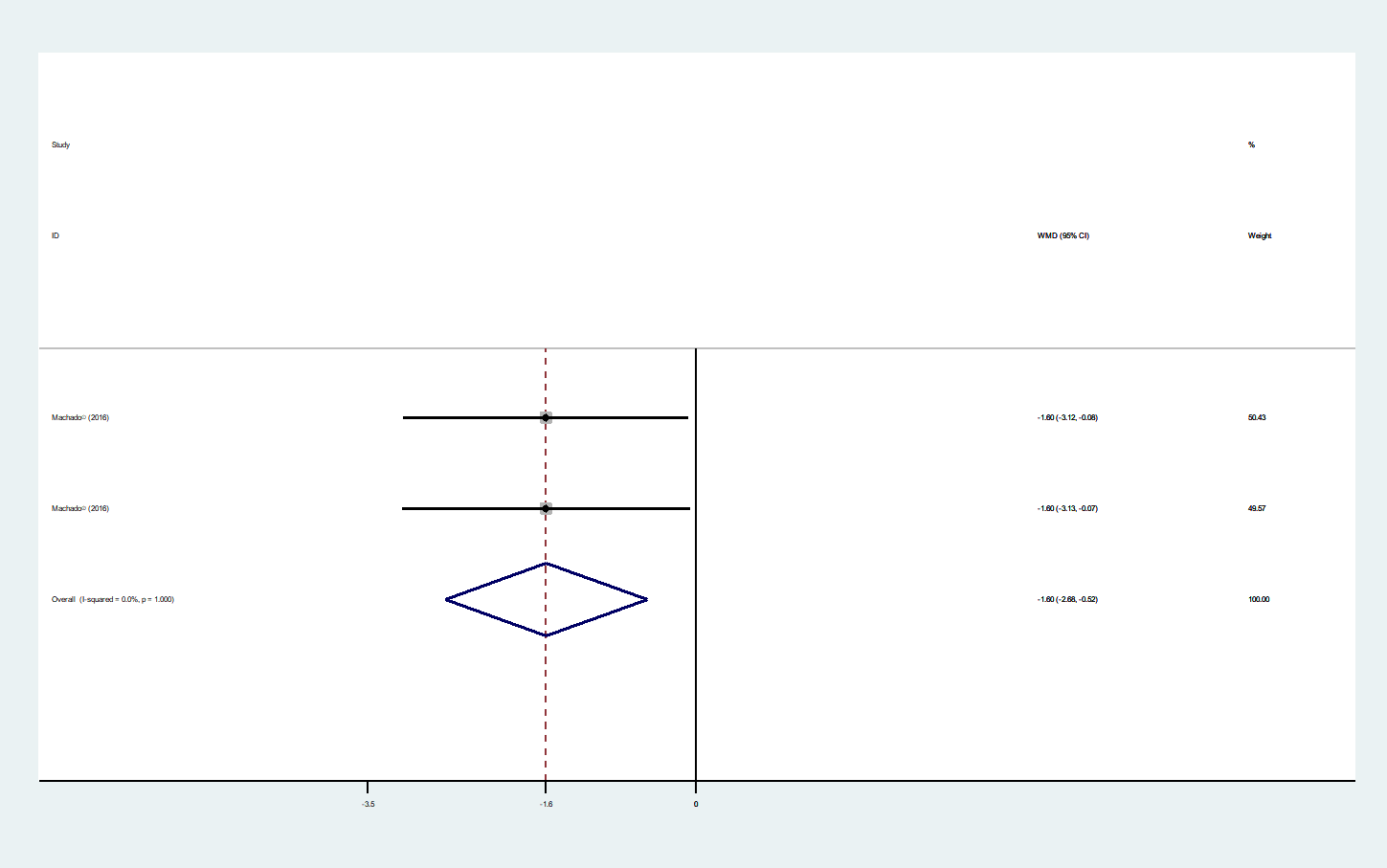


Supplementary Fig. 26. Forest plot of tenderness to palpation-masseter at intermediate term follow-up. Comparing home-based rehabilitation with LLLT in tenderness to palpation (mean difference, MD) at intermediate term follow-up. Pooled mean differences calculated by fixed effects model.


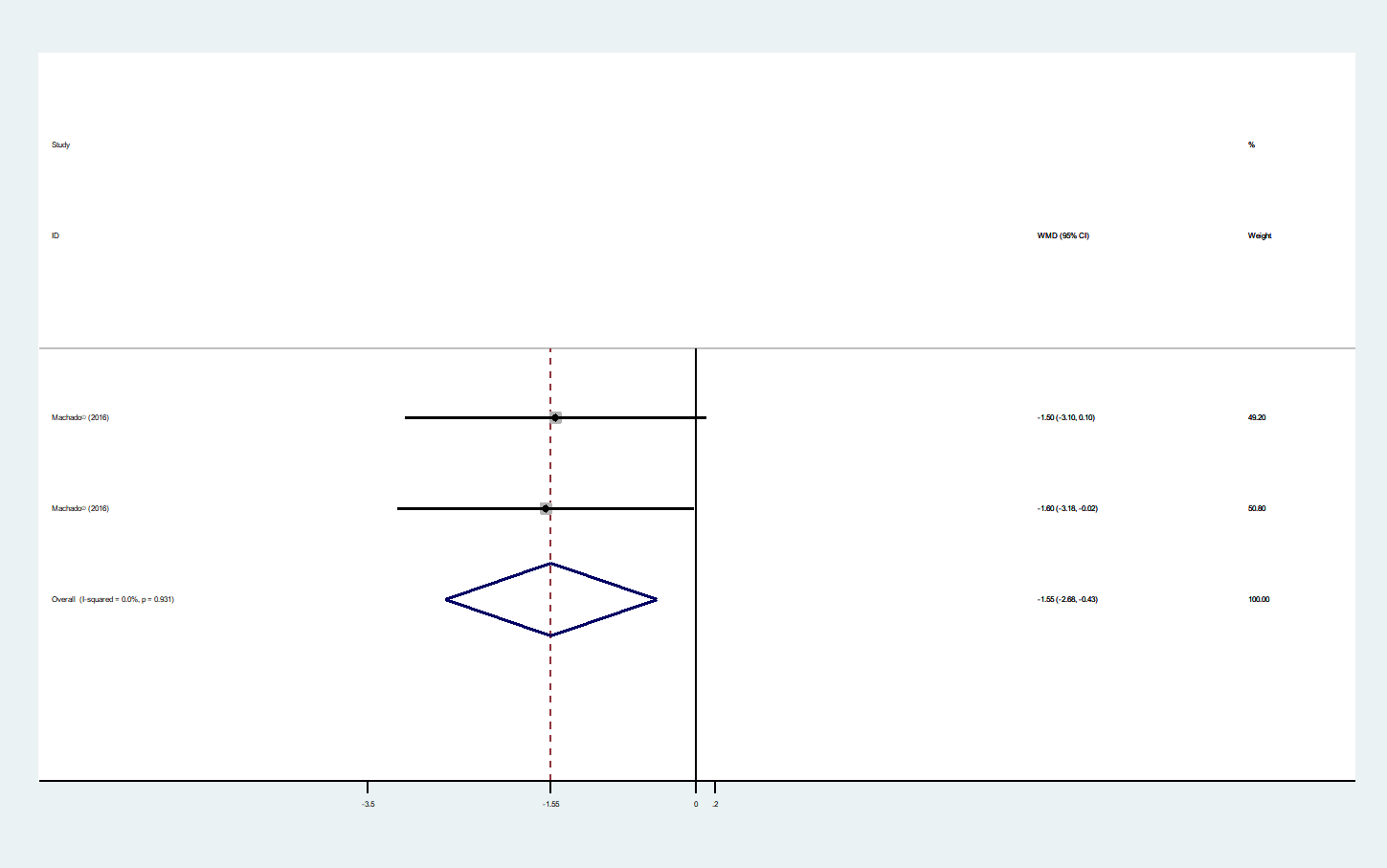


Supplementary Fig. 27. Forest plot of tenderness to palpation-temporomandibular joint at intermediate term follow-up. Comparing home-based rehabilitation with LLLT in tenderness to palpation (mean difference, MD) at intermediate term follow-up. Pooled mean differences calculated by fixed effects model.
